# Annexin A1 binds PDZ and LIM domain 7 to inhibit adipogenesis and prevent obesity

**DOI:** 10.1038/s41392-024-01930-0

**Published:** 2024-08-23

**Authors:** Lu Fang, Changjie Liu, Zong-zhe Jiang, Mengxiao Wang, Kang Geng, Yangkai Xu, Yujie Zhu, Yiwen Fu, Jing Xue, Wenxin Shan, Qi Zhang, Jie Chen, Jiahong Chen, Mingming Zhao, Yuxuan Guo, K. W. Michael Siu, Y. Eugene Chen, Yong Xu, Donghui Liu, Lemin Zheng

**Affiliations:** 1 The Institute of Cardiovascular Sciences and Institute of Systems Biomedicine, School of Basic Medical Sciences, State Key Laboratory of Vascular Homeostasis and Remodeling, NHC Key Laboratory of Cardiovascular Molecular Biology and Regulatory Peptides, Beijing Key Laboratory of Cardiovascular Receptors Research, Health Science Center, Peking University, 100191 Beijing, China; 2 Department of Blood Transfusion, Guangzhou First People’s Hospital, School of Medicine, South China University of Technology, Guangzhou, 510000 Guangdong China; 3 Department of Endocrinology and Metabolism, The Affiliated Hospital of Southwest Medical University, Luzhou, 646000 Sichuan PR China; 4 Metabolic Vascular Disease Key Laboratory of Sichuan Province, Luzhou, 646000 Sichuan China; 5 Department of plastic and burns surgery, The Affiliated Hospital of Southwest Medical University, Luzhou, 646000 Sichuan PR China; 6 Beijing Tiantan Hospital, China National Clinical Research Center for Neurological Diseases, Advanced Innovation Center for Human Brain Protection, Capital Medical University, 6 Tiantan Xili, Chongwen District, 100050 Beijing, China; 7 Department of Cardiology and Institute of Vascular Medicine, Peking University Third Hospital; State Key Laboratory of Vascular Homeostasis and Remodeling, Peking University; NHC Key Laboratory of Cardiovascular Molecular Biology and Regulatory Peptides; Beijing Key Laboratory of Cardiovascular Receptors Research; Research Unit of Medical Science Research Management/Basic and Clinical Research of Metabolic Cardiovascular Diseases, Chinese Academy of Medical Sciences, 100191 Beijing, China; 8 Center for Mass Spectrometry Research and Clinical Application, Shandong Public Health Clinical Center Affiliated to Shandong University, Lishan Campus, 46 Lishan Road, Jinan, Shandong China; 9 Department of Chemistry and Biochemistry, University of Windsor, 401 Sunset Avenue, Windsor, ON N9B 3P4 Canada; 10 Department of Internal Medicine, University of Michigan Medical Center, Ann Arbor, MI USA; 11 Department of Geriatrics, National Key Clinical Specialty, Guangzhou First People’s Hospital, School of Medicine, South China University of Technology, Guangzhou, 510000 China

**Keywords:** Differentiation, Disease model, Endocrine system and metabolic diseases

## Abstract

Obesity is a global issue that warrants the identification of more effective therapeutic targets and a better understanding of the pivotal molecular pathogenesis. Annexin A1 (ANXA1) is known to inhibit phospholipase A2, exhibiting anti-inflammatory activity. However, the specific effects of ANXA1 in obesity and the underlying mechanisms of action remain unclear. Our study reveals that ANXA1 levels are elevated in the adipose tissue of individuals with obesity. Whole-body or adipocyte-specific ANXA1 deletion aggravates obesity and metabolic disorders. ANXA1 levels are higher in stromal vascular fractions (SVFs) than in mature adipocytes. Further investigation into the role of ANXA1 in SVFs reveals that ANXA1 overexpression induces lower numbers of mature adipocytes, while ANXA1-knockout SVFs exhibit the opposite effect. This suggests that ANXA1 plays an important role in adipogenesis. Mechanistically, ANXA1 competes with MYC binding protein 2 (MYCBP2) for interaction with PDZ and LIM domain 7 (PDLIM7). This exposes the MYCBP2-binding site, allowing it to bind more readily to the SMAD family member 4 (SMAD4) and promoting its ubiquitination and degradation. SMAD4 degradation downregulates peroxisome proliferator-activated receptor gamma (PPARγ) transcription and reduces adipogenesis. Treatment with Ac2-26, an active peptide derived from ANXA1, inhibits both adipogenesis and obesity through the mechanism. In conclusion, the molecular mechanism of ANXA1 inhibiting adipogenesis was first uncovered in our study, which is a potential target for obesity prevention and treatment.

## Introduction

Obesity poses a global health challenge,^1^ linked to chronic conditions like type 2 diabetes, cardiovascular diseases,^2,3^ and cancer.^4^ The 2017 Global Burden of Disease Study^5^ reported that 13% of adults are obese, with 39% being overweight. Additionally, a quarter of children and adolescents worldwide are overweight.^4^ In 2019, obesity contributed to 160.2 million disability-adjusted life years.^6^ Obesity results from an energy intake-expenditure imbalance,^7^ with various cells contributing to excessive and dysfunctional accumulation of adipose tissue through multiple mechanisms.^8^ In individuals with obesity, adipocytes undergo hypertrophy, hyperplasia, and abnormal differentiation,^9^ leading to insulin resistance and impaired glucose and lipid metabolism, heightening the risk of chronic illnesses.^10^

Numerous studies have emphasized that adipose tissue serves not only as an energy reservoir but also as a crucial endocrine regulator, influencing metabolic processes and inflammatory responses. Dysregulation of lipid metabolism in adipocytes can impair function and contribute to various diseases.^11^ Adipose tissue, characterized by remarkable plasticity in expansion and contraction, results from coordinated interactions among various cell types. It comprises mature adipocytes and stromal vascular fractions (SVFs), including adipocyte progenitor cells (APCs) capable of proliferation and differentiation into mature adipocytes. Adipose tissue expansion can occur due to increased adipocyte numbers (hyperplasia) or enlarged resident adipocytes in white adipose tissue (WAT). Both processes are dynamic and significantly influenced by dietary factors. Adipogenesis, the process by which adipocytes form from preadipocytes, plays a pivotal role in the pathogenesis of obesity and related disorders.^12^ The process is important for understanding obesity, because during prolonged caloric excess, new adipocytes can emerge from adipogenesis and contribute to adipose tissue expansion.^13^ Adipogenesis is regulated by numerous transcription factors, including CCAAT/enhancer-binding protein beta (C/EBPβ), CCAAT/enhancer-binding protein delta (C/EBPδ), peroxisome proliferator-activated receptor gamma (PPARγ), and CCAAT/enhancer-binding protein alpha (C/EBPα). These transcription factors coordinate the expression of hundreds of genes to establish the mature adipocyte phenotype.^14^ Notably, Bone morphogenetic protein 4 (BMP4) plays a pivotal role in inducing mesenchymal progenitor cells to differentiate into the adipocyte lineage. Several studies confirm that BMP4 is sufficient to drive adipocyte migration and is essential for in vitro adipogenesis. Upon binding to its receptor, BMP4 activates downstream transcription factor SMAD family member 4 (SMAD4), which, in turn, stimulates the transcription of PPARγ, thereby promoting terminal differentiation of preadipocytes. Additionally, BMP4 has been found to activate the dissociation of the WNT1-inducible-signaling pathway protein 2 / Zinc finger protein 423 (WISP2/ZNF423) complex via SMAD1/5/8, allowing ZNF423 nuclear translocation and subsequent induction of PPARγ. These findings highlight the close association between SMAD family proteins and adipogenesis.^15^ A comprehensive investigation into the mechanisms of adipogenesis is crucial for countering the prevailing obesity epidemic. Despite extensive research on preadipocytes and progenitor cells contributing to mature adipocytes, our understanding of their origin and properties within the body remains incomplete.

Annexin A1 (ANXA1), a member of the calcium-dependent phospholipid-binding annexin superfamily, has a molecular weight of 37 kDa. Initially identified as a downstream signaling molecule of glucocorticoids in 1979, the gene sequence encoding ANXA1 was cloned in 1986.^16^ Since then, research on ANXA1 has steadily increased, leading to a deeper understanding of its multifaceted biological functions.^13^ Notably, ANXA1 is involved in anti-inflammatory responses, apoptosis regulation, and cell cycle modulation. It is expressed in most cell types^17^ and functions extracellularly as an anti-inflammatory pro-resolving protein.^18^ It exerts protective effects against several diseases, such as viral infections,^19^ breast cancer,^20^ pancreatic cancer,^21^ and glioblastoma.^22^ ANXA1 is notably increased in the adipose tissue of children with obesity^23^ and in mice fed with a high-fat diet (HFD).^24^ Additionally, treatment with recombinant human ANXA1 reportedly reduces body weight in such mice.^25^ These findings suggest that ANXA1 plays an important role in regulating adiposity and metabolic diseases associated with obesity.^26^ However, most of existing studies on the anti-obesity effects of ANXA1 are based on its anti-inflammatory properties and focus on endothelial cells and inflammatory cells. These studies have not considered the role of ANXA1 in adipogenesis, and the underlying molecular mechanism remains unclear. PDZ and LIM domain 7 (PDLIM7) is a PDZ-LIM domains-containing protein that functions as a ubiquitin E3 ligase and inhibits NF-κB-mediated inflammatory responses.^27^ According to previous studies, PDLIM7 can promote YAP1 nuclear translocation and the formation of YAP1-TEAD complexes to activate the Hippo pathway by specifically binding to the PDZ-binding motif of YAP1 through its PDZ domain. This pathway is involved in the transcriptional regulation of various genes, including those related to cell survival, proliferation, cell cycle, migration, and motility.^28^ MYC binding protein 2 (MYCBP2) is a ubiquitin E3 ligase that is essential for neural development and regulates axon maintenance.^29^ MYCBP2 has also been shown to ubiquitinate F-Box and WD Repeat Domain Containing 7 (Fbxw7) and Unc-51 Like Autophagy Activating Kinase 1 (ULK1), thereby promoting chemoresistance and inhibiting neuronal autophagy, respectively.^30,31^ However, the roles of PDLIM7 and MYCBP2 in obesity and fat metabolism were not clear.

Herein, we focused on purified preadipocytes and have elucidated the anti-obesity role of ANXA1, PDLIM7 and MYCBP2 in the suppression of adipogenesis, along with the underlying molecular mechanisms. This study provides a new avenue for research in this field and will facilitate the development of new therapeutic strategies for obesity-related metabolic diseases.

## Results

### ANXA1 knockout aggravates obesity and metabolic disorders in HFD-fed mice

**Fig. 1:**
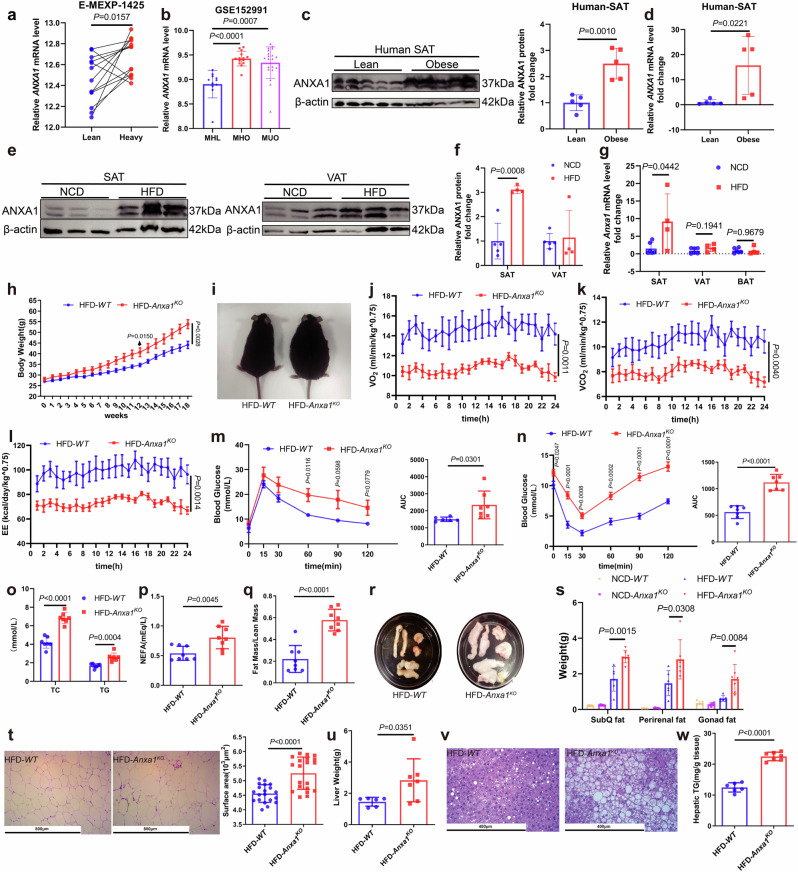
The expression of *ANXA1* is increased in adipose tissue in human with obesity, obesity and metabolic disorders worsen in ANXA1 knockout mice fed with HFD. **a** Gene expression analysis of *ANXA1* was performed in subcutaneous adipose tissue (SAT) from 13 pairs of twins (E-MEXP-1425). Student’s *t* test was used for analysis. **b** Transcriptome analysis of *ANXA1* was performed in SAT from metabolically healthy lean (MHL, *n* = 11), metabolically healthy obese (MHO, *n* = 14), and metabolically unhealthy obese (MUO, *n* = 20) individuals (GSE152991). Student’s *t* test was used for analysis. **c** Representative western blotting and quantification of ANXA1 from SAT of lean and obese individuals (*n* = 5 per group). Student’s *t* test was used for analysis. **d** mRNA abundance of *ANXA1* in SAT of lean and obese individuals (*n* = 5 per group). Student’s *t* test was used for analysis. Representative western blotting (**e**) and quantification (**f**) of ANXA1 from SAT and VAT of *WT* mice fed with NCD or HFD for 12 weeks (*n* = 4–5 per group). Student’s *t* test was used for analysis. **g** mRNA abundance of *Anxa1* in SAT, VAT and Brown Adipose Tissue (BAT) of *WT* mice fed with NCD or HFD for 12 weeks (*n* = 4–6 per group). One-way ANOVA and Dunn post hoc test were used for analysis. **h** Average body weight ± s.e.m. of *WT* and *Anxa1*^*KO*^ mice fed with HFD for 18 weeks (*n* = 8 per group). One-way ANOVA and Dunn post hoc test were used for analysis. **i** Representative photographs of *WT* and *Anxa1*^*KO*^ mice fed with HFD for 12 weeks (*n* = 8 per group). **j**–**l** Oxygen consumption, carbon dioxide production, and energy expenditure within 24 h of *WT* and *Anxa1*^*KO*^ mice fed with HFD for 12 weeks (*n* = 5 per group). VO_2_ oxygen consumption, VCO_2_ carbon dioxide production, EE energy expenditure. One-way ANOVA and Dunn post hoc test were used for analysis. **m** Results of glucose tolerance test (GTT)(left) were quantified as area under the curve (AUC) (right) for *WT* and *Anxa1*^*KO*^ mice fed with HFD for 12 weeks (*n* = 5–7 per group). One-way ANOVA and Dunn post hoc test were used for analysis. **n** Results of insulin tolerance test (ITT) (left) were quantified as AUC (right) for *WT* and *Anxa1*^*KO*^ mice fed with HFD for 12 weeks (*n* = 7 per group). One-way ANOVA and Dunn post hoc test were used for analysis. **o** Plasma total cholesterol (TC) and triglyceride (TG) concentrations in *WT* and *Anxa1*^*KO*^ mice fed with HFD for 12 weeks (*n* = 7 per group). One-way ANOVA and Dunn post hoc test were used for analysis. **p** Plasma non-esterified fatty acid (NEFA) concentration in *WT* and *Anxa1*^*KO*^ mice fed with HFD for 12 weeks (*n* = 8 per group). Student’s *t* test was used for analysis. **q** Ratio of fat mass to lean mass was detected by magnetic resonance imaging in *WT* and *Anxa1*^*KO*^ mice fed with HFD for 12 weeks (*n* = 8 per group). Student’s *t* test was used for analysis. **r** Representative photographs of subcutaneous fat(upper left), perirenal fat(lower), and gonadal fat(upper right) from *WT* and *Anxa1*^*KO*^ mice fed with HFD for 12 weeks (*n* = 8 per group). **s** Subcutaneous fat, perirenal fat, and gonadal fat weight of *WT* and *Anxa1*^*KO*^ mice fed with NCD or HFD for 12 weeks (*n* = 5–7 per group). One-way ANOVA and Dunn post hoc test were used for analysis. **t** Hematoxylin & eosin-stained subcutaneous fat sections showing adipocyte size in *WT* and *Anxa1*^*KO*^ mice fed with HFD for 12 weeks (*n* = 20 per group). Scale bar: 800 µm. Student’s *t* test was used for analysis. **u** Liver weight of *WT* and *Anxa1*^*KO*^ mice fed with NCD or HFD for 12 weeks (*n* = 6–7 per group). Student’s *t* test was used for analysis. **v** Hematoxylin & eosin-stained liver sections from *WT* and *Anxa1*^*KO*^ mice fed with HFD for 12 weeks. Scale bar: 400 µm. **w** Hepatic TG concentration in *WT* and *Anxa1*^*KO*^ mice fed with HFD for 12 weeks (*n* = 6–7 per group). Student’s *t* test was used for analysis

The development of obesity is influenced by a combination of genetic and environmental factors, making it a complex condition. Studies on twins offer valuable insights into the acquired nature of obesity.^32^ We first assessed whether there were differences in *ANXA1* mRNA levels in the subcutaneous adipose tissue (SAT) between monozygotic co-twins discordant for body mass index (BMI) (within-pair difference in BMI ≥ 3 kg·m^−2^). In two publicly available data sets of 13^32^ (Fig. 1a) and 49^33^ (FDR, *P* = 0.00383) twins pairs, gene expression analyses identified a significant increase in *ANXA1* mRNA levels in the SAT of identical twins with higher BMI. Additionally, in another cohort consisting of 45 individuals, *ANXA1* mRNA levels were elevated markedly in the SAT of both metabolically healthy and unhealthy obese individuals compared to metabolically healthy lean individuals^34^ (Fig. 1b).

Concurrently, SAT from five human subjects was analyzed, revealing a significant elevation of ANXA1 protein and *ANXA1* mRNA levels in obese subjects compared to their lean counterparts (Fig. 1c, d). In the SAT of wild-type (*WT*)-C57BL/6 mice fed with HFD for 12 weeks, the protein and mRNA levels of ANXA1 were higher than in those fed with normal chow diet (NCD), whereas no significant difference was observed in visceral adipose tissue (VAT) and brown adipose tissue (BAT) (Fig. 1e–g). The findings indicated a correlation between the elevated adipose tissue ANXA1 levels and obesity in humans and mice. ANXA1 protein levels in the heart and liver of HFD mice were unaltered compared with those in the NCD mice (Supplementary Fig. 1a, b). Correspondingly, we set forth to better understand the role of ANXA1 in adipose tissue than in other tissues in obesity.

*Anxa1* knockout (*Anxa1*^*KO*^) C57BL/6 mice were generated by microinjection of transcription activator-like effector nucleases (TALENs) into fertilized oocytes. Eight-week-old *WT* and *Anxa1*^*KO*^ mice were fed with either NCD or HFD for 12 or 18 weeks (Supplementary Fig. 1c). HFD-*Anxa1*^*KO*^ mice exhibited a significant increase in body weight compared to HFD-*WT* mice (Fig. 1h, i, Supplementary Fig. 1d). However, no significant difference in body weight was noted between NCD-*Anxa1*^*KO*^ and NCD-*WT* mice (Supplementary Fig. 1e).

Additionally, oxygen consumption (VO_2_), carbon dioxide production (VCO_2_), and energy expenditure (EE) decreased significantly in HFD-*Anxa1*^*KO*^ mice compared to those in HFD-*WT* mice (Fig. 1j–l), indicating a decline in the overall metabolic rate. No difference in basal blood glucose levels was observed between NCD-*Anxa1*^*KO*^ and NCD-*WT* mice (Supplementary Fig. 1f). However, the basal blood glucose and insulin levels were elevated significantly in HFD-*Anxa1*^*KO*^ mice (Supplementary Fig. 1f, g). HFD-*Anxa1*^*KO*^ mice displayed marked glucose intolerance (Fig. 1m) and severe insulin resistance (Fig. 1n) compared to HFD-*WT* mice. Plasma levels of total cholesterol (TC), triglyceride (TG), non-esterified fatty acid (NEFA), high-density lipoprotein cholesterol (HDL-C), and low-density lipoprotein cholesterol (LDL-C) were increased significantly in HFD-*Anxa1*^*KO*^ mice compared to those in HFD-*WT* mice (Fig. 1o, p and Supplementary Fig. 1h, i). These results established that ANXA1 knockout promoted HFD-induced obesity and metabolic disorder. Analysis of adipose and liver tissues in mice revealed that the ratio of fat-to-lean tissue was elevated significantly in HFD-*Anxa1*^*KO*^ mice (Fig. 1q), along with increased weights of subcutaneous, perirenal and gonadal fat (Fig. 1r, s) compared with those in HFD-*WT* mice. No significant changes in adipose tissue weight were observed upon NCD feeding (Fig. 1s). Finally, SAT lipid content, liver weight, and hepatic TG levels in HFD-*Anxa1*^*KO*^ mice were notably higher than those in HFD-*WT* mice (Fig. 1t–w). The data demonstrate that the knockout of ANXA1 promotes HFD-induced obesity and metabolic disorders in mice, and underscore the critical contribution of ANXA1 in the development of obesity.

### Adipose tissue-specific ANXA1 KO exacerbates obesity and metabolic disorders in HFD-fed mice

**Fig. 2:**
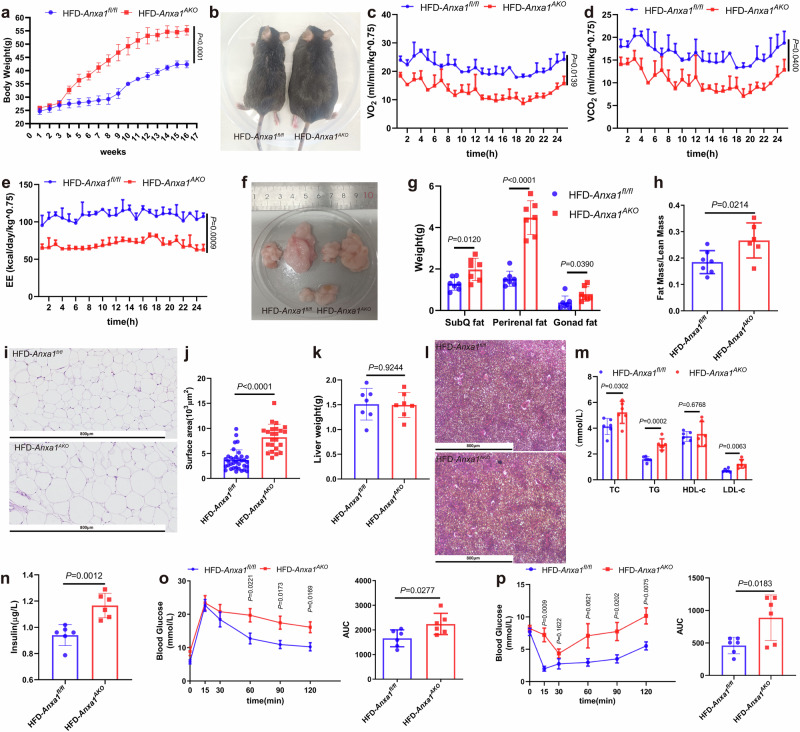
Adipose tissue-specific deficiency of ANXA1 aggravates obesity and insulin resistance in HFD mice. **a**–**p** *Anxa1*^*fl/fl*^ and adipose tissue-specific *Anxa1* knockout (*Anxa1*^*AKO*^) mice were fed with HFD for 16 weeks. **a** Average body weight ± s.e.m. of the mice (*n* = 5 per group). One-way ANOVA and Dunn post hoc test were used for analysis. **b** Representative photographs of the mice (*n* = 5–8 per group). **c**–**e** Oxygen consumption, carbon dioxide production, and energy expenditure within 24 h of the mice (*n* = 3 per group). One-way ANOVA and Dunn post hoc test were used for analysis. **f** Representative photographs of subcutaneous fat (upper left), perirenal fat (upper right), and gonadal fat (lower) from the mice (*n* = 5–8 per group). **g** Subcutaneous fat, perirenal fat, and gonadal fat weight of the mice (*n* = 7 per group). One-way ANOVA and Dunn post hoc test were used for analysis. **h** Ratio of fat mass to lean mass was detected by magnetic resonance imaging in the mice (*n* = 6–7 per group). Student’s *t* test was used for analysis. **i** Hematoxylin & eosin-stained subcutaneous fat sections from the mice (*n* = 5–8 per group). Scale bar: 800 µm. **j** Adipocyte size in subcutaneous fat from the mice (*n* = 23–35 per group). Student’s *t* test was used for analysis. **k** Liver weight of the mice (*n* = 6 per group). Student’s *t* test was used for analysis. **l** Hematoxylin & eosin-stained liver sections from the mice (*n* = 5-8 per group). Scale bar: 800 µm. **m** Plasma total cholesterol (TC), triglyceride (TG), high-density lipoprotein cholesterol (HDL-C), and low-density lipoprotein cholesterol (LDL-C) concentrations in the mice (*n* = 6 per group). One-way ANOVA and Dunn post hoc test were used for analysis. **n** Plasma insulin concentration in the mice (*n* = 5 per group). Student’s *t* test was used for analysis. **o** Results of glucose tolerance test (GTT) (left)were quantified as area under the curve (AUC) (right) for the mice (*n* = 6 per group). One-way ANOVA and Dunn post hoc test were used for analysis. **p** Results of insulin tolerance test (ITT) (left) were quantified as AUC(right) for the mice (*n* = 6 per group). One-way ANOVA and Dunn post hoc test were used for analysis

To determine the role of ANXA1 specific to adipose tissue, adipose-specific *Anxa1* KO (*Anxa1*^*AKO*^) mice were generated by crossing adiponectin-Cre (*Adipoq*-Cre) and *Anxa1* flox/flox mice. In 8-week-old *Anxa1*^*AKO*^ mice, the protein and mRNA levels of ANXA1 were decreased in the SAT and VAT, while, which were no significant changes in the heart and liver (Supplementary Fig. 2a, b). To avoid the influence of ADIPOQ, the protein and mRNA levels of ADIPOQ in the SAT and VAT of 4-week-old *Anxa1*^*AKO*^ and the control group mice were measured (Supplementary Fig. 2c, d). There were no changes in the protein and mRNA levels of ADIPOQ in the two groups of mice, indicating that the subsequent changes in the obese phenotype of the mice were due to ANXA1 rather than ADIPOQ. After 16 weeks of HFD feeding (Supplementary Fig. 2e), the body weight of *Anxa1*^*AKO*^ mice increased significantly compared with that of their control littermates (Fig. 2a, b). Additionally, VO_2_, VCO_2_, and EE were decreased significantly in HFD-*Anxa1*^*AKO*^ mice compared to those in their control littermates (Fig. 2c–e), indicating a decline in the overall metabolic rate. Both SAT and VAT depots in *Anxa1*^*AKO*^ mice were increased significantly (Fig. 2f, g), resulting in an elevated fat mass/lean mass ratio (Fig. 2h). Furthermore, histological analysis of the SAT in HFD-*Anxa1*^*AKO*^ mice revealed that enlarged adipocytes dispersed in the SAT (Fig. 2i, j). However, the liver size and hepatic fat accumulation did not differ between HFD-*Anxa1*^*AKO*^ mice and their control littermates (Supplementary Fig. 2f and Fig. 2k, l).

Considering such defects in tissue morphology, we further investigated whether ANXA1 loss also affected adipose tissue regulatory functions. TC, TG, and LDL-C levels were upregulated significantly in HFD-*Anxa1*^*AKO*^ mice (Fig. 2m), suggesting that ANXA1 was required to maintain circulating lipid levels. Basal insulin levels were increased significantly in HFD-*Anxa1*^*AKO*^ mice (Fig. 2n). Compared with their control littermates, HFD-*Anxa1*^*AKO*^ mice exhibited glucose intolerance and insulin resistance (Fig. 2o, p), implying that ANXA1 was also essential for glucose regulation and for influencing insulin sensitivity in peripheral tissues. The data provide evidence for the indispensable role of ANXA1 in preserving the structural and functional integrity of adipose tissue.

### ANXA1 overexpression resists HFD-induced obesity in mice

**Fig. 3:**
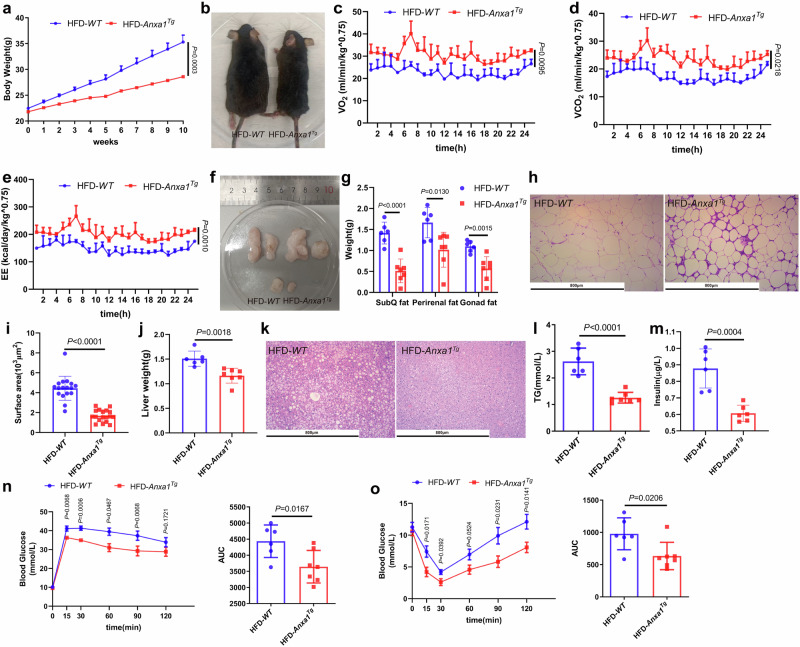
Overexpression of ANXA1 resists HFD-induced obesity in mice. **a**–**o** *WT* and *Anxa1* transgenic (*Anxa1*^*Tg*^) mice were fed with HFD for 10 weeks. **a** Average body weight ± s.e.m. of the mice (*n* = 6–7 per group). One-way ANOVA and Dunn post hoc test were used for analysis. **b** Representative photographs of the mice (*n* = 6–7 per group). **c**–**e** Oxygen consumption, carbon dioxide production, and energy expenditure within 24 h of the mice (*n* = 3 per group). One-way ANOVA and Dunn post hoc test were used for analysis. **f** Representative photographs of subcutaneous fat (upper left), perirenal fat (upper right), and gonadal fat (lower) from the mice (*n* = 6–7 per group). **g** Subcutaneous fat, perirenal fat, and gonadal fat weight of the mice (*n* = 6–7 per group). One-way ANOVA and Dunn post hoc test were used for analysis. **h** Hematoxylin & eosin-stained subcutaneous fat sections from the mice (*n* = 6–7 per group). Scale bar: 800 µm. **i** Adipocyte size in subcutaneous fat from the mice (*n* = 17–18 per group). Student’s *t* test was used for analysis. **j** Liver weight of the mice (*n* = 6–7 per group). Student’s *t* test was used for analysis. **k** Hematoxylin & eosin-stained liver sections from the mice (*n* = 7 per group). Scale bar: 800 µm. **l** Plasma triglyceride (TG) concentration in the mice (*n* = 6–7 per group). Student’s *t* test was used for analysis. **m** Plasma insulin concentration in the mice (*n* = 6 per group). Student’s *t* test was used for analysis. **n** Results of glucose tolerance test (GTT)(left) were quantified as area under the curve (AUC)(right) for the mice (*n* = 6–7 per group). One-way ANOVA and Dunn post hoc test were used for analysis. **o** Results of insulin tolerance test (ITT)(left) were quantified as AUC (right) for the mice (*n* = 6–7 per group). One-way ANOVA and Dunn post hoc test were used for analysis

To further elucidate the association between the increased adipose tissue ANXA1 levels and obesity, we examined metabolic function in a systemic ANXA1 overexpression mouse model (*Anxa1*^*Tg*^) (Supplementary Fig. 3a, b), generated by targeted transgenesis. Besides, we determined that there were no statistical changes in the protein and mRNA levels of ADIPOQ in SAT and VAT in 4-week-old mice to rule out the effect of ADIPOQ (Supplementary Fig. 3c, d), suggesting that the subsequent changes in the obese phenotype of mice were due to ANXA1 rather than ADIPOQ. Both *WT* and *Anxa1*^*Tg*^ mice were fed with HFD for 10 weeks, starting at 8 weeks of age (Supplementary Fig. 3e). *Anxa1*^*Tg*^ mice exhibited distinct resistance to HFD-induced obesity and weight gain compared with their littermate controls (Fig. 3a, b). In parallel, VO_2_, VCO_2_, and EE were increased significantly in HFD-*Anxa1*^*Tg*^ mice compared to those in their control littermates (Fig. 3c–e), indicating an enhancement of the overall metabolic rate. Notably, SAT and VAT depots in HFD-*Anxa1*^*Tg*^ mice were reduced significantly in size and weight (Fig. 3f, g), and the adipocytes of HFD-*Anxa1*^*Tg*^ mice were smaller than those in the control mice (Fig. 3h, i). HFD-*Anxa1*^*Tg*^ mice exhibited decreased liver weight gain (Fig. 3j). The livers of HFD-*WT* mice displayed a characteristic steatotic pale color, which was less apparent in the HFD-*Anxa1*^*Tg*^ livers (Supplementary Fig. 3f). HFD-*Anxa1*^*Tg*^ mice showed less liver lipid accumulation (Fig. 3k) and TG levels (Fig. 3l) compared with those in their littermate controls, suggesting reduced hepatic fat accumulation. Importantly, glucose tolerance and insulin sensitivity under HFD treatment were improved in *Anxa1*^*Tg*^ mice compared with those in their littermate controls (Fig. 3m–o). Taken together, the data implicate that ANXA1 exerts a beneficial effect on adipose tissue homeostasis, thereby mitigating lipotoxic insults in other organs.

### ANXA1 inhibits differentiation of adipose tissue-derived SVFs into mature adipocytes in mice

**Fig. 4:**
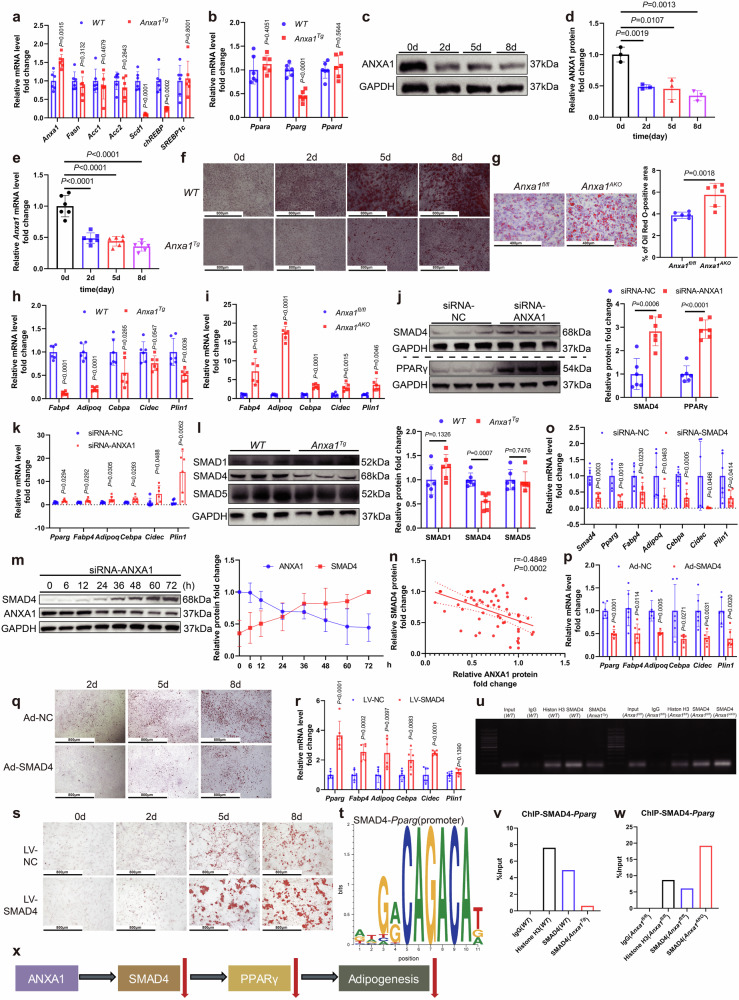
ANXA1 deficiency induces increased SMAD4 and PPARγ protein levels and promotes adipogenesis. **a** mRNA abundance of *Anxa1* and genes closely related to lipogenesis in SVFs from *WT* and *Anxa1*^*Tg*^ mice (*n* = 6 per group). One-way ANOVA and Dunn post hoc test were used for analysis. **b** mRNA abundance of *Ppara*, *Pparg* and *Ppard* in SVFs from *WT* and *Anxa1*^*Tg*^ mice (*n* = 6 per group). One-way ANOVA and Dunn post hoc test were used for analysis. **c** Representative western blot of ANXA1 at different time points after adipogenic induction during the adipogenesis of SVFs (*n* = 3 per group). **d** Fold change of ANXA1 protein levels, quantified from **c** (*n* = 3 per group). Student’s *t* test was used for analysis. **e** mRNA levels of *Anxa1* at different time points after adipogenic induction during the adipogenesis of SVFs (*n* = 6 per group). Student’s *t* test was used for analysis. **f** Oil Red O staining of SVFs at day 0, day 2, day 5 and day 8 after adipogenic induction from *WT* and *Anxa1*^*Tg*^ mice (*n* = 6 per group). Scale bar: 800 µm. **g** Oil Red O staining (left) was quantified as Oil Red O-positive area (right) in SVFs induced to differentiate on day 2 from *Anxa1*^*fl/fl*^ and *Anxa1*^*AKO*^ mice (*n* = 6 per group). Student’s *t* test was used for analysis. Scale bar: 400 µm. **h** mRNA abundance of genes closely related to adipogenesis in SVFs from *WT* and *Anxa1*^*Tg*^ mice (*n* = 6 per group). One-way ANOVA and Dunn post hoc test were used for analysis. **i** mRNA abundance of genes closely related to adipogenesis in SVFs from *Anxa1*^*fl/fl*^ and *Anxa1*^*AKO*^ mice (*n* = 6 per group). One-way ANOVA and Dunn post hoc test were used for analysis. **j** Representative western blotting and quantification of SMAD4 and PPARγ from SVFs transfected with ANXA1 siRNA or negative control (NC) siRNA for 48 h (*n* = 6 per group). One-way ANOVA and Dunn post hoc test were used for analysis. **k** mRNA abundance of *Pparg* and genes closely related to adipogenesis in SVFs transfected with ANXA1 siRNA or NC siRNA for 48 h (*n* = 6 per group). One-way ANOVA and Dunn post hoc test were used for analysis. **l** Representative western blotting and quantification of SMAD1, SMAD4, and SMAD5 in SVFs from *WT* and *Anxa1*^*Tg*^ mice (*n* = 6 per group). One-way ANOVA and Dunn post hoc test were used for analysis. **m** Representative western blotting and quantification of ANXA1 and SMAD4 from SVFs at different time points after transfection with ANXA1 siRNA and ANXA1 siRNA(1) (*n* = 3–4 per group). **n** Spearman correlation between fold change of ANXA1 and SMAD4 protein levels (*n* = 3–4 per group). **o** mRNA abundance of *Smad4*, *Pparg* and genes closely related to adipogenesis in SVFs transfected with SMAD4 siRNA or NC siRNA for 48 h (*n* = 6 per group). One-way ANOVA and Dunn post hoc test were used for analysis. **p** mRNA abundance of *Pparg* and genes closely related to adipogenesis in SVFs transfected with SMAD4 adenovirus (Silencing SMAD4) or NC adenovirus for 72 h (*n* = 6 per group). One-way ANOVA and Dunn post hoc test were used for analysis. **q** After transfected with SMAD4 adenovirus or NC adenovirus for 72 h, Oil Red O staining of SVFs at different time points after adipogenic induction (*n* = 6 per group). Scale bar: 800 µm. **r** mRNA abundance of *Pparg* and genes closely related to adipogenesis in SVFs transfected with SMAD4 lentivirus (Overexpressing SMAD4) or NC lentivirus for 96 h (*n* = 6 per group). One-way ANOVA and Dunn post hoc test were used for analysis. **s** After transfected with SMAD4 lentivirus or NC lentivirus for 96 h, Oil Red O staining of SVFs at different time points after adipogenic induction (*n* = 6 per group). Scale bar: 800 µm. **t** Predicted SMAD4 binding *Pparg* promoter motifs by UCSC Genome Browser Home and PROMO. **u** The binding of *Pparg* promoter motifs and IgG, Histone H3 and SMAD4 was further explored by ChIP-PCR assays in SVFs from *WT*, *Anxa1*^*Tg*^, *Anxa1*^*fl/fl*^ and *Anxa1*^*AKO*^. **v**–**w** The binding of *Pparg* promoter region and IgG, Histone H3 and SMAD4 was further explored by ChIP-qPCR assays in SVFs from *WT*, *Anxa1*^*Tg*^, *Anxa1*^*fl/fl*^ and *Anxa1*^*AKO*^. **x** Schematic diagram of the ANXA1-SMAD4-PPARγ-Adipogenesis axis

To investigate the molecular mechanism underlying the impact of ANXA1 on adipose tissue, SVFs, a heterogeneous cell population that mainly contains a large number of preadipocytes, were extracted from adipose tissues (Supplementary Fig. 4a, b). We analyzed the mRNA levels of several genes closely associated with lipogenesis, including fatty acid synthase (*Fasn*), acetyl-CoA carboxylase 1 (*Acc1*), acetyl-CoA carboxylase 2 (*Acc2*), stearoyl-CoA desaturase (*Scd1*), responsive element binding protein (*ChREBP*), and sterol-regulatory element binding protein-1c (*SREBP1c*) in SVFs from *Anxa1*^*Tg*^ and *Anxa1*^*AKO*^ mice. Compared with those in the control group, the mRNA levels of *Scd1* and *ChREBP* were reduced significantly in the *Anxa1*^*Tg*^ group (Fig. 4a), whereas they were increased significantly in the *Anxa1*^*AKO*^ group (Supplementary Fig. 4c). *Scd1* is reduced notably during the differentiation process of 3T3-L1 cells, and both *ChREBP* and *Scd1* are closely related to adipogenesis.^35^ Additionally, *Pparg* mRNA levels decreased significantly in the SVFs of *Anxa1*^*Tg*^ mice, and increased significantly in the SVFs of *Anxa1*^*AKO*^ mice (Fig. 4b, Supplementary Fig. 4d). However, no significant changes of peroxisome proliferator-activated receptor alpha (*Ppara*) and peroxisome proliferator-activated receptor delta (*Ppard*) mRNA levels were observed (Fig. 4b, Supplementary Fig. 4d), proving that the changes in *Pparg* in SVFs caused by ANXA1 were specific. The protein and mRNA levels of PPARγ were elevated during SVFs differentiation (Supplementary Fig. 5a). PPARγ is a key regulator of adipogenesis (Supplementary Fig. 5b).^36^ Treatment with the PPARγ antagonist 2-chloro-5-nitrobenzanilide (GW9662) inhibited the expression of *Pparg*, fatty acid-binding protein 4 (*Fabp4*), *Adipoq*, CCAAT enhancer-binding protein alpha (*Cebpa*), cell death-inducing DFFA-like effector c (*Cidec*), and perilipin 1 (*Plin1*) (key genes in adipogenesis) in SVFs (Supplementary Fig. 5c). Rosiglitazone, a PPARγ agonist, activated the expression of *Pparg*, *Fabp4*, *Adipoq*, *Cebpa*, *Cidec*, and *Plin1* in SVFs (Supplementary Fig. 5d). GW9662 inhibited SVFs differentiation into mature adipocytes, whereas rosiglitazone promoted the process (Supplementary Fig. 5e).

We observed that the protein and mRNA levels of ANXA1 decreased during SVFs differentiation (Fig. 4c–e), so we conjectured that the cells had a higher degree of maturation as ANXA1 was reduced. Moreover, we found that over 98% of SVFs expressed both CD105 (cell surface marker of preadipocytes) and ANXA1, based on flow cytometry and fluorescence localization staining (Supplementary Fig. 6a, b), indicating that the extracted SVFs were mostly preadipocytes with high expression of ANXA1 and no other cells. This further demonstrated the important role of ANXA1 in preadipocytes. We corroborated that the induced differentiation potential of SVFs from *Anxa1*^*Tg*^ mice was diminished significantly (Fig. 4f), whereas SVFs from *Anxa1*^*AKO*^ mice exhibited a pronounced propensity for adipocyte maturation (Fig. 4g). In comparison to the control group, there was a significant decrease in the mRNA levels of adipogenesis-related genes in SVFs of *Anxa1*^*Tg*^ mice (Fig. 4h) and a significant increase in SVFs of *Anxa1*^*AKO*^ mice (Fig. 4i). Next, we employed specific siRNA to silence ANXA1 in SVFs from *WT* mice, achieving an approximately 70% reduction in ANXA1 protein levels and a 90% reduction in *Anxa1* mRNA levels (Supplementary Fig. 6c, d). ANXA1 knockdown significantly increased the PPARγ protein levels (Fig. 4j, Supplementary Fig. 6d), and the mRNA levels of *Pparg*, *Fabp4*, *Adipoq*, *Cebpa*, *Cidec*, and *Plin1* (Fig. 4k, Supplementary Fig. 6e). In addition, the silencing ANXA1 significantly increased the mRNA levels of inflammation-related factors, such as interleukin 6 (*IL-6*), C-C motif chemokine ligand 2 (*Ccl2*), and C-X-C motif chemokine ligand 10 (*Cxcl10*) (Supplementary Fig. 6f).

Multiple studies have shown that SMAD family proteins can induce adipogenesis through PPARγ.^37^ SMAD4 protein levels were reduced significantly, whereas SMAD1 and SMAD5 protein levels remained unchanged in SVFs from *Anxa1*^*Tg*^ mice (Fig. 4l). Silencing ANXA1 using siRNA upregulated SMAD4 levels (Fig. 4j, Supplementary Fig. 6d). We identified that SMAD4 protein levels were negatively correlated with ANXA1 protein levels (Fig. 4m, n, Supplementary Fig. 6g). Compared with that in NCD-*WT* mice, ANXA1 protein levels decreased significantly in SVFs from *WT* mice fed with HFD for 12 weeks; whereas, SMAD4 protein levels increased significantly (Supplementary Fig. 6h). Additionally, the average expression levels of *ANXA1* mRNA in adipose stem and progenitor cells within the SAT were significantly lower among individuals with a BMI > 40 relative to those with a BMI < 30, as observed in the human single-cell atlas data by Emont et al. (Supplementary Fig. 6i)^38^.

Silencing SMAD4 using siRNA in SVFs significantly attenuated the protein levels of PPARγ (Supplementary Fig. 6j, k), and the mRNA levels of *Pparg*, *Fabp4*, *Adipoq*, *Cebpa*, *Cidec*, and *Plin1* (Fig. 4o, Supplementary Fig. 6l). SMAD4 adenovirus was also used to silence SMAD4 in SVFs (Supplementary Fig. 6m), and the mRNA levels of these genes also decreased significantly (Fig. 4p). Meanwhile, SVFs were less induced into mature adipocytes (Fig. 4q). SMAD4 lentivirus was used to overexpress SMAD4 in SVFs (Supplementary Fig. 6n), which increased significantly the mRNA levels of the above genes (Fig. 4r), and SVFs were induced to a greater extent into mature adipocytes (Fig. 4s). We predicted that SMAD4 was a transcription factor of *Pparg* (Fig. 4t). The results suggested that possibly ANXA1 reduced the entry of SMAD4 into the nucleus, thereby reducing the initiate of *Pparg* transcription. Therefore, we used ChIP-PCR and ChIP-qPCR to detect the binding of SMAD4 to the *Pparg* promoter region in SVFs from *WT*, *Anxa1*^*Tg*^, *Anxa1*^*fl/fl*^, and *Anxa1*^*AKO*^ mice (Fig. 4u–w). The results indicated that high expression of ANXA1 was accompanied by a reduced binding of SMAD4 to the *Pparg* promoter region. Upon silencing of ANXA1, the binding of SMAD4 to the *Pparg* promoter region was enhanced.

In summary, ANXA1 reduces SMAD4 protein levels, which subsequently downregulates PPARγ and inhibits SVFs differentiation into mature adipocytes (Fig. 4x).

### ANXA1- PDLIM7 interaction promotes SMAD4 ubiquitination and inhibits adipogenesis in SVFs

**Fig. 5:**
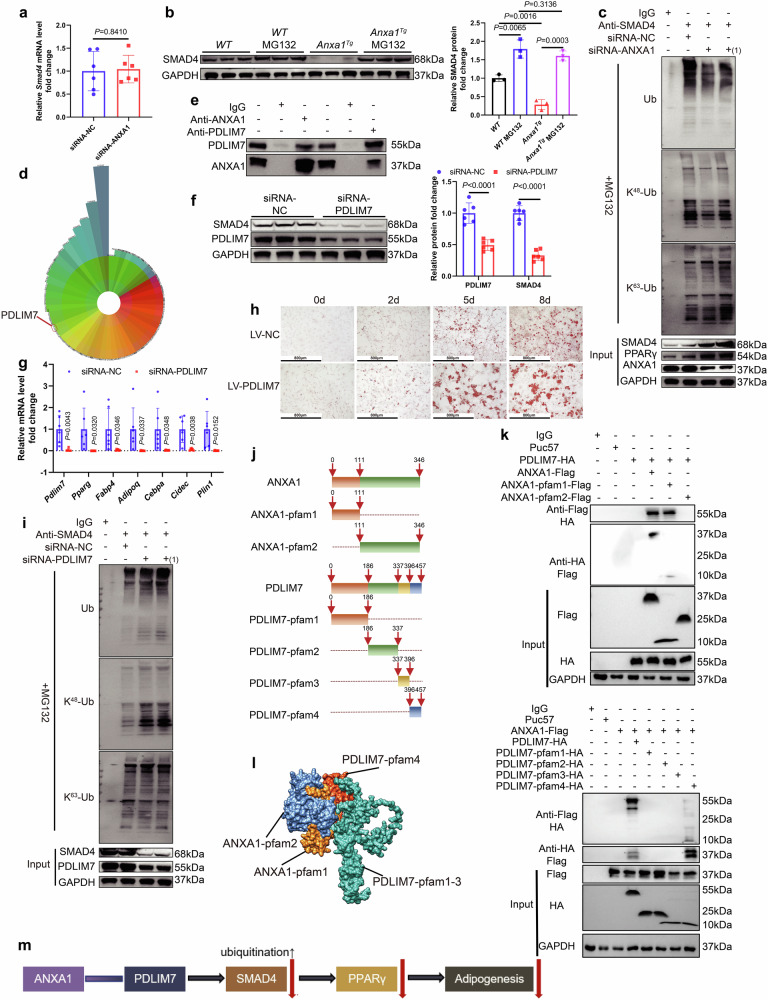
The interaction between ANXA1 and PDLIM7 increases the degradation of SMAD4 after ubiquitination and inhibits adipogenesis in SVFs. **a** mRNA abundance of *Smad4* in SVFs transfected with ANXA1 siRNA or NC siRNA for 48 h (*n* = 6 per group). Student’s *t* test was used for analysis. **b** Representative western blotting and quantification of SMAD4 from *WT*-SVFs, *Anxa1*^*Tg*^-SVFs, and *WT*-SVFs and *Anxa1*^*Tg*^-SVFs incubated with 10 µM MG132 for 6 h (*n* = 3 per group). Student’s *t* test was used for analysis. **c** SVFs were transfected with ANXA1 siRNA, ANXA1 siRNA(1) or NC siRNA for 48 h, followed by incubation with 10 µM MG132 for 6 h. Cell lysates were immunoprecipitated with an anti-SMAD4 antibody and then immunoblotted with an anti-ubiquitin (Ub) antibody, anti-K48-linkage specific polyubiquitin (K^48^-Ub) antibody and anti-K63-linkage specific polyubiquitin (K^63^-Ub) antibody. **d** The polar coordinate bar chart shows that PDLIM7 has a strong interaction with ANXA1 in the IP-MS results for ANXA1 and IgG. **e** Co-immunoprecipitation assay of ANXA1 and PDLIM7 in SVFs. **f** Representative western blotting and quantification of SMAD4 and PDLIM7 from SVFs transfected with PDLIM7 siRNA or NC siRNA for 48 h (*n* = 6 per group). One-way ANOVA and Dunn post hoc test were used for analysis. **g** mRNA abundance of *Pdlim7*, *Pparg* and genes closely related to adipogenesis in SVFs transfected with PDLIM7 siRNA or NC siRNA for 48 h (*n* = 6 per group). One-way ANOVA and Dunn post hoc test were used for analysis. **h** After incubation with PDLIM7 lentivirus (Overexpressing PDLIM7) or NC lentivirus for 96 h, Oil Red O staining of SVFs at different time points after adipogenic induction (*n* = 6 per group). Scale bar: 800 µm. **i** SVFs were transfected with PDLIM7 siRNA, PDLIM7 siRNA(1) or NC siRNA for 48 h, followed by incubation with 10 µM MG132 for 6 h. Cell lysates were immunoprecipitated with an anti-SMAD4 antibody and then immunoblotted with an anti-Ub antibody, anti-K^48^-Ub antibody and anti-K^63^-Ub antibody. **j** Schematic diagram of peptide segment design. **k** HEK 293T cells were transfected with different peptide segment vector plasmids or empty plasmids (Puc57) for 48 h. Cell lysates were immunoprecipitated with an anti-Flag tag antibody and then immunoblotted with an anti-HA tag antibody, or conversely, cell lysates were immunoprecipitated with an anti-HA tag antibody and then immunoblotted with an anti-Flag tag antibody. **l** Schematic representation of the interaction of ANXA1 with PDLIM7. ANXA1-pfam1 in yellow, ANXA1-pfam2 in blue, PDLIM7-pfam1-3 in green and PDLIM7-pfam4 in orange-red. **m** Schematic diagram of the ANXA1-PDLIM7-SMAD4-PPARγ-Adipogenesis axis

Notably, we observed that silencing ANXA1 did not affect *Smad4* mRNA levels (Fig. 5a, Supplementary Fig. 7a), raising the hypothesis that ANXA1 could promote SMAD4 protein degradation. The ubiquitin-proteasome pathway is responsible for the degradation of numerous proteins.^39^ In SVFs, treatment with the proteasome inhibitor MG132 increased SMAD4 protein levels (Supplementary Fig. 7b), suggesting that SMAD4 could be degraded via the ubiquitin-proteasome pathway. In the SVFs from *Anxa1*^*Tg*^ mice, SMAD4 protein levels were reduced significantly compared with that from *WT* mice, but after treatment with MG132, SMAD4 protein levels showed no difference between the two groups (Fig. 5b). In the SVFs from *Anxa1*^*AKO*^ mice, SMAD4 protein levels increased significantly compared with that in the control group mice. After treatment with MG132, there were no significant changes in SMAD4 protein levels in the SVFs from *Anxa1*^*AKO*^ mice (Supplementary Fig. 7c). Therefore, we hypothesized that ANXA1 affected the proteasomal degradation of SMAD4, possibly by interfering with its ubiquitination. Co-immunoprecipitation (co-IP) experiments were performed to measure SMAD4 ubiquitination levels when ANXA1 was silenced in SVFs, suggesting that ANXA1 promoted significantly SMAD4 ubiquitination in SVFs (Fig. 5c). To be more specific, ANXA1 promoted significantly the K48-linkage specific ubiquitination of SMAD4 in SVFs, but did not alter the K63-linkage specific ubiquitination (Fig. 5c).

Subsequently, using immunoprecipitation coupled with mass spectrometry (IP-MS), we identified 55 proteins that specifically interacted with ANXA1 within SVFs, among which PDLIM7 exhibited strong interactions with ANXA1 and was associated with SMAD4 protein (OMIX006798, Fig. 5d, Supplementary Fig. 7d, e). Co-IP and colocalization of immunofluorescence (co-IF) experiments showed that ANXA1 and PDLIM7 interacted with each other (Fig. 5e, Supplementary Fig. 7f). When PDLIM7 was silenced in SVFs using PDLIM7 siRNA, SMAD4 and PPARγ protein levels decreased significantly, whereas *Anxa1* and *Smad4* mRNA levels remained unchanged (Fig. 5f, Supplementary Fig. 7g–j). PDLIM7 siRNA treatment decreased significantly the mRNA levels of *Pdlim7*, *Pparg*, *Fabp4*, *Adipoq*, *Cebpa*, *Cidec*, and *Plin1* (Fig. 5g, Supplementary Fig. 7j). Overexpression of PDLIM7 in SVFs using PDLIM7 lentivirus resulted in a significant increase in SMAD4 protein levels and the mRNA levels of the aforementioned adipogenesis-related genes (Supplementary Fig. 7k, l), leading to a higher induction into mature adipocytes from SVFs (Fig. 5h). Co-IP experiments showed that SMAD4 total ubiquitination was upregulated significantly, K48-linkage specific ubiquitination was increased significantly, whereas K63-linkage specific ubiquitination was not altered significantly, when PDLIM7 was silenced in SVFs (Fig. 5i). The findings suggested that PDLIM7 inhibited SMAD4 K48-linkage specific ubiquitination and upregulated SMAD4 protein levels in SVFs.

Furthermore, we used two ANXA1 peptides and four PDLIM7 peptides encompassing distinct protein domains to validate the interface of their interactions (Fig. 5j). Co-IP experiments demonstrated that ANXA1-pfam1 (protein fragment 1) and PDLIM7-pfam4(protein fragment 4) interacted with each other (Fig. 5k, l). Collectively, the findings suggest that ANXA1 promotes SMAD4 K48-linkage specific ubiquitination by binding to PDLIM7, resulting in a decrease in SMAD4 protein levels and inhibition of SVFs differentiation into mature adipocytes (Fig. 5m).

### PDLIM7 inhibits MYCBP2-mediated SMAD4 ubiquitination in SVFs

**Fig. 6:**
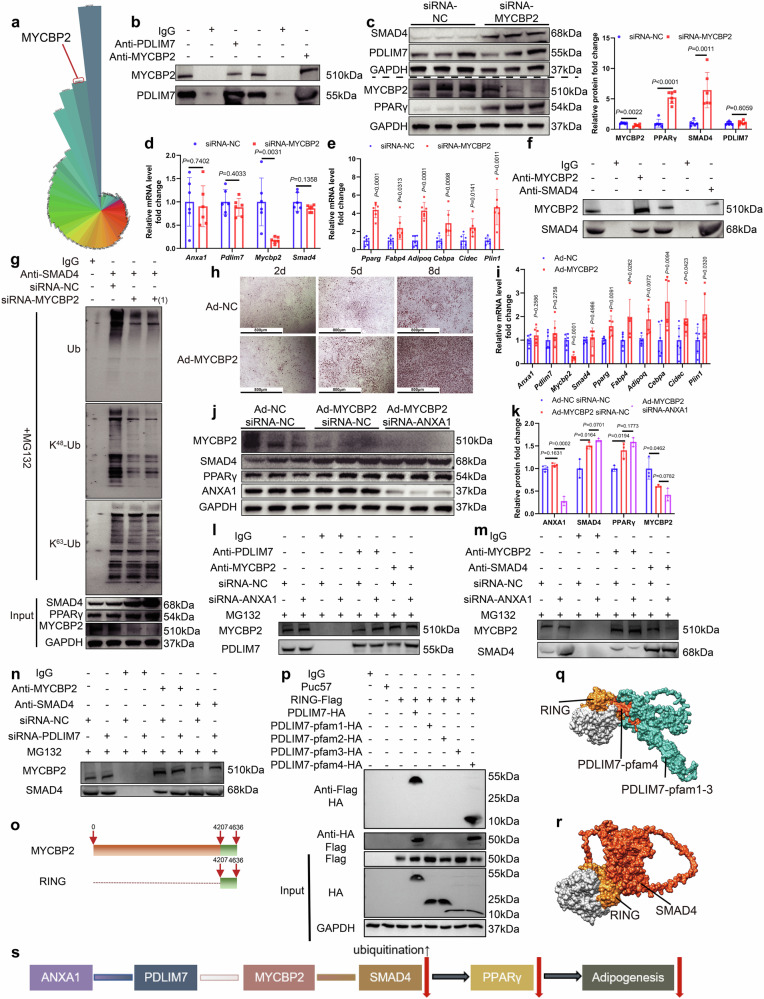
The interaction between PDLIM7 and MYCBP2 inhibits MYCBP2-mediated ubiquitination of SMAD4 and promotes adipogenesis in SVFs. **a** The polar coordinate bar chart shows that MYCBP2 has a strong interaction with PDLIM7 in the IP-MS results for PDLIM7 and IgG. **b** Co-immunoprecipitation assay of PDLIM7 and MYCBP2 in SVFs. **c** Representative western blotting and quantification of MYCBP2, PPARγ, SMAD4 and PDLIM7 from SVFs transfected with MYCBP2 siRNA or NC siRNA for 48 hs (*n* = 6 per group). One-way ANOVA and Dunn post hoc test were used for analysis. **d** mRNA abundance of *Anxa1*, *Pdlim7*, *Mycbp2* and *Smad4* in SVFs transfected with MYCBP2 siRNA or NC siRNA for 48 h (*n* = 6 per group). One-way ANOVA and Dunn post hoc test were used for analysis. **e** mRNA abundance of *Pparg* and genes closely related to adipogenesis in SVFs transfected with MYCBP2 siRNA or NC siRNA for 48 h (*n* = 6 per group). One-way ANOVA and Dunn post hoc test were used for analysis. **f** Co-immunoprecipitation assay of MYCBP2 and SMAD4 in SVFs. **g** SVFs were transfected with MYCBP2 siRNA, MYCBP2 siRNA(1) or NC siRNA for 48 h, followed by incubation with 10 µM MG132 for 6 h. Cell lysates were immunoprecipitated with an anti-SMAD4 antibody and then immunoblotted with an anti-Ub antibody, anti-K^48^-Ub antibody and anti-K^63^-Ub antibody. **h** After transfected with MYCBP2 adenovirus (Silencing MYCBP2) or NC adenovirus for 72 h, Oil Red O staining of SVFs at different time points after adipogenic induction (*n* = 6 per group). Scale bar: 800 µm. **i** mRNA abundance of *Anxa1*, *Pdlim7*, *Mycbp2*, *Smad4*, *Pparg* and genes closely related to adipogenesis in SVFs transfected with MYCBP2 adenovirus or NC adenovirus for 72 h (*n* = 6 per group). One-way ANOVA and Dunn post hoc test were used for analysis. Representative western blotting (**j**) and quantification (**k**) of MYCBP2, SMAD4, PPARγ and ANXA1 from SVFs transfected with MYCBP2 adenovirus or NC adenovirus for 96 h and transfected with ANXA1 siRNA or NC siRNA for 48 h (*n* = 3 per group). One-way ANOVA and Dunn post hoc test were used for analysis. **l** Co-immunoprecipitation assay of MYCBP2 and PDLIM7 in SVFs transfected with ANXA1 siRNA or NC siRNA for 48 h followed by incubation with 10 µM MG132 for 6 h. **m** Co-immunoprecipitation assay of MYCBP2 and SMAD4 in SVFs transfected with ANXA1 siRNA or NC siRNA for 48 h followed by incubation with 10 µM MG132 for 6 h. **n** Co-immunoprecipitation assay of MYCBP2 and SMAD4 in SVFs transfected with PDLIM7 siRNA or NC siRNA for 48 h followed by incubation with 10 µM MG132 for 6 h. **o** Schematic diagram of peptide segment design. **p** HEK 293T cells were transfected with different peptide segment vector plasmids or empty plasmids for 48 h. Cell lysates were immunoprecipitated with an anti-Flag tag antibody and then immunoblotted with an anti-HA tag antibody, or conversely, cell lysates were immunoprecipitated with an anti-HA tag antibody and then immunoblotted with an anti-Flag tag antibody. **q** Schematic representation of the interaction of PDLIM7 with MYCBP2. PDLIM7-pfam1-3 in green, PDLIM7-pfam4 in orange-red, RING in yellow and MYCBP2 in gray except for RING. **r** Schematic representation of the interaction of MYCBP2 with SMAD4. RING in yellow, MYCBP2 in gray except for RING and SMAD4 in orange-red. **s** Schematic diagram of the ANXA1-PDLIM7-MYCBP2-SMAD4-PPARγ-Adipogenesis axis

To investigate whether PDLIM7 directly interacted with SMAD4, we employed IP-MS to identify proteins interacting with PDLIM7 (OMIX006798, Fig. 6a, Supplementary Fig. 8a). Notably, SMAD4 was not detected among the proteins that interacted with PDLIM7. However, MYCBP2, an atypical E3 ubiquitin-protein ligase known to specifically mediate ubiquitination of threonine and serine residues on target proteins, exhibited a strong interaction with PDLIM7. The interaction was further confirmed by co-IP and co-IF experiments (Fig. 6b, Supplementary Fig. 8b).

Given that SMAD4 can be polyubiquitinated and degraded by SMAD specific E3 ubiquitin protein ligase (SMURF),^40^ tripartite motif 47 (TRIM47),^41^ and beta-transducin repeat containing E3 ubiquitin protein ligase (β-TRCP)^42^ by both K48- and K63-linked ubiquitination, MYCBP2 might also ubiquitinate SMAD4 in the similar way and promote its degradation. Upon silencing MYCBP2 in SVFs using MYCBP2 siRNA, we observed a significant increase in SMAD4 and PPARγ protein levels (Fig. 6c, Supplementary Fig. 8c). However, no significant changes in the PDLIM7 protein levels and the mRNA levels of *Anxa1*, *Pdlim7*, and *Smad4* were observed (Fig. 6c, d and Supplementary Fig. 8c, d). Additionally, the mRNA levels of *Pparg*, *Fabp4*, *Adipoq*, *Cebpa*, *Cidec*, and *Plin1* increased significantly following MYCBP2 siRNA treatment (Fig. 6e, Supplementary Fig. 8e). Subsequently, we confirmed the interaction between MYCBP2 and SMAD4 by co-IP and co-IF experiments (Fig. 6f, Supplementary Fig. 8f). Notably, when MYCBP2 was silenced in SVFs, SMAD4 total ubiquitination and the K48-linkage specific ubiquitination were downregulated significantly, whereas there was no significant change on K63-linkage specific ubiquitination (Fig. 6g). These data indicated that MYCBP2 ubiquitinated the SMAD4 at K48. When MYCBP2 adenovirus was used to silence MYCBP2 in SVFs, SVFs were induced to a greater extent into mature adipocytes (Fig. 6h), as well as the protein levels of SMAD4 and PPARγ and the mRNA levels of the above adipogenesis-related genes were increased significantly (Supplementary Fig. 8g, Fig. 6i). Based on these findings, we posited that PDLIM7 inhibited SMAD4 ubiquitination by competitively interacting with MYCBP2. Moreover, when siRNA-ANXA1 was used to silence ANXA1 in the MYCBP2-KO-SVFs after using Ad-MYCBP2, there were no significant changes in the protein levels of SMAD4 and PPARγ (Fig. 6j, k and Supplementary Fig. 8h), and the mRNA levels of these adipogenesis-related genes also did not exhibit significant changes (Supplementary Fig. 8i). The results suggested that MYCBP2 did indeed play a role downstream of the ANXA1 axis.

We further hypothesized that the interactions among ANXA1, PDLIM7, and MYCBP2 were competitive. Co-IP experiments showed that silencing ANXA1 in SVFs using ANXA1 siRNA enhanced significantly the interaction between PDLIM7 and MYCBP2 (Fig. 6l), whereas the interaction between MYCBP2 and SMAD4 was weakened significantly (Fig. 6m). Conversely, the interaction between MYCBP2 and SMAD4 was enhanced significantly by PDLIM7 siRNA treatment (Fig. 6n). The data suggested that when ANXA1 was expressed highly, it was bound competitively to PDLIM7 more strongly than MYCBP2, exposing the binding site of MYCBP2 to SMAD4 and promoting the ubiquitination of SMAD4. The MYCBP2 E3 machinery is presumed to utilize a RING domain to bind to the E2 ubiquitin-conjugating enzyme.^43^ Consequently, we constructed the RING-FLAG peptide, a recombinant C-terminal version of MYCBP2 encompassing the RING domain and a FLAG label (Fig. 6o). Co-IP experiments demonstrated that RING-FLAG and PDLIM7-pfam4 interacted with each other (Fig. 6p, q), which interfered with MYCBP2-mediated ubiquitination of SMAD4 (Fig. 6r).

In mature adipocytes, we also discovered the presence of ANXA1-PDLIM7-MYCBP2-SMAD4 interactions by co-IP experiments (Supplementary Fig. 9a–c). To further investigate whether the molecular mechanism existed in other cells, we detected the interactions between the above proteins in murine microvascular endothelial cell line (H5V) by co-IP experiments, and found that ANXA1-PDLIM7-MYCBP2-SMAD4 interactions also existed (Supplementary Fig. 9d–f). Subsequently, we used siRNA to silence ANXA1 in H5V cells and found that the protein levels of SMAD4 and PPARγ still increased significantly (Supplementary Fig. 9g). The data suggested that the above molecular mechanisms also existed in mature adipocytes and the H5V cell line.

Collectively, the findings demonstrate that PDLIM7 inhibits MYCBP2-mediated SMAD4 K48-linkage specific ubiquitination through its interaction with MYCBP2 (Fig. 6s).

### Ac2-26 interacts with PDLIM7 to inhibit adipogenesis and prevents obesity in HFD mice

**Fig. 7:**
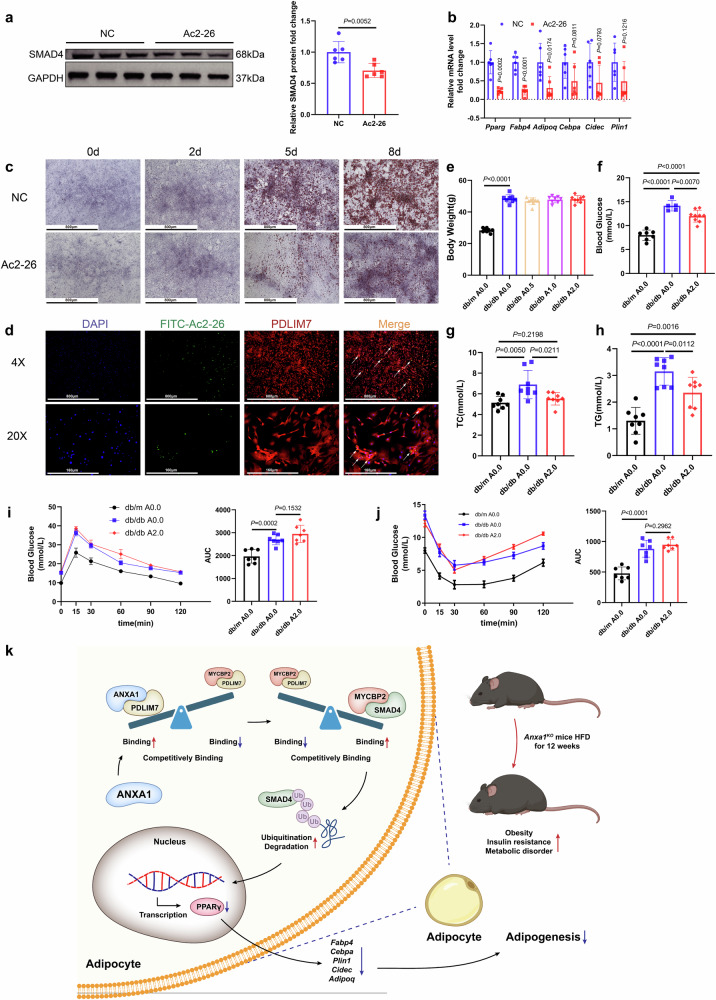
Ac2-26 inhibits adipogenesis and prevents obesity in HFD mice. **a** Representative western blotting and quantification of SMAD4 from SVFs incubated with 0.1 mg/ml Ac2-26 or DMSO (NC) for 48 h (*n* = 6 per group). Student’s *t* test was used for analysis. **b** mRNA abundance of *Pparg* and genes closely related to adipogenesis in SVFs incubated with 0.1 mg/ml Ac2-26 or DMSO for 48 h (*n* = 6 per group). One-way ANOVA and Dunn post hoc test were used for analysis. **c** Oil Red O staining of SVFs at different time points after adipogenic induction incubated with 0.1 mg/ml Ac2-26 or DMSO (*n* = 6 per group). Scale bar: 800 µm. **d** ICC experiments demonstrate the interaction between PDLIM7 and FITC- Ac2-26. Scale bar: 800 µm, 160 µm. **e**–**j** Eight-week-old db/m mice, db/db mice, and db/db mice were injected intraperitoneally with 0, 0.5, 1.0, or 2.0 mg/kg of Ac2-26 (dissolved in PBS) for 10 weeks and were fed with HFD throughout. **e** Body weight of the five groups of the mice (*n* = 8 per group). Student’s *t* test was used for analysis. **f**–**h** Blood glucose, total cholesterol (TC), and triglyceride (TG) concentrations in db/m mice, db/db mice, and db/db mice injected intraperitoneally with 0.0 or 2.0 mg/kg of Ac2-26 (*n* = 5–9 per group). Student’s *t* test was used for analysis. **i** Results of glucose tolerance test (GTT)(left) were quantified as area under the curve (AUC)(right) for db/m mice, db/db mice, and db/db mice injected intraperitoneally with 0.0 or 2.0 mg/kg of Ac2-26 (*n* = 7 per group). One-way ANOVA and Dunn post hoc test were used for analysis. **j** Results of insulin tolerance test (ITT)(left) were quantified as AUC(right) for the three groups of mice (*n* = 7 per group). One-way ANOVA and Dunn post hoc test were used for analysis. **k** Schematic created with BioRender.com to better illustrate the conceptual advancement in our understanding of how ANXA1 affects adipogenesis. Overexpression of ANXA1 in SVFs enhanced its interaction with PDLIM7, thereby weakening the interaction of PDLIM7 with MYCBP2.This exposed the MYCBP2-binding site, allowing it to bind more readily to SMAD4 and mediate its ubiquitination and degradation. SMAD4 degradation downregulated PPARγ transcription and reduced adipogenesis. Conversely, knocking out ANXA1 made HFD-mice more obese

To further explore whether Ac2-26, an active N-terminal peptide of ANXA1, could prevent obesity by inhibiting adipogenesis, Ac2-26 was incubated with SVFs at 0.1 mg/mL. SMAD4 protein levels in SVFs were reduced significantly after Ac2-26 treatment (Fig. 7a), and the mRNA levels of key genes in adipogenesis decreased significantly, compared to that in the control group (Fig. 7b). In addition, the induced differentiation potential of SVFs treated with Ac2-26 was reduced significantly (Fig. 7c). We constructed FITC- Ac2-26, which was incubated with SVFs at 0.1 mg/mL for 48 h, followed by immunocytochemistry. The results strongly suggested that FITC- Ac2-26 could interact with PDLIM7 (Fig. 7d). The above results indicated that Ac2-26 could inhibit adipogenesis via the similar mechanism to ANXA1.

Diet-induced obesity on a HFD is likely controlled by a variety of genetic factors, while db/db mice, as recognized leptin receptor gene-deficient mice recessive mutations on chromosome 4, were used to establish a diabetic-obese mouse model and to investigate whether exogenous ANXA1 (Ac2-26) has a therapeutic effect on obesity. Ac2-26 was intraperitoneally injected into 8-week-old db/db mice fed with HFD every other day at doses of 0, 0.5, 1.0, and 2 mg/kg for 10 weeks. Compared with the mice receiving 0 mg/kg Ac2-26, the body weight of the other three groups did not change significantly (Fig. 7e). The blood glucose, TC, and TG levels in db/db mice treated with Ac2-26 (2 mg/kg) were significantly lower than the levels observed in db/db mice without Ac2-26 treatment (Fig. 7f–h). However, no significant changes in glucose or insulin tolerance were observed (Fig. 7i, j). Therefore, we speculate that Ac2-26 treatment to prevent obesity in db/db mice mainly targets adipose tissue and may not be directly related to pancreatic islet function.

To investigate whether ANXA1 affected mice obesity via PPARγ in vivo, we treated HFD-fed *Anxa1*^*AKO*^ mice with GW9662 (intraperitoneal injection every other day at a dose of 1 mg/kg for 16 weeks). Compared with those without GW9662 treatment, the body (Supplementary Fig. 10a, b), liver (Supplementary Fig. 10c, d), and SAT weights (Supplementary Fig. 10e, f) of HFD-*Anxa1*^*AKO*^ mice treated with GW9662 were reduced significantly. Simultaneously, the lipid accumulation was decreased in HFD-*Anxa1*^*AKO*^ mice treated with GW9662 (Supplementary Fig. 10g), and the glucose and insulin tolerance improved significantly (Supplementary Fig. 10h–j). The data suggest that GW9662 prevents obesity induced by Adipose tissue-specific ANXA1 KO.

## Discussion

The global prevalence of obesity has made it imperative to identify targets for obesity treatment. This study sheds light on important quandaries in the field. Firstly, our study reveals for the first time the pathway via which ANXA1 prevents obesity by inhibiting adipogenesis in mice. Transcriptional analyses by Pietiläinen et al.^32^ and van der Kolk et al.^33^ show that *ANXA1* mRNA levels are significantly higher in twins with higher BMI than in those with lower BMI. We validate these results and find that ANXA1 protein and mRNA levels are upregulated significantly in the human and mouse SAT under obese conditions. Global knockout of ANXA1 followed by an HFD in C57BL/6 mice aggravates obesity and insulin resistance. Mice with adipose tissue-specific knockout of ANXA1 exhibit exacerbated obesity and metabolic disorders after HFD. In contrast, the global overexpression of ANXA1 effectively prevents HFD-induced obesity and metabolic dysfunction in mice. As ANXA1 protein and mRNA levels gradually decrease with the differentiation of SVFs, we believe that ANXA1 plays an important role in the development stage of adipocytes.

Next, we investigate how ANXA1 contributes to obesity in SVFs from adipose tissue. The findings demonstrate that ANXA1 competes with the E3 ubiquitin ligase MYCBP2 for binding to PDLIM7. We find that ANXA1 in SVFs interacts with PDLIM7, thereby decreasing the interaction between PDLIM7 and MYCBP2. When the interaction between PDLIM7 and MYCBP2 weakens, MYCBP2-mediated SMAD4 K48-linkage specific ubiquitination increases, leading to an increase in SMAD4 degradation through the proteasomal pathway and a decrease in SMAD4 protein levels. It has been reported that in BMP/TGF-β signaling, BMP4 binds its receptor and signals to activate the downstream transcription factor SMAD4, leading to the transcription of PPARγ and facilitating of adipogenesis.^44,45^ Our study reveals that ANXA1 reduces SMAD4 protein accumulation through protein-protein interactions (PPIs), inhibits PPARγ transcription, and ameliorates obesity and metabolic disorders (Fig. 7k). In addition, Ac2-26, the active N-terminal peptide of ANXA1, interacts with PDLIM7, reduces SMAD4 protein levels in SVFs and inhibits the differentiation potential of SVFs. Ac2-26 treatment also prevents HFD-induced obesity in mice. PPIs are essential for most biological processes governing life.^46^ This study has unveiled PDLIM7 and MYCBP2 as novel potential targets in the fight against obesity and uncovered a novel mechanism whereby ANXA1-PDLIM7-MYCBP2-SMAD4 PPIs influences adipogenesis. The discovery significantly contributes to the expansion of the PPIs-obesity network, offering fresh perspectives for the identification of therapeutic targets in obesity treatment.

Notably, the elevation of ANXA1 in mice and humans with obesity does not seem to support the hypothesis that ANXA1 prevents obesity. However, Sárvári et al.^47^ observed that HFD-induced obesity leads to a considerable relative increase in immune cells and fibro/adipogenic progenitors 2 (FAP2, also known as preadipocytes). The ANXA1 levels in immune cells and preadipocytes were significantly higher than those in mature adipocytes (Fig. 4c). Considering the increased relative cell count of inflammatory cells and preadipocytes with high expression of ANXA1 and the compensatory protective increase in ANXA1 levels in obesity, this may explain the higher ANXA1 levels in the SAT of mice and humans with obesity. Additionally, the anti-inflammatory effects of ANXA1 cannot be ignored. Adipose tissue inflammation is central to metabolic diseases associated with obesity. Our previous studies have showed that a lack of ANXA1 is closely associated with the exacerbation of inflammation in acute aortic dissection^48^ and diabetic nephropathy.^49,50^ We also observed the increased levels of inflammatory factors in SVFs lacking ANXA1 (Supplementary Fig. 6f). These data strongly suggest that the anti-inflammatory effect of ANXA1 on obesity may be substantial and warrants further investigation.

Although our findings indicated that the interaction between ANXA1 and PDLIM7 primarily occurs in the cytoplasm, and the influence of ANXA1 on SMAD4 was mainly through its ubiquitination and proteasomal degradation in the cytoplasm, we observed a notable phenomenon during the ANXA1 immunofluorescence staining of SVFs. A small subset of cells produced vesicles, with ANXA1 predominantly localized at the vesicles surface (Supplementary Fig. 6b). This observation, coupled with documented instances of elevated circulating ANXA1 in obese populations,^23^ leads us to hypothesize that ANXA1 may travel with vesicles throughout the body, exerting an anti-adipogenic effect. Exosomes, such as vesicles, generally function by being captured and endocytosed into the cytoplasm of target cells. This can further explain that the anti-obesity effect of ANXA1 is exerted intracellularly, not through extracellular receptors. The potential mechanism represents a significant interest for our future research.

There are currently nine known types of ubiquitination based on the connection sites between ubiquitin and substrates. These include M1, K6, K11, K27, K29, K33, K48, K63, and G76. Each type of ubiquitination regulates different cellular functions. In particular, K48-linked ubiquitination is associated with proteasomal degradation. K48 linkages are the most common type, accounting for approximately 50% of all ubiquitin chains. K6 ubiquitin chains may be involved in processes such as DNA damage repair and mitochondrial autophagy. Additionally, the Lys48/Lys11 branched chain formed by K11 and K48 linkages enhances proteasomal degradation. K63-linked ubiquitination and mono-ubiquitination may be involved in DNA repair and endocytosis.^51^ We employed co-IP techniques to investigate K48-linkage specific ubiquitination and K63-linkage specific ubiquitination of SMAD4 in SVFs (silencing ANXA1, PDLIM7, or MYCBP2 using siRNA). Interestingly, we observed significant changes in K48-linkage specific ubiquitination of SMAD4, while K63-linkage specific ubiquitination remained unchanged. Based on the results, we propose that MYCBP2 primarily promotes SMAD4 degradation through K48-linkage specific ubiquitination. However, we need more in-depth experiments to explore whether SMAD4 has other ubiquitination sites.

The effect of adipogenesis on obesity remains controversial. The prevailing view among many researchers is that adipogenesis contributes to the development of obesity.^52,53^ However, a counter-argument suggests that additional expansion of adipose tissue could potentially enhance metabolic health in individuals afflicted with obesity.^12,54^ Our findings align with the majority perspective, demonstrating that the absence of ANXA1 accelerates the transformation of preadipocytes into adipocytes. This process facilitates the expansion of adipose tissue in mice, thereby promoting obesity.

In conclusion, our study uncovers that ANXA1 prevents obesity and reduces insulin resistance. Mechanistically, ANXA1 inhibits the interaction between PDLIM7 and MYCBP2, thereby promoting MYCBP2-mediated SMAD4 ubiquitination and degradation to inhibit adipogenesis. Our study reveals molecular mechanisms linking ANXA1 to adipose tissue homeostasis and obesity for the first time, and provides insights for potential interventions in metabolic disorders.

## Materials and methods

### Human subjects

This study was conducted in accordance with both the Declaration of Helsinki and the International Conference on Harmonization Guidelines for Good Clinical Practice. The study protocols were approved by the Ethics Committee of The Affiliated Hospital of Southwest Medical University. This study included 10 participants who were divided into lean control (lean, *n* = 5) and obese patient (obese, *n* = 5) groups based on clinical assessment. All the samples were obtained from discarded SAT from the donor site of scar surgery and snap frozen in liquid nitrogen for the extraction of total RNA and protein. The experiments were carried out with the full, informed consent of the subjects. A brief description of the participants is found in Supplementary Table 1.

### Animals

All animal experiments were performed in accordance with the regulations approved by the Peking University Institutional Animal Care and Use Committee and followed the National Institute of Health guidelines on the care and use of animals. *Anxa1*-knockout (*Anxa1*^*KO*^), *Anxa1* flox/flox (*Anxa1*^*fl/fl*^) and *Anxa1* transgenic (*Anxa1*^*Tg*^) mice were generated as Zhou et al. described.^48^ Male C57BL/6 mice (*WT*) were used as controls. *Adipoq*-Cre mice were purchased from Suzhou Cyagen Biotechnology Co., Ltd. *WT* mice, Male db/db mice and db/m mice were purchased from the Laboratory Animal Center of Peking University Health Science Center. Eight-week-old male mice with different genotypes were fed a high-fat diet (D12492, Research Diets) for 10–18 weeks to induce diet-induced obesity. All animals were housed at 21 ± 1 °C with a humidity of 50% ± 5% in a 12-h light/dark cycle and had ad libitum access to standard mouse feed and water throughout the experiments. Animal welfare and experimental procedures were carried out strictly in accordance with ethical regulations of the European Parliament on the protection of animals used for scientific purposes.

### In vivo mouse treatments

Ac2-26 (Ac-AMVSEFLKQAWFIENEEQEYVQTVK, MCE, HY-P1098) was administered by intraperitoneal injection into db/db mice at doses of 0 mg, 0.5 mg, 1.0 mg, or 2.0 mg/kg body weight every other day, starting two days prior to the experiment and continuing for up to 10 weeks.

PPARγ antagonist, GW9662(MCE, HY-16578), was dissolved in dimethyl sulfoxide (DMSO) and stored at −20 °C. The aliquots were diluted with PBS to a final concentration of 10% DMSO and administered by intraperitoneal injection at 1 mg/kg for GW9662 every other day, starting two days prior to the experiment and continuing for up to 16 weeks.

### Metabolic phenotyping

Plasma cholesterol (100000180; BioSino), triglycerides (TG) (100000220; BioSino), and glucose (100000240; BioSino) were measured using enzymatic methods. Free glycerol content (E1002; Applygen, Beijing, China) and the levels of non-esterified fatty acid (NEFA) were measured by a colorimetric assay (294-63601; Wako Chemical, Richmond, VA).

Body fat and lean mass were measured using a nuclear magnetic resonance (NMR) analyzer (Minispec LF90II, Bruker Optics).

Glucose tolerance testing has been described by Zhao et al.^55^

Body composition was measured with an EchoMRI-100 body composition analyzer (Echo Medical Systems, Oceanside, NY).

Enclosed metabolic chambers with digital oxygen (O_2_) and carbon dioxide (CO_2_) sensors (TSE Systems) were used to simultaneously measure energy expenditure and indirect calorimetry in mice under standard housing conditions.

### Histology and analysis of liver lipids

The hematoxylin and eosin (H&E) staining of adipose tissue and livers samples and analysis of liver lipids have been described by Zhao et al.^55^

### Cell culture and induction of differentiation

Mouse SVFs of WAT was isolated from 8-week-old mice. The tissue was minced and digested in a sterile solution of 0.8 mg/ml type I collagenase (Biosharp, BS032A) in DMEM(L) medium (Solarbio, 31600) containing 1% bovine serum albumin (BSA, Carl Roth GmbH). After 1 h of digestion at 37 °C with gentle agitation, DMEM(L) was added to reach approximately 200% of the total volume. The digest was filtered through a 100 µm cell strainer (Fisher Scientific, Schwerte, Germany) to remove undigested tissue fragments. The lower layer of culture medium was collected and centrifuged at room temperature and 500 g for 20 min. The supernatant was removed and the SVFs were resuspended in DMEM/F12 medium (Procell, PM150312) containing 10% fetal bovine serum (FBS; Thermo, 26010074). The SVFs were cultured in DMEM/F12 containing 10% FBS, and the medium was changed every two days.

On day 0, SVFs were induced to differentiate using DMEM/F12 containing 10% FBS, 170 nM insulin (Solarbio, I8830), 1 μM dexamethasone (Solarbio, D8040), 250 μM 3-isobutyl-1-methyl-xanthine (Aladdin, I106812), and 60 μM indomethacin (Konoscience, A086907). The medium was changed every two days until lipid droplets appeared.

### Lentivirus production

HEK 293T cells were seeded 1 day before transfection. At 70% confluency, cells were transfected using jetPRIME® (Polyplus, 101000046) with lentiviral-based transfer vectors (Supplementary Table 2) and lentivirus packaging (psPAX2) and envelope plasmids (pMD2-VSVG). Supernatants were collected at 24-, 48-, and 72-h post-transfection and filtered through a 0.45-mm PVDF filter (Millipore). Polybrene (4 µg·ml^−1^) was added to the viral supernatants for infection of SVFs. The lentiviral vector plasmids were constructed by Shanghai Genechem Co., Ltd.

### Cell transfection (plasmids, siRNA, adenoviruses and lentivirus)

When the cell density was between 40 and 60%, SVFs and HEK293T cells were transfected with plasmids and siRNA using jetPRIME® (Polyplus, 101000046) following the manufacturer’s instructions. The plasmid sequence is listed in Supplementary Table 2, and the siRNA sequence is listed in Supplementary Table 3. The plasmids were constructed by Beijing Tsingke Biotech Co., Ltd, and the siRNA was constructed by Suzhou GenePharma Co., Ltd.

SVFs were seeded in 6-well plates at a density of 1 × 10^6^ cells/well. When the cell density was between 40 and 60%, SVFs were transfected with Ad-NC, Ad-SMAD4, Ad-MYCBP2, LV-NC, LV-SMAD4 and LV-PDLIM7 at MOI = 400. After 24 h, complete culture medium was used, and GFP fluorescence detection was performed at 1d, 2d, 3d, 5d, 8d and 11d to determine the transfection effect. The adenoviruses and lentivirus sequence are listed in Supplementary Table 2. The adenoviruses were constructed by Shandong Weizhen Biotechnology Co., Ltd.

### Co-immunoprecipitation (co-IP) assays and mass spectrometry

For co-immunoprecipitation (co-IP) assays, protein lysates were prepared from SVFs, mature adipocytes or H5V cells using Pierce™ IP Lysis Buffer (Thermo, 87787). The lysates were incubated overnight at 4 °C with anti-ANXA1(Abcam ab214486, 1:100), anti-PDLIM7 (Proteintech 10221-1-AP, 1:100), anti-MYCBP2 (Millipore MABN2397, 1:50), anti-SMAD4 (Proteintech 10231-1-AP, 1:100) antibodies or control immunoglobulin G (IgG; Beyotime, A7058) on a rotating pattern. Immobilized Protein A/G resin slurry (Thermo, 20423) was added to the lysates and incubated for 2 h at 4 °C with gentle mixing. The complexes were washed five times with lysis buffer (Thermo, 28379) and resuspended in 2× SDS loading buffer. The immunoprecipitated proteins were eluted by incubation at 95 °C for 5 min and detected by immunoblotting after separation by SDS-PAGE.

For mass spectrometry-based proteomic analysis, gel pieces were dehydrated with acetonitrile and digested with trypsin. LC-MS/MS analysis of peptides was conducted at Peking University Institute of Systems Biomedicine (China).

### Immunofluorescence

SVFs were 4% paraformaldehyde-fixed. Standard IF was performed with Quadruple Fluorence immunohistiochemical mouse/rabbit kit (pH 9.0) (Immunoway, RS0037-30t), anti-ANXA1(Abcam, ab214486, 1:100), anti-PDLIM7 (Proteintech, 10221-1-AP, 1:100), anti-MYCBP2 (Millipore MABN2397, 1:50), anti-SMAD4 (Proteintech, 10231-1-AP, 1:100) and anti-CD105(Proteintech, 67075-1-Ig, 1:1000) antibodies according to the manufacturer’s instructions.

FITC-Ac2-26(Ac-AMVSEFLKQAWFIENEEQEYVQTVK-FITC) with purity higher than 95% used in this study were commercially synthesized by Wuhan Bioyeargene Biotechnology Co. Ltd. (Wuhan, China). The synthesized FITC-Ac2-26 was analyzed by mass spectrometry and co-eluted with native RL-QN15 to ensure the correct structure. SVFs were incubated in a dark environment with FITC-Ac2-26 for 48 h, and then perform fluorescence staining of PDLIM7.

### Three-dimensional image simulation of protein interaction

Utilizing the predicted three-dimensional structures of ANXA1 (P04083 · ANXA1_HUMAN, PDB AF-P04083-F1), PDLIM7 (Q9NR12 · PDLIM7_HUMAN, PDB AF-Q9NR12-F1), SMAD4 (Q13485 · SMAD4_HUMAN, PDB AF-Q13485-F1), and MYCBP2 (O75592 · MYCBP2_HUMAN, PDB 6T7F) obtained from the Uniprot database, we employed Pymol to simulate the interactions between these proteins.

### Ubiquitination assays

The cell-permeable proteasome inhibitor MG132 (10 μM, MCE, HY-13259) was added to SVFs for 6 h, followed by lysis with immunoprecipitation lysis buffer for 30 min. The lysates were immunoprecipitated overnight at 4 °C with an anti-SMAD4 antibody or IgG on a rotating pattern. Protein A/G agarose beads were added to the lysates and incubated for an additional 2 h at 4 °C. The beads were washed five times with lysis buffer, and the proteins were released by boiling in 2× SDS loading buffer. The released proteins were analyzed by immunoblotting with an anti-ubiquitin (P37) (Cell Signaling, 58395, 1:1000), anti-K48-linkage specific polyubiquitin (Cell Signaling, 4289, 1:1000) and anti-K63-linkage specific polyubiquitin (D7A11) (Cell Signaling, 5621, 1:1000) antibodies according to the manufacturer’s instructions.

### ChIP-PCR/qPCR

Cultivate SVFs cells in a 10 cm cell culture dish with a dosage of 10 ml of cell culture medium. When the cell density is 80–90%, the cell permeable protein inhibitor MG132 (10 μM, MCE, HY-13259) was added to SVFs for 6 h. Afterwards, add an appropriate amount of formaldehyde directly to the cell culture medium, gently mix until the final concentration is 1%. Immediately incubate at 37 °C for 10 min to crosslink the target protein and corresponding genomic DNA. BeyoChIP ™ Enzymatic Chromatin Immunoprecision (ChIP) Assay Kit with Protein A/G Magnetic Beads (Beyotime, P2083S), SMAD4 Polyclonal Antibody (Protein, 10231-1-AP), Histone H3 Rabbit Polyclonal Antibody (Beyotime, AF7101), and Rabbit IgG (Beyotime, A7016) were used for ChIP. DNA purification was performed using PCR/DNA purification kit (Beyotime, D0033). The obtained ChIP-DNA was subjected to PCR and qPCR detection using *Pparg* promoter region primers. PCR products were separated by 2% (w/v) agarose gel electrophoresis.

### Flow cytometry

After fixing 3 × 10^6^ SVFs cells with 1% paraformaldehyde, incubate cells with cold PBS containing 1% triton to penetrate the cell membrane. Incubate cells at room temperature with 3% BSA/PBS containing 0.1–10 μg/mL anti-ANXA1(Abcam, ab214486) and anti-CD105(Proteintech, 67075-1-Ig) antibodies for 30 min, and wash cells 3 times with cold PBS. Afterwards, cells were incubated with 3% BSA/PBS containing Donkey Anti-Rabbit IgG H&L (Alexa Fluor® 555, Abcam, ab150074) and Donkey Anti-Mouse IgG H&L (Alexa Fluor® 488, Abcam, ab150105) for 30 min, washed three times, and analyzed by flow cytometry using CytoFlex Flow Cytometer (Beckman Coulter, USA).

### RNA isolation and quantitative real-time polymerase chain reaction

Total RNA was isolated from cells, mouse and human tissues using TRIzol reagent (Invitrogen, Carlsbad, California) according to the manufacturer’s instructions. Equal amounts of RNA were reverse-transcribed to cDNA (TransGen Biotech, Beijing, China). Messenger RNA (mRNA) levels were quantified by quantitative PCR with SYBR Green (TransGen Biotech, Beijing, China). Samples were normalized against β-actin mRNA levels. Primers are listed in Supplementary Table 4.

### Western blot

Whole cell protein samples were extracted from split cells, mouse and human tissue samples in RIPA buffer. Protein concentration was quantitatively determined using the Bradford protein assay (Bio Rad, Hercules, CA, USA). Equal amounts of total protein were loaded onto SDS-PAGE gels and transferred onto nitrocellulose membranes. The membranes were blocked in 5% skim milk and incubated at room temperature for 1 h. They were then incubated with primary antibodies against GAPDH (Proteintech, 60004-1-Ig, 1:5000), β-actin (Beyotime, AF0003, 1:1000), ANXA1 (Abcam, ab214486, 1:3000), ADIPOQ (Proteintech, 66239-1-Ig, 1:1000), SMAD4 (Proteintech, 10231-1-AP, 1:1000), P53 (Proteintech, 10442-1-AP, 1:5000), PPARγ (Proteintech, 16643-1-AP, 1:2000), PDLIM7 (Proteintech, 10221-1-AP, 1:1000), MYCBP2 (Millipore MABN2397, 1:500), Ubiquitin (P37) (Cell Signaling, 58395 1:1000), K48-linkage specific polyubiquitin (Cell Signaling, 4289, 1:1000), K63-linkage specific polyubiquitin (D7A11) (Cell Signaling, 5621, 1:1000), DYKDDDDK tag (Proteintech, 20543-1-AP, 1:2000), and HA tag (Proteintech, 51064-2-AP, 1:5000) for 12 h at 4 °C. The membranes were then incubated with HRP-conjugated secondary antibodies appropriate for the species for 1 h at room temperature. Specific immune-reactive protein bands were detected using ECL kits (Pierce, Appleton, WI, USA) and analyzed using Image J.

### Statistical analysis

Statistical analyses were performed using GraphPad Prism 8.0 software (GraphPad Software, San Diego, CA, USA). The data shown are representative of two or three independent experiments. Continuous variables with normal distribution are expressed as means ± standard errors of the mean. Differences between the two groups were analyzed by Student’s *t* test after the demonstration of homogeneity of variance with an F test. Comparisons between three groups were performed with a one‐way analysis of variance and Dunn’s post hoc test. Continuous variables with non‐normal distributions are expressed as medians. Comparisons between groups were performed using the Mann–Whitney U test for nonparametric variables and Fisher’s exact test or chi‐square test for categorical variables. A two‐sided *p*‐value less than 0.05 was used to define statistical significance.

### Supplementary information

Unprocessed western blots

Supplementary figures and Supplementary tables

## Data Availability

The data supporting the findings of this study are available on request from the corresponding author, Lemin Zheng, Email: zhengl@bjmu.edu.cn. The proteomic data in the article has been submitted to the China National Center for Bioinformation (https://ngdc.cncb.ac.cn/omix, OMIX ID: OMIX006798).
