# Supplementary material for: Annexin A1 binds PDZ and LIM domain 7 to inhibit adipogenesis and prevent obesity
Source: Signal Transduct Target Ther. 2024 Aug 23;9:218. doi: 10.1038/s41392-024-01930-0 (PMC11341699; doi:10.1038/s41392-024-01930-0)
Supplement: Supplementary file 1 — Unprocessed western blots [file 41392_2024_1930_MOESM1_ESM.pdf]

Fig. 1

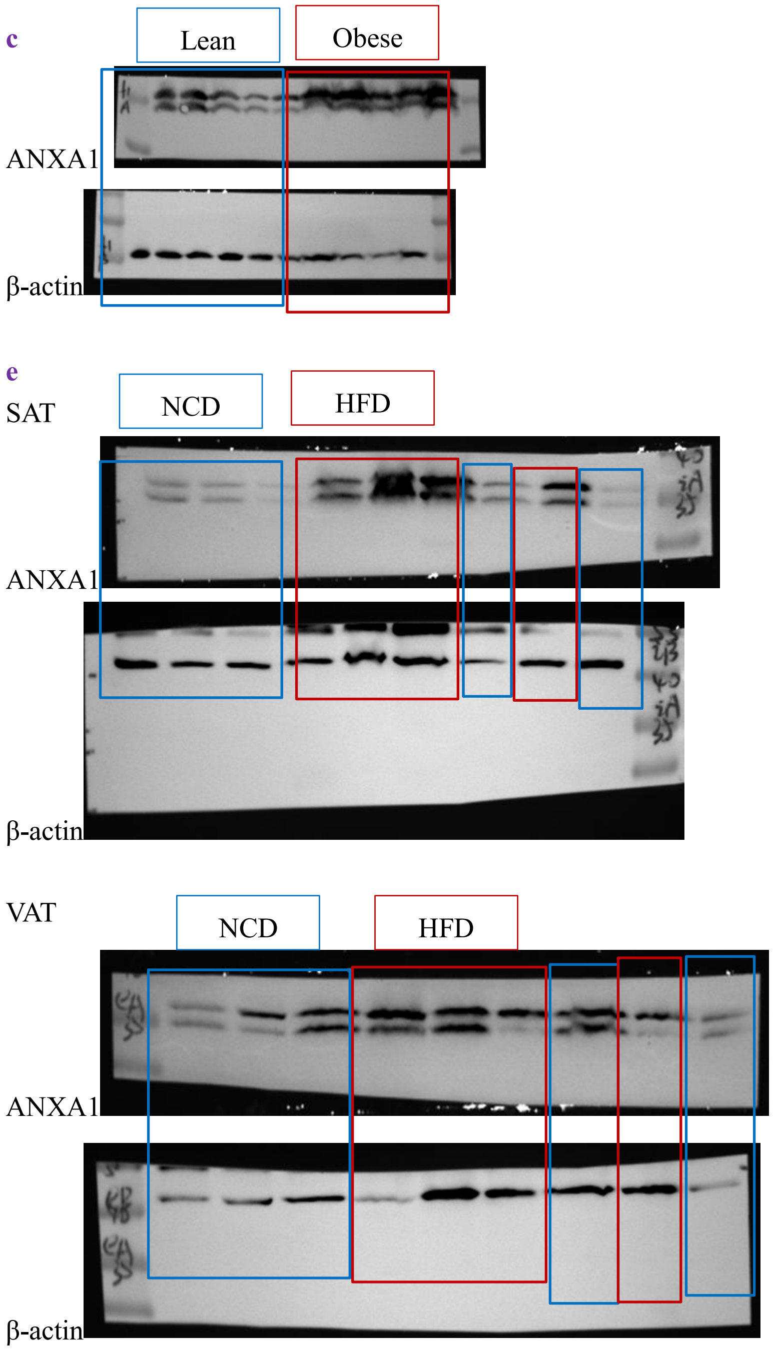
c
Lean
Obese
ANXA1
β-actin
e
SAT
NCD
HFD
ANXA1
β-actin
VAT
NCD
HFD
ANXA1
β-actin

Fig. 4

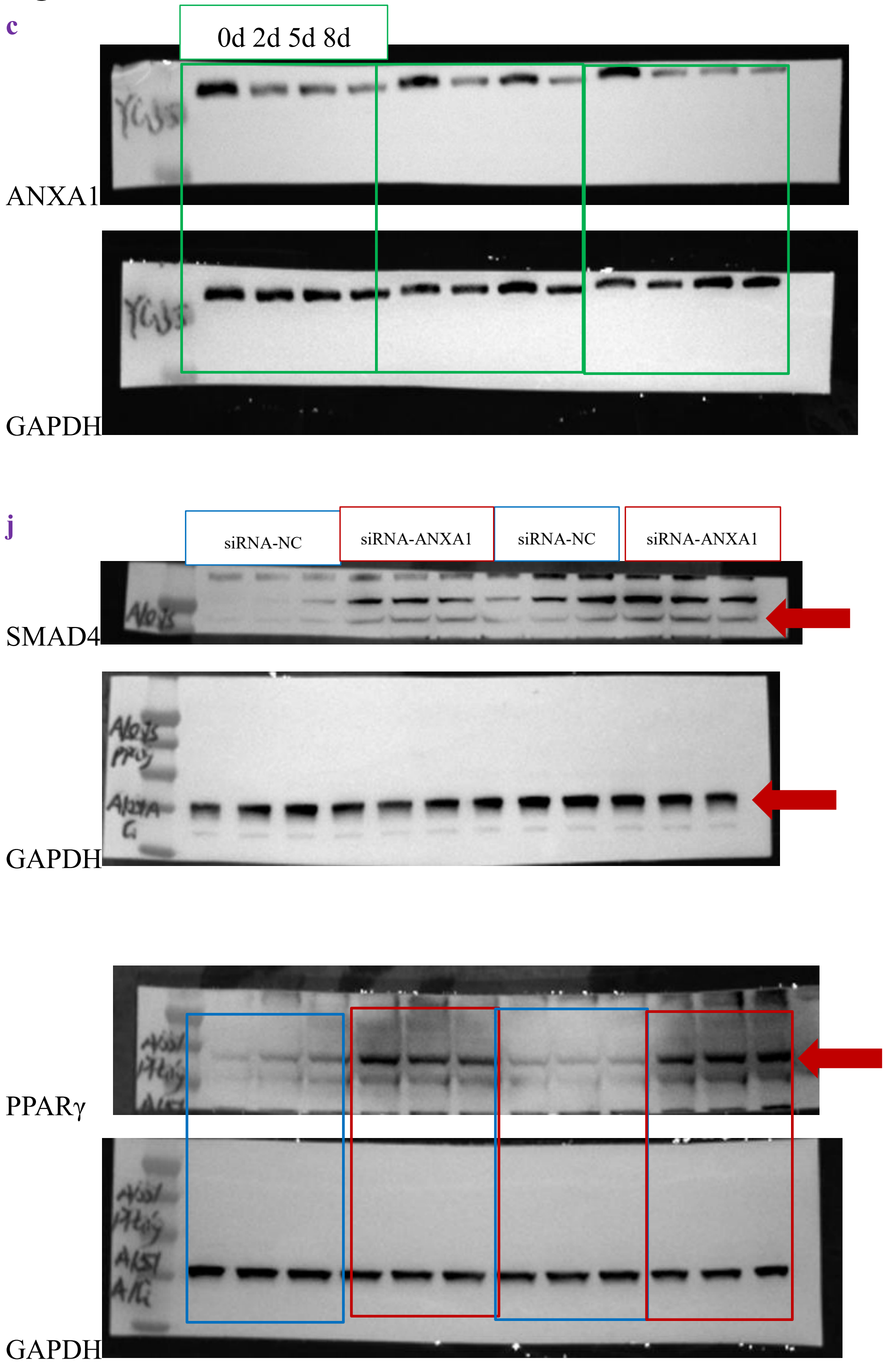
c
0d 2d 5d 8d
ANXA1
GAPDH
j
siRNA-NC
siRNA-ANXA1
siRNA-NC
siRNA-ANXA1
SMAD4
GAPDH
PPARγ
GAPDH

l

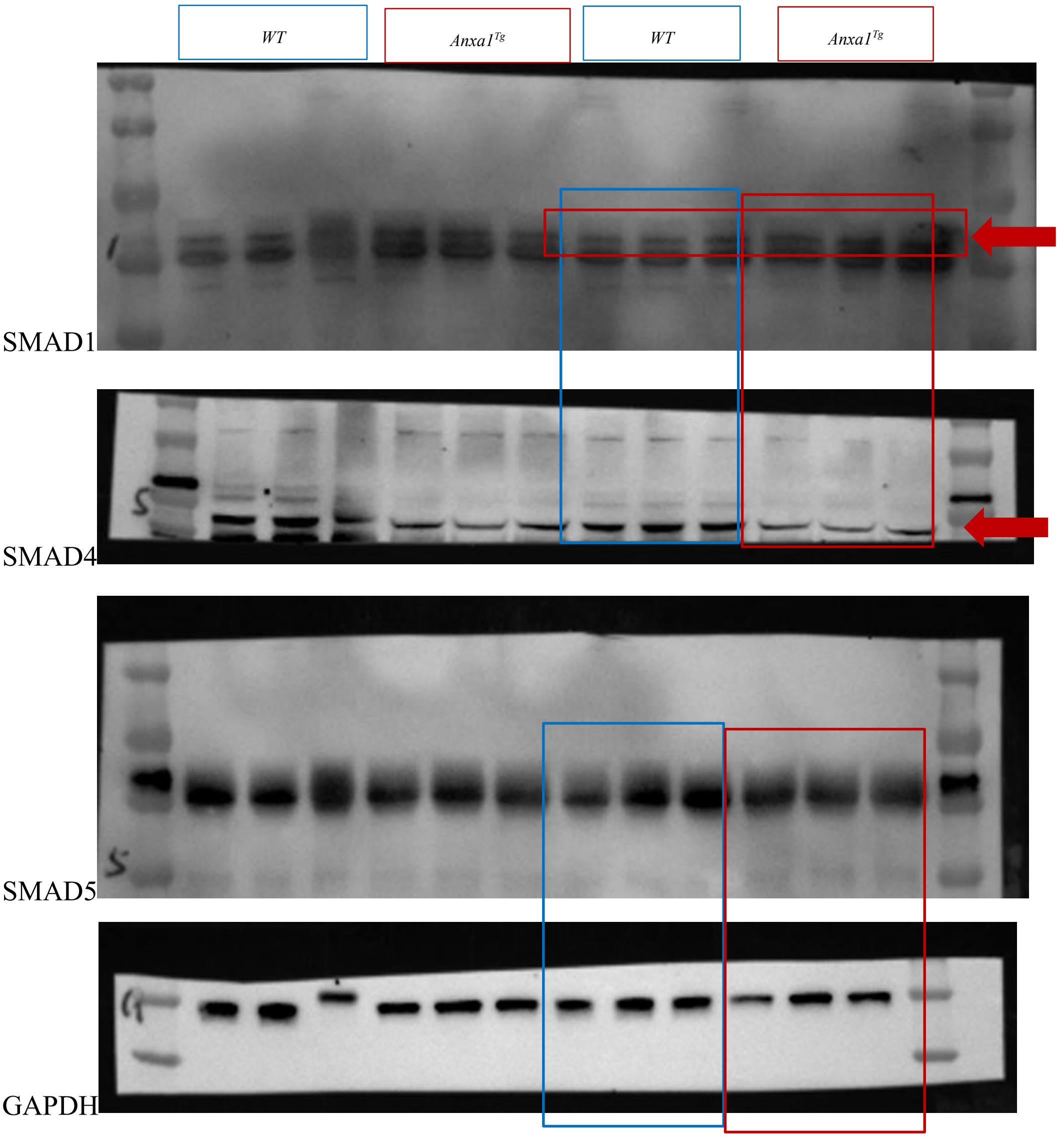

m

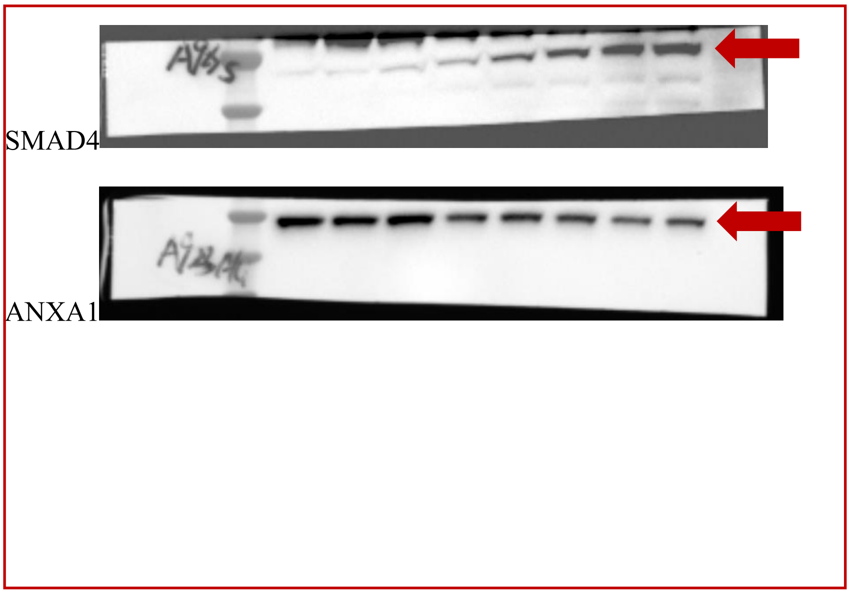

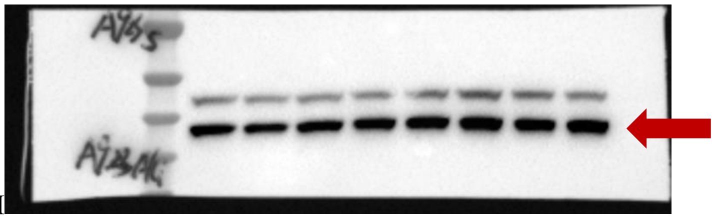

GAPDH

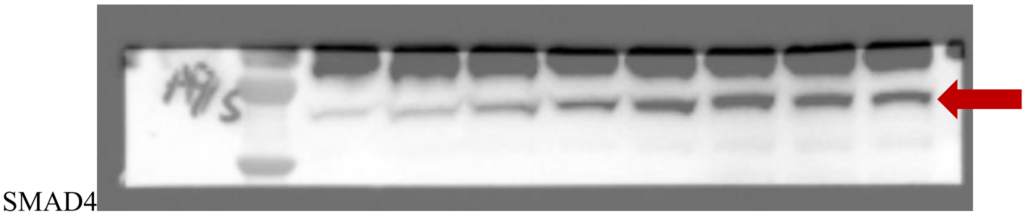

SMAD4

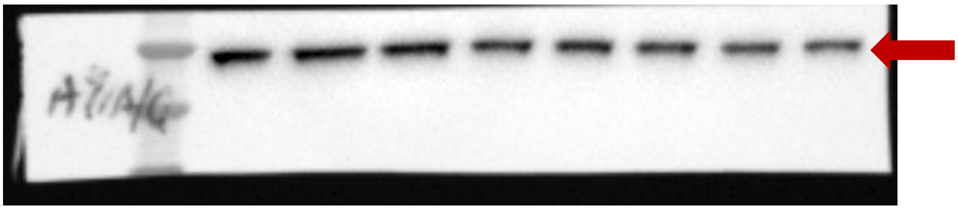

ANXA1

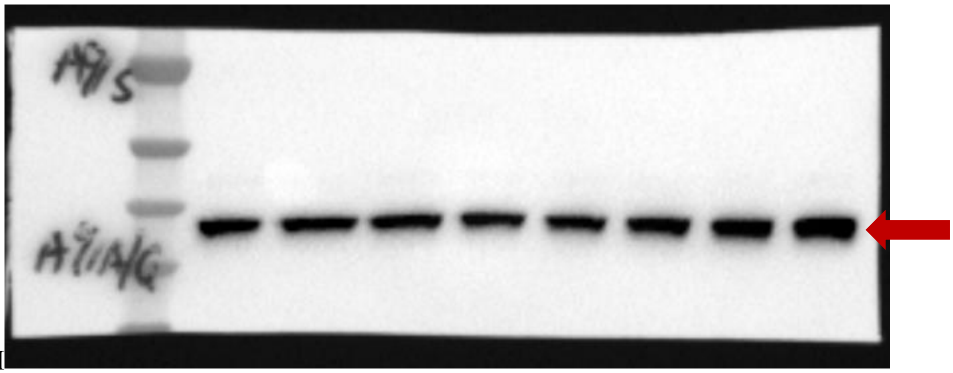

GAPDH

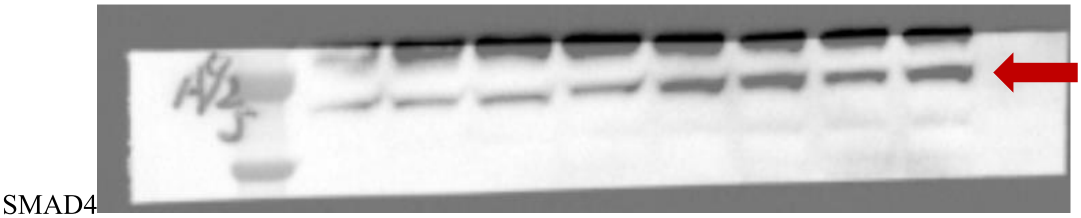

SMAD4

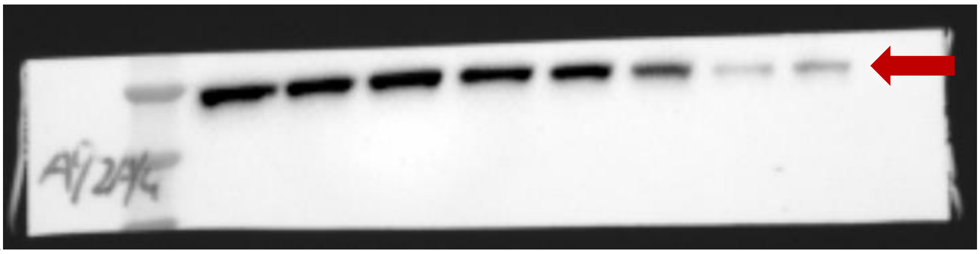

ANXA1

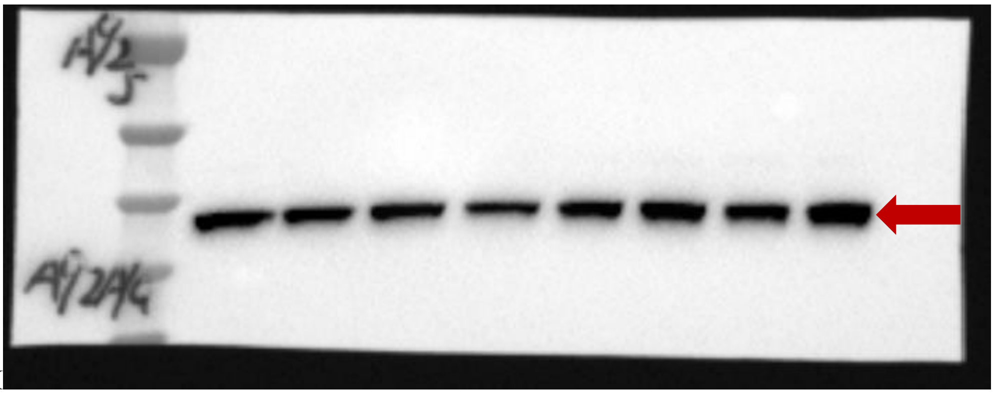

GAPDH

Fig. 5

b

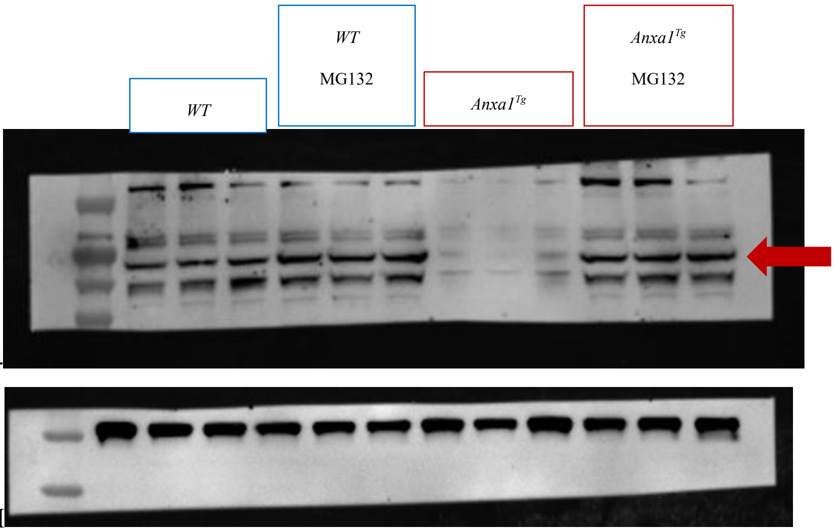

SMAD4

GADPH

c

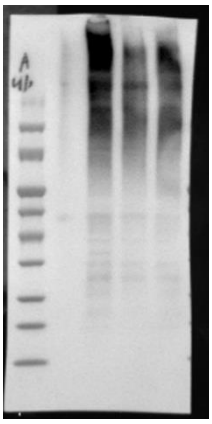

Ub

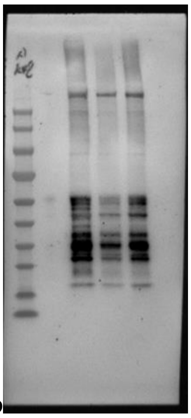
K^{48}-Ub

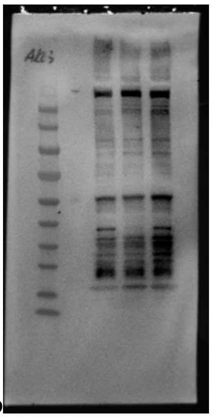
K^{63}-Ub

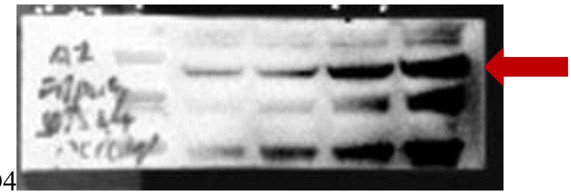
SMAD4

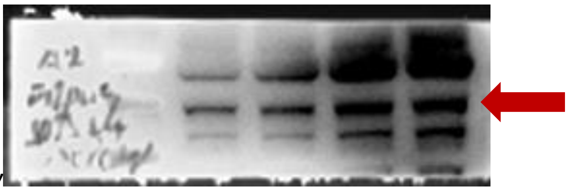
PPARγ

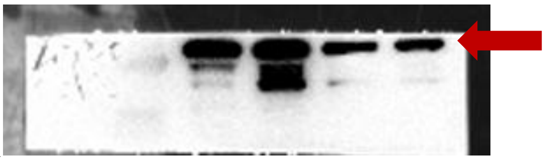
ANXA1

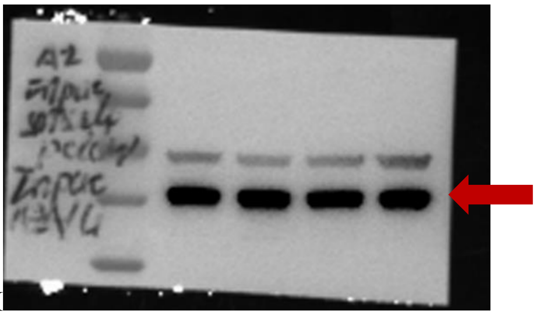
GAPDH

e

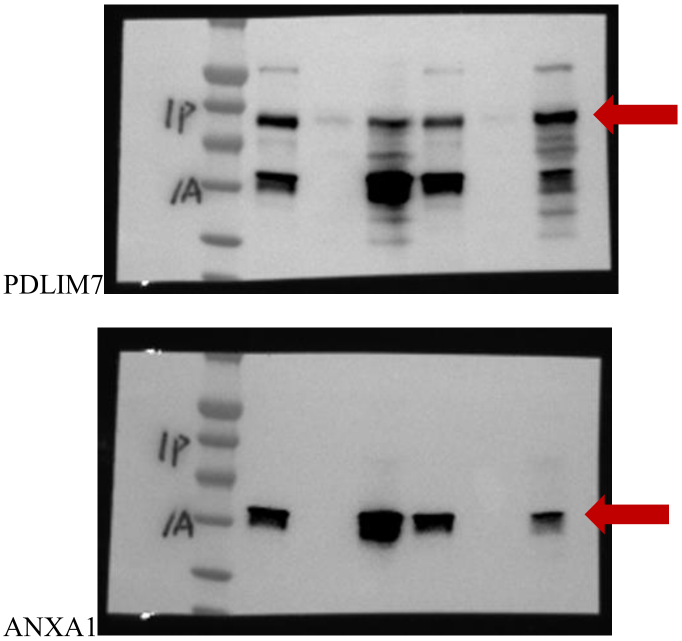
PDLIM7
ANXA1

f

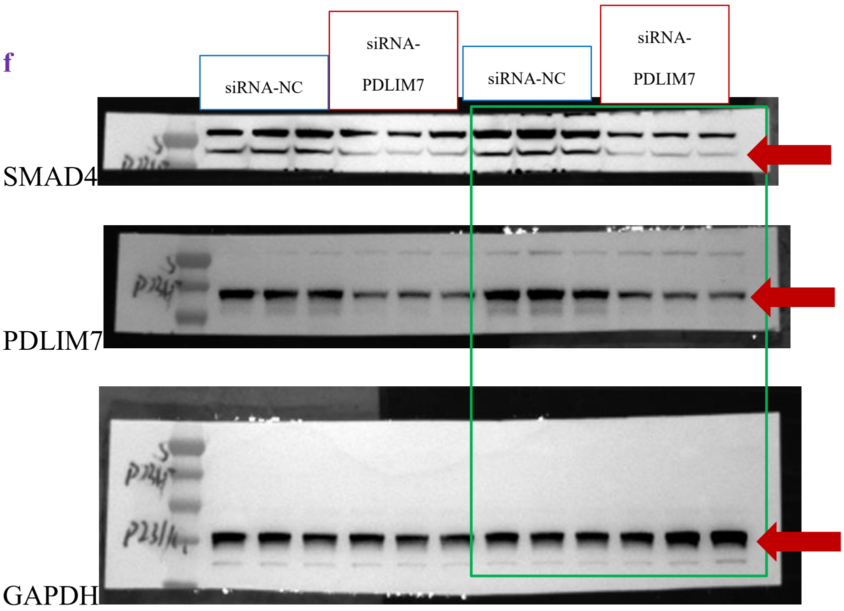
siRNA-NC
siRNA-PDLIM7
siRNA-NC
siRNA-PDLIM7
SMAD4
PDLIM7
GAPDH

i

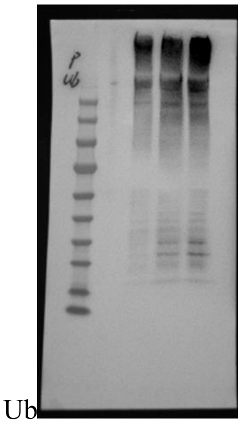
Ub

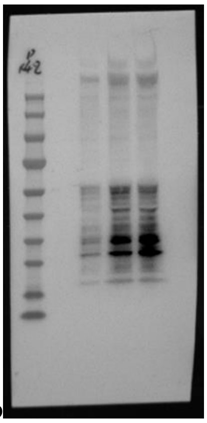

K^{48}-Ub

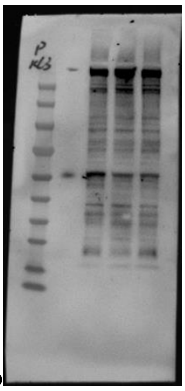

K^{63}-Ub

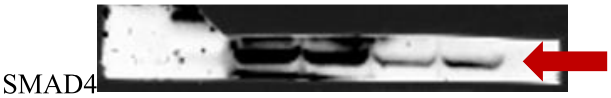

SMAD4

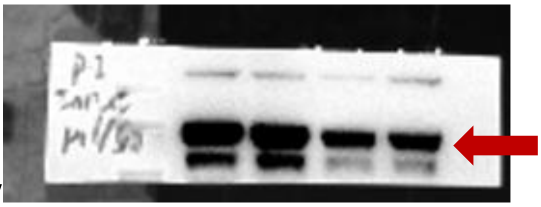

PDLIM7

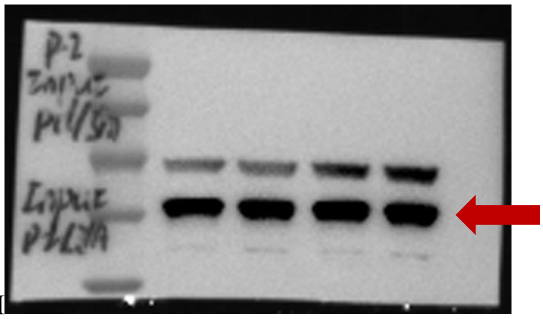

GAPDH

k

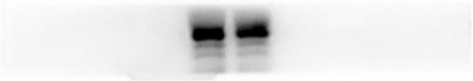

anti-Flag HA

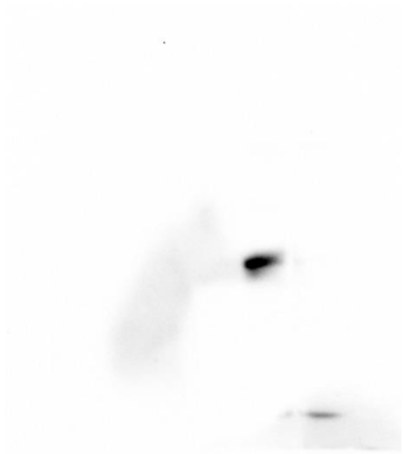

anti-HA Flag

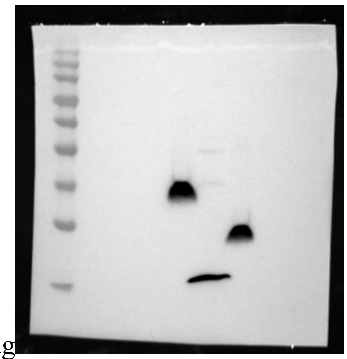

Input-Flag

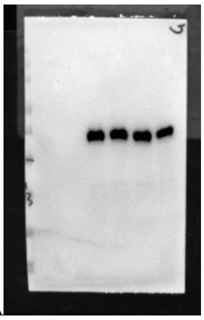

Input-HA

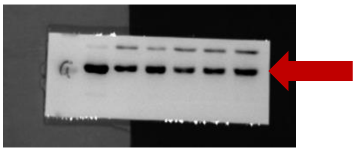

Input-GAPDH

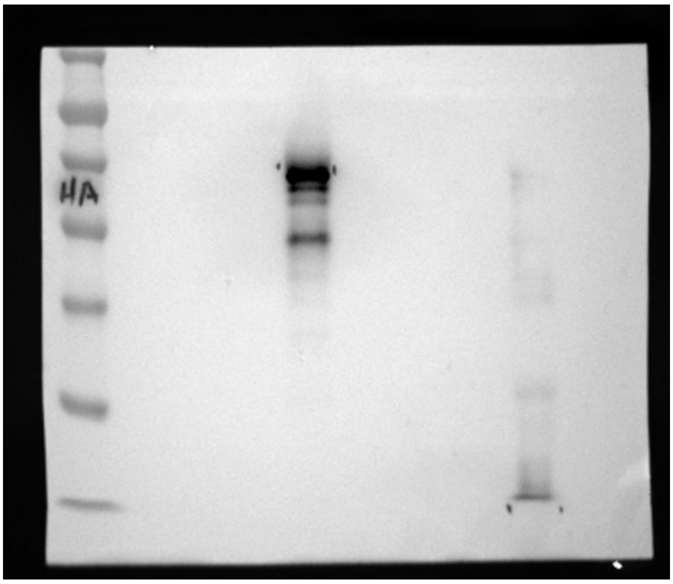

anti-Flag HA

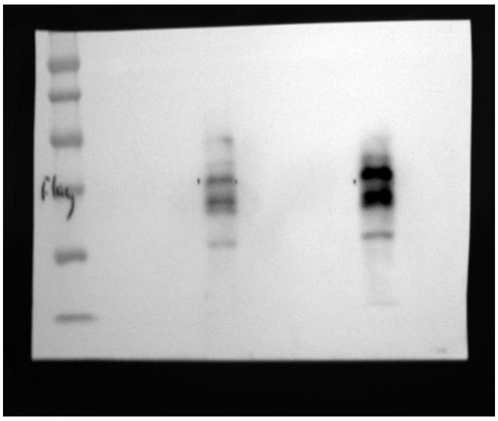

anti-HA Flag

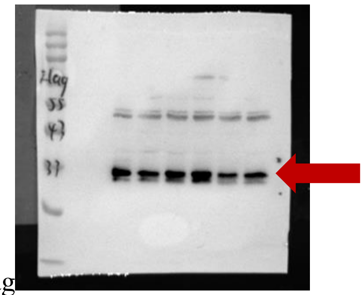

Input-Flag

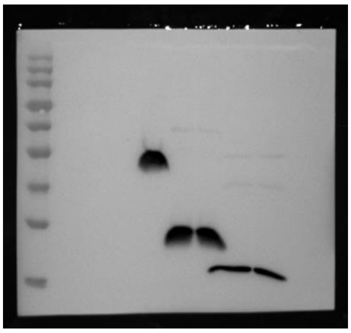

Input-HA

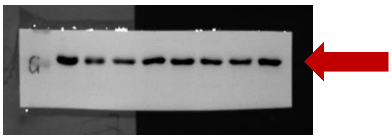

Input-GAPDH

Fig. 6

b

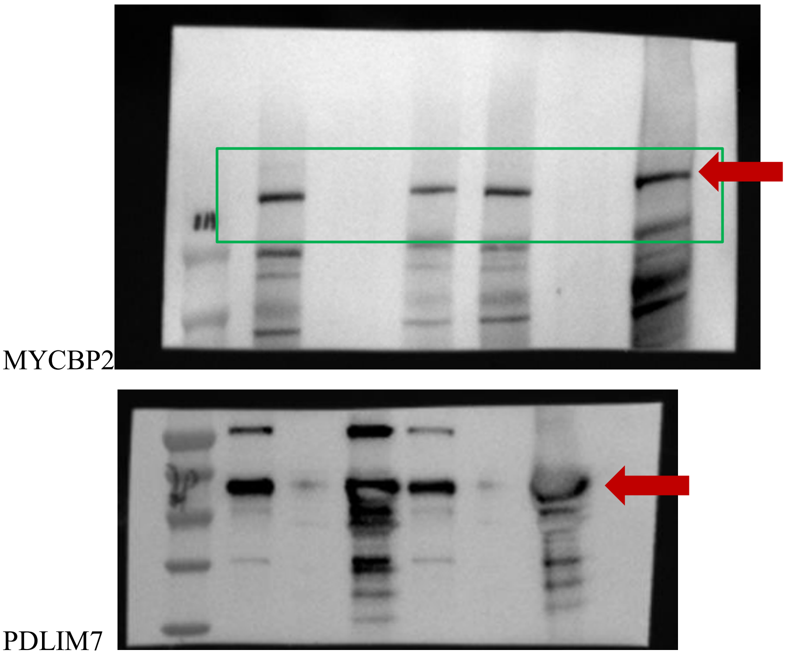
MYCBP2
PDLIM7

c

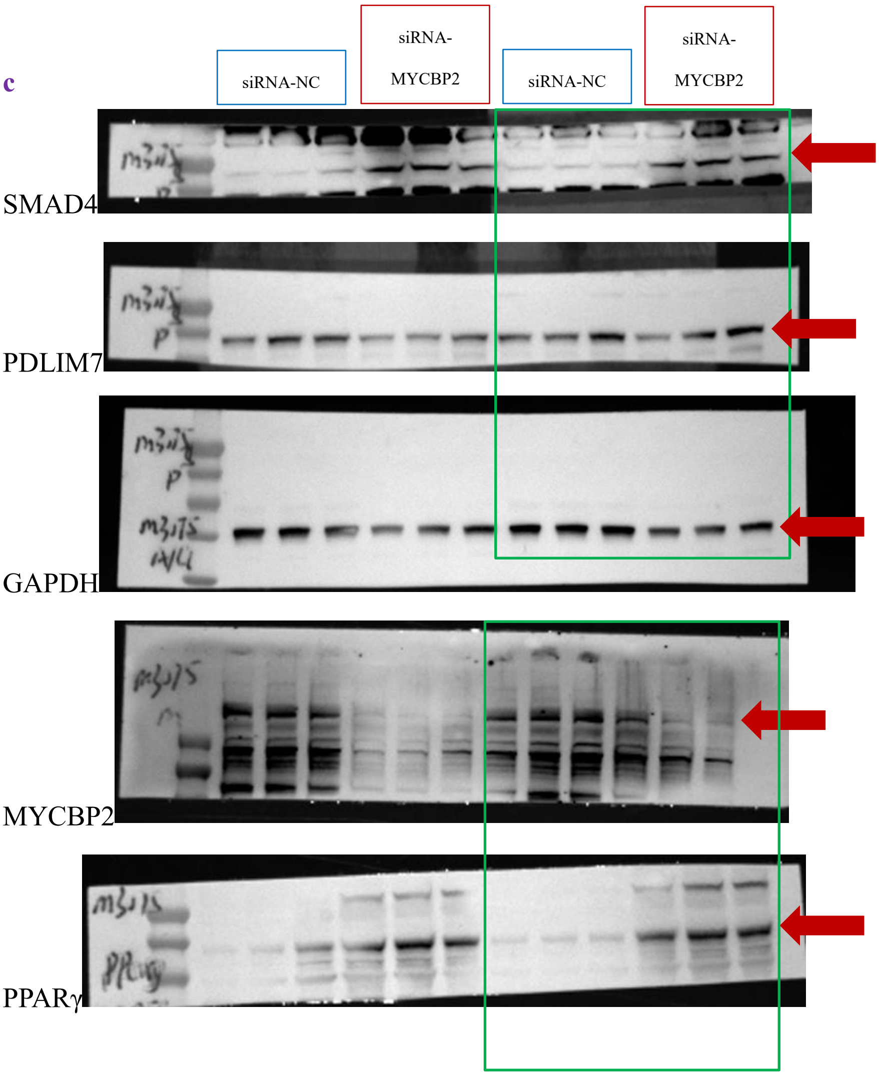
siRNA-NC
siRNA-MYCBP2
siRNA-NC
siRNA-MYCBP2
SMAD4
PDLIM7
GAPDH
MYCBP2
PPARγ

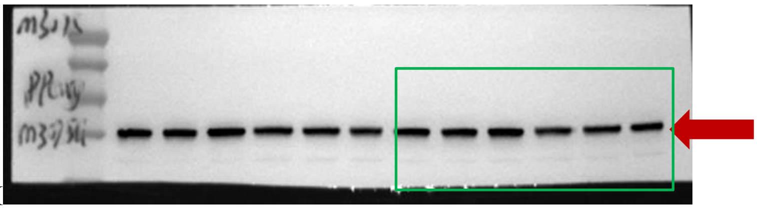

GAPDH

f

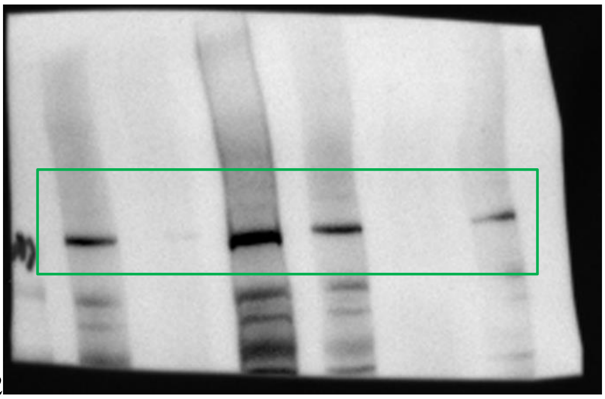

MYCBP2

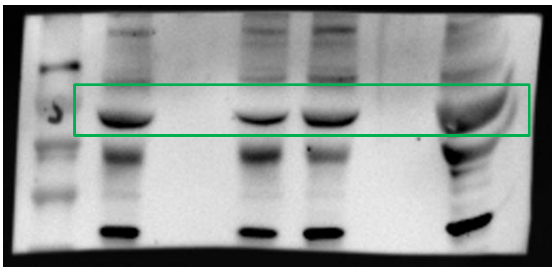

SMAD4

g

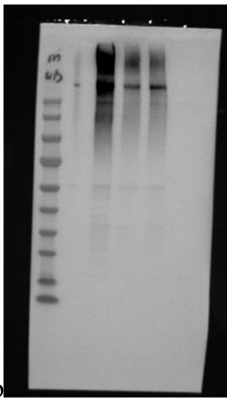

Ub

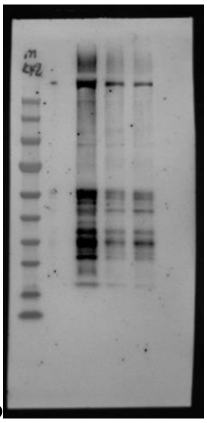

K^{48}-Ub

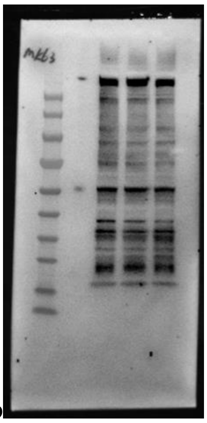

K^{63}-Ub

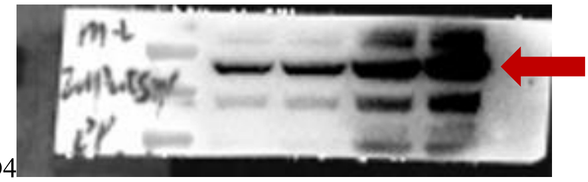

SMAD4

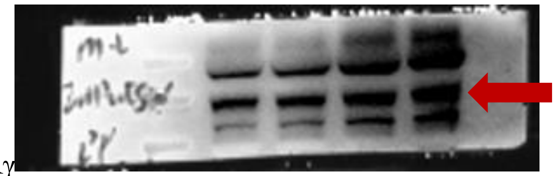

PPARγ

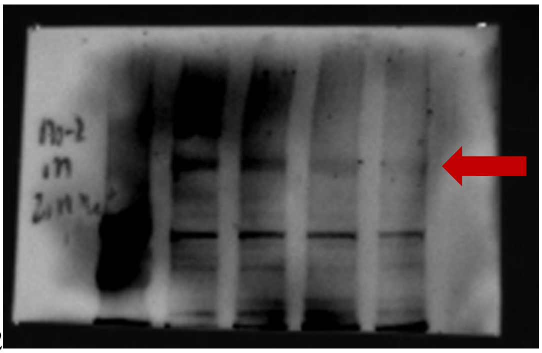

MYCBP2

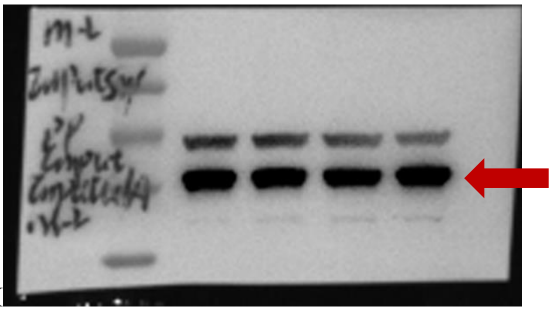

GAPDH

j

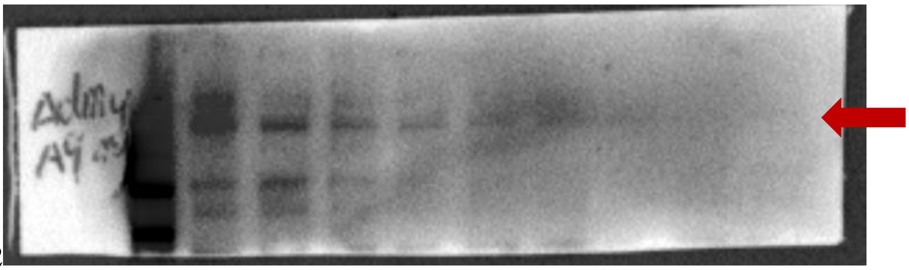

MYCBP2

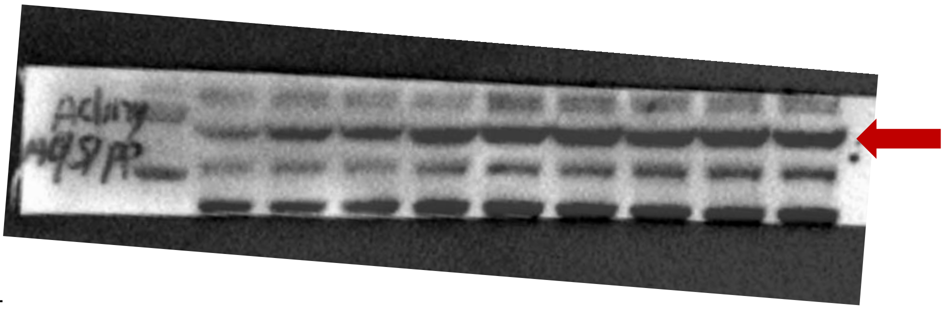

SMAD4

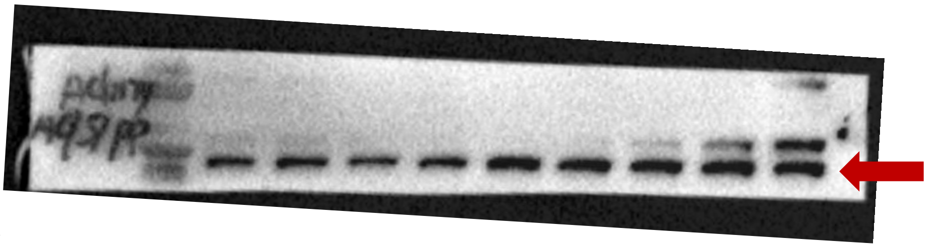

PPARγ

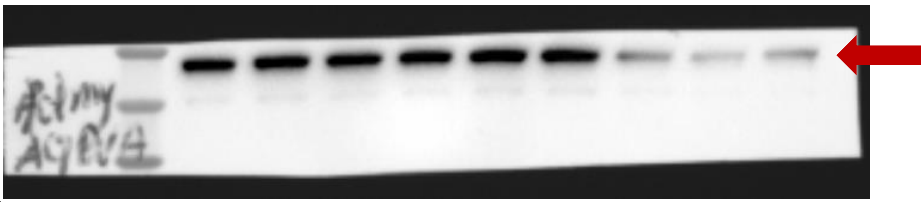

ANXA1

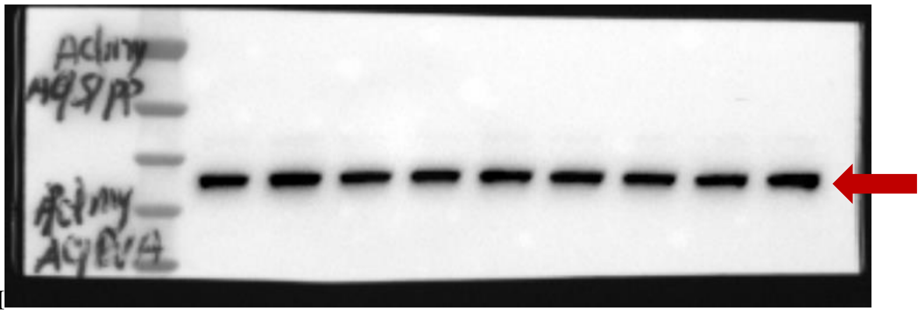

GAPDH

l

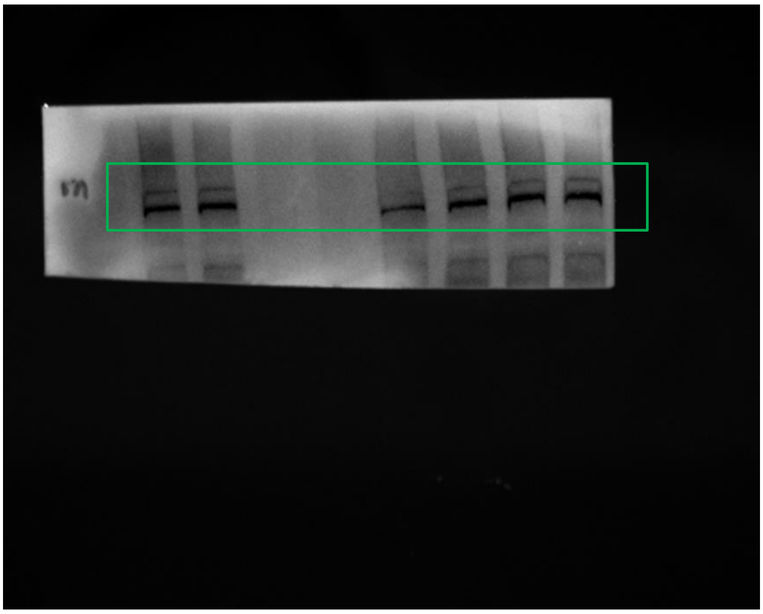

MYCBP2

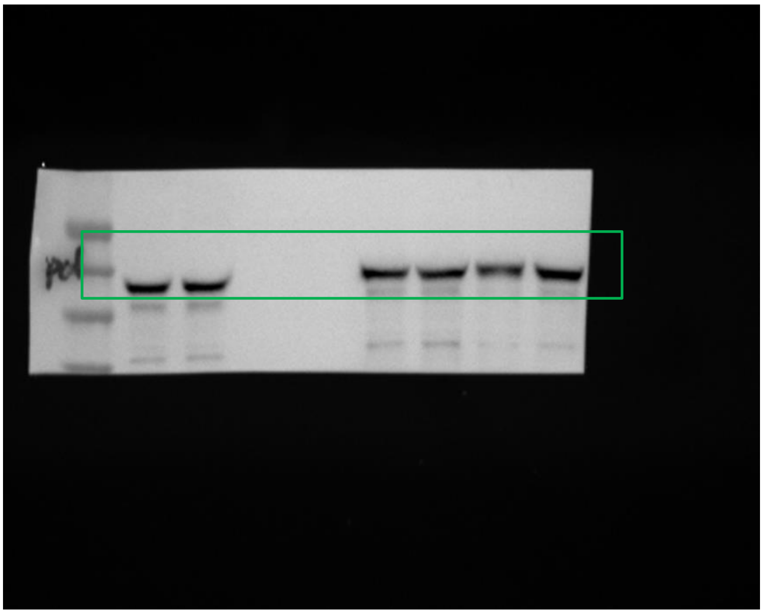

PDLIM7

m

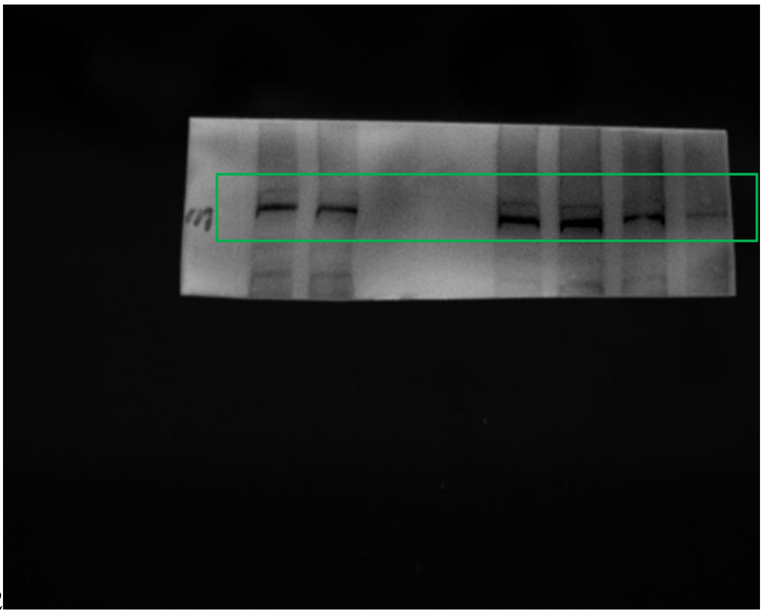

MYCBP2

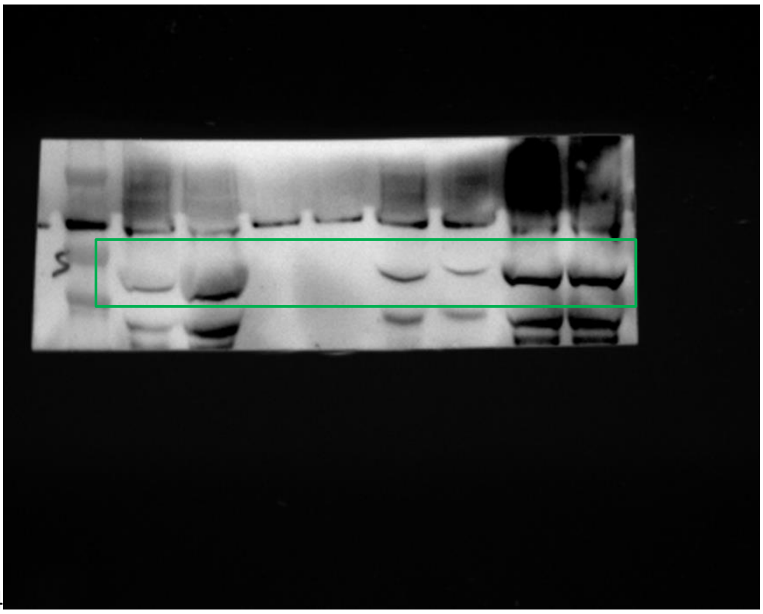

SMAD4

n

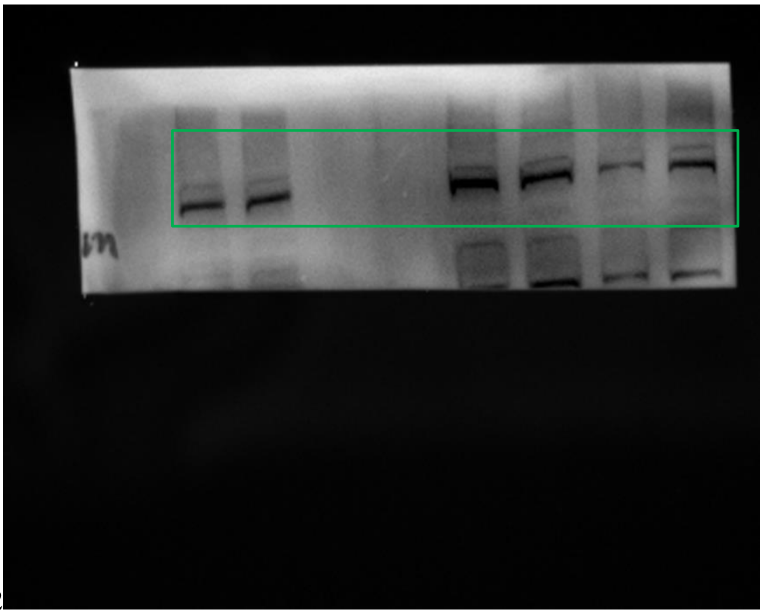

MYCBP2

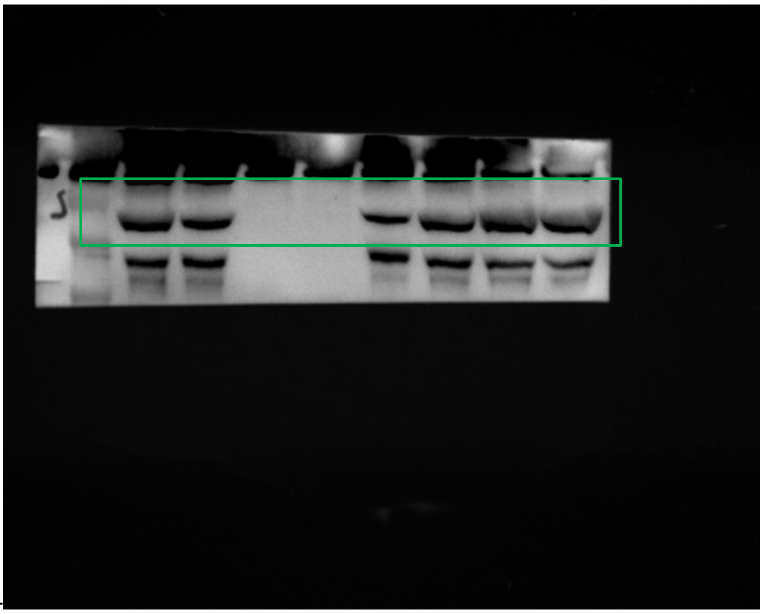

SMAD4

I

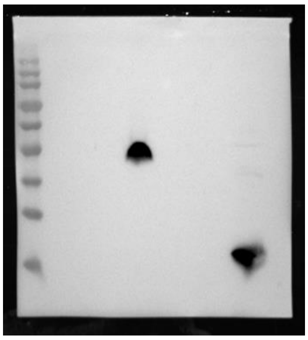

anti-Flag HA

anti-HA Flag

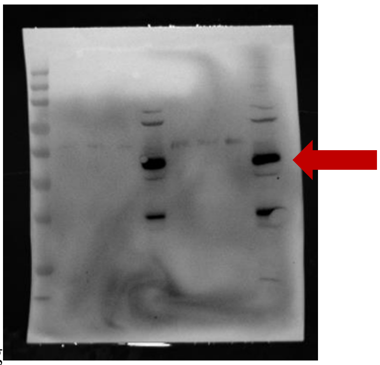

Input-Flag

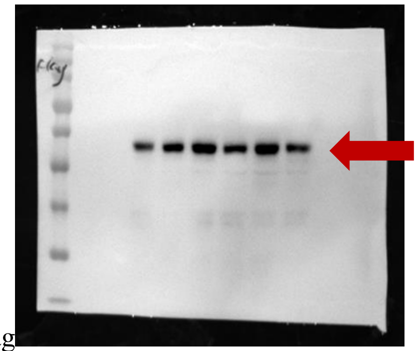

Input-HA

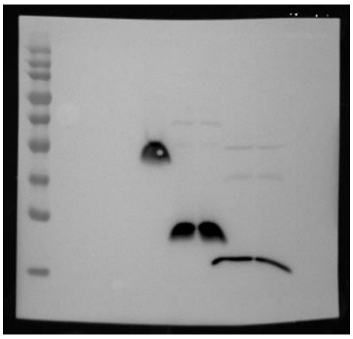

Input-GAPDH

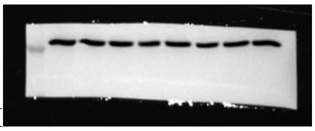

Fig. 7

a

SMAD4

GAPDH

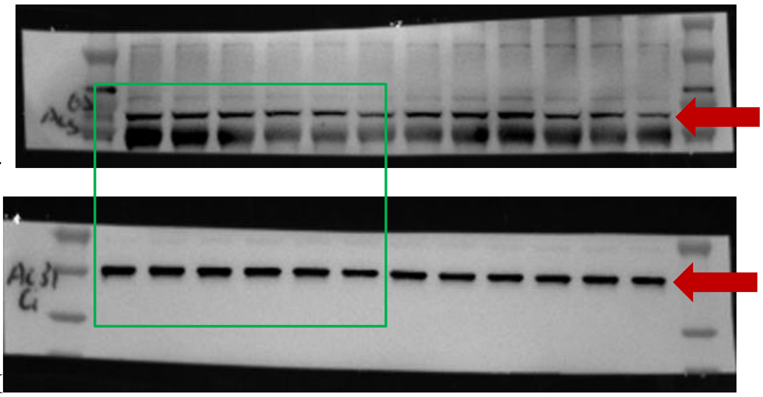

Fig. S1

a

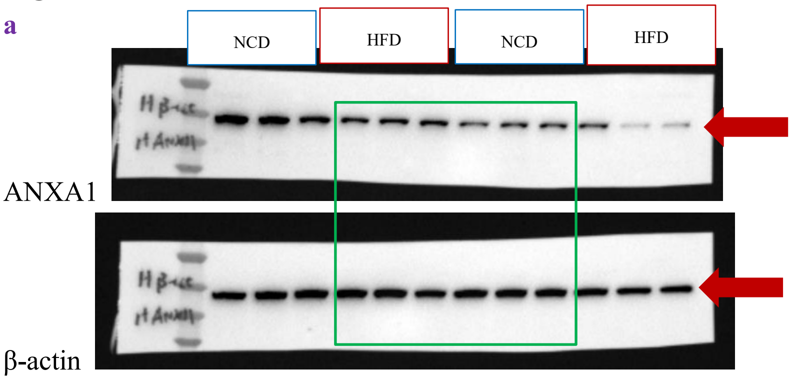

b

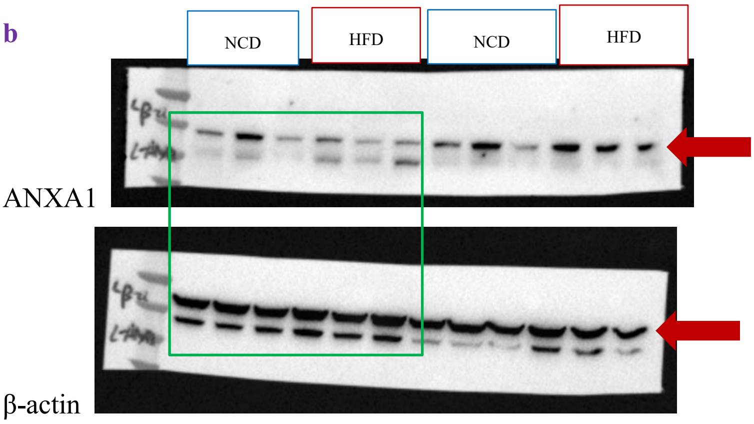

Fig. S2

a

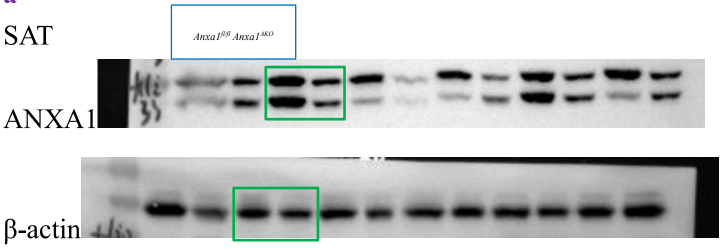

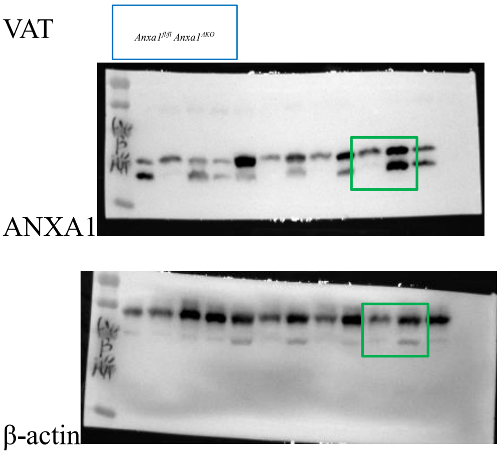

Liver

ANXA1

β-actin

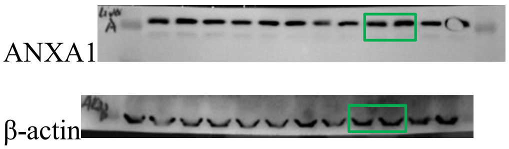

Heart

ANXA1

β-actin

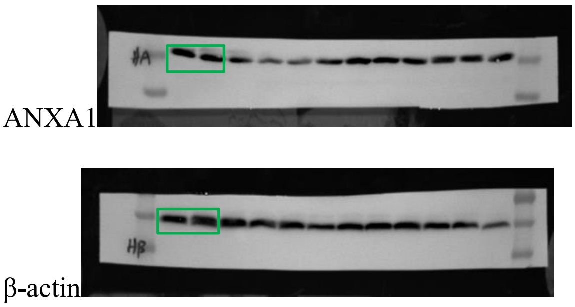

c

SAT

ADIPOQ

β-actin

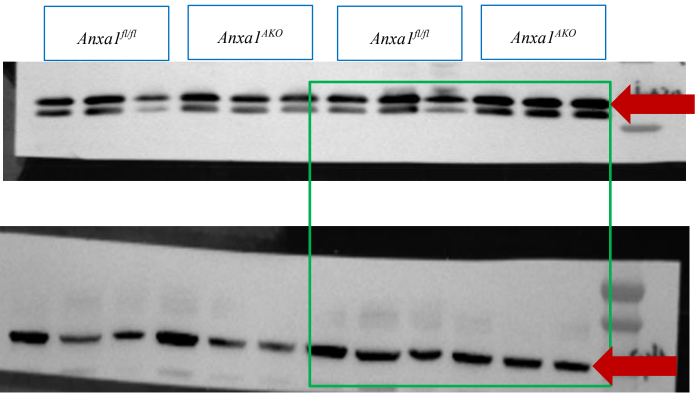

VAT

ADIPOQ

β-actin

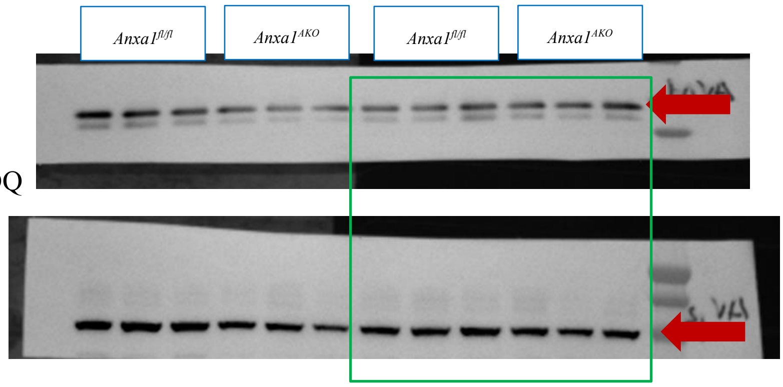

Fig. S3

a

SAT

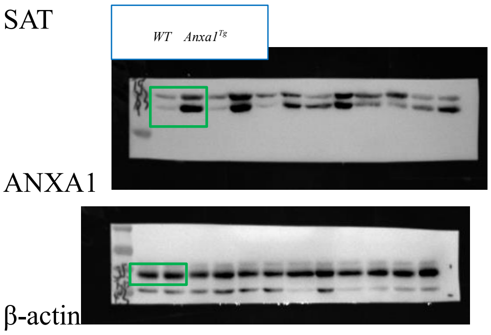

ANXA1

β-actin

Liver

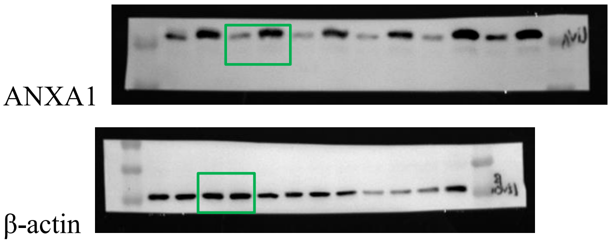

ANXA1

β-actin

Heart

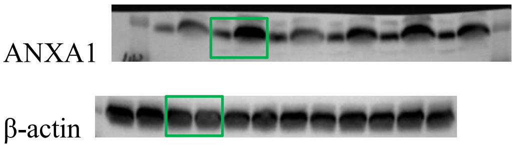

ANXA1

β-actin

c

SAT

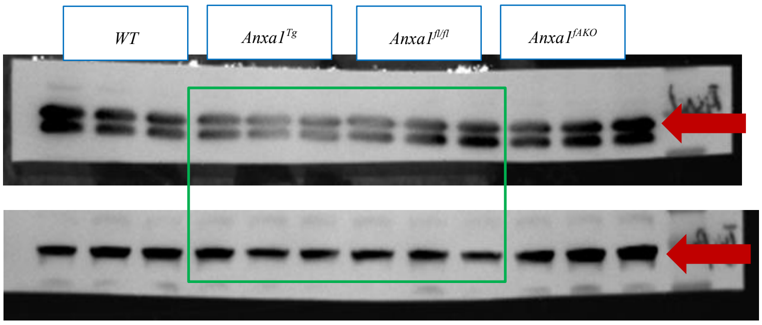

ADIPOQ

β-actin

VAT

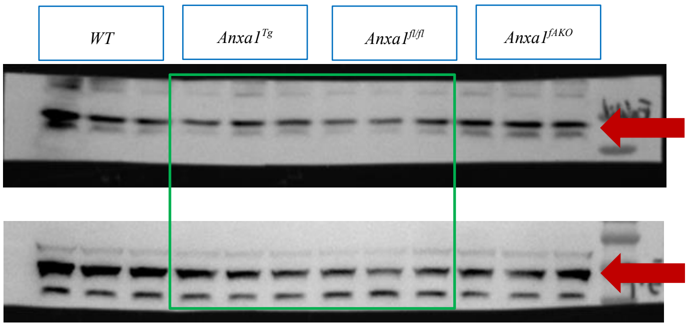

ADIPOQ

β-actin

Fig. S4

a

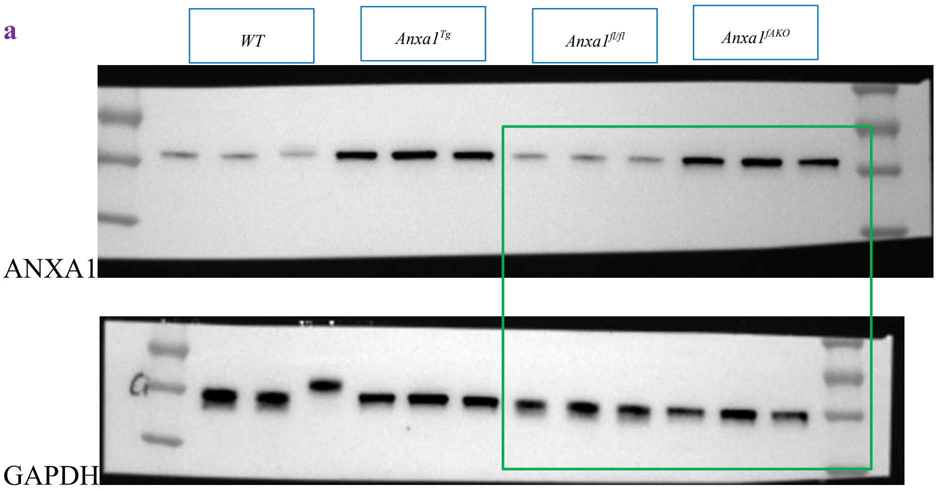

b

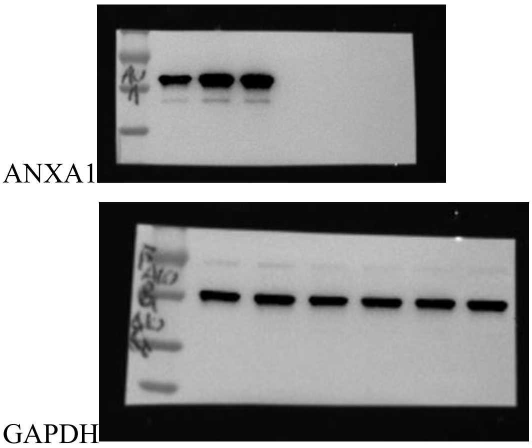

Fig. S5

a

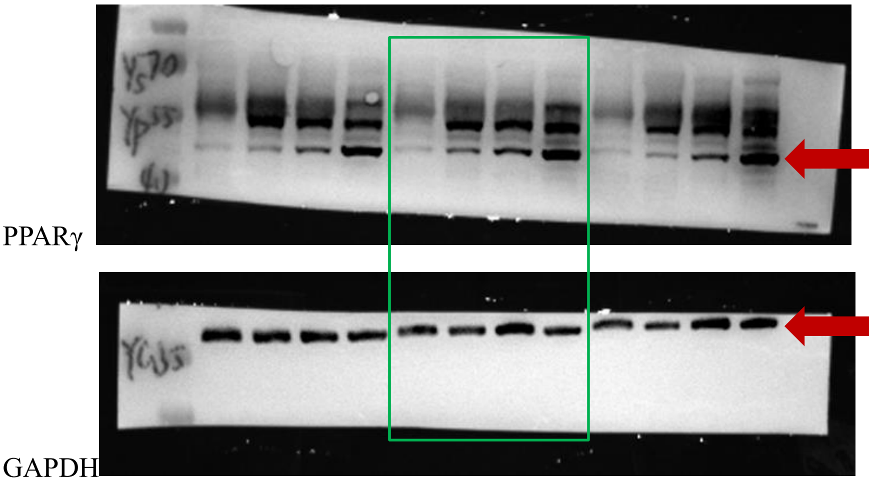

Fig. S6

c

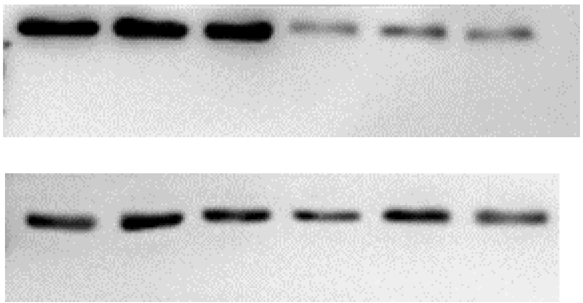

ANXA1

GAPDH

d

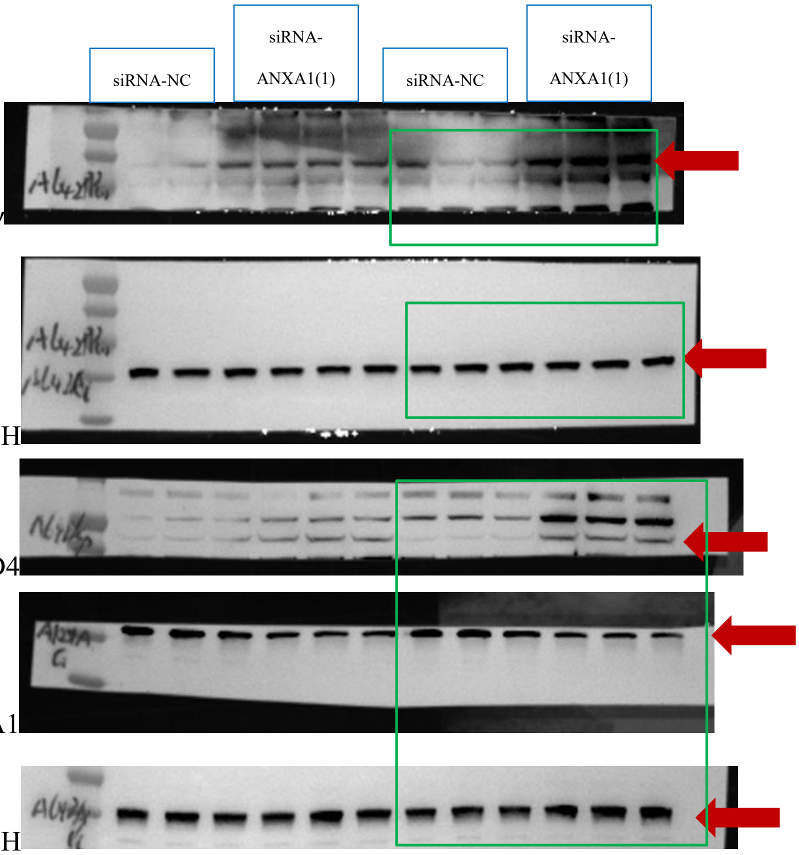

PPARγ

GAPDH

SMAD4

ANXA1

GAPDH

g

siRNA-NC(1)

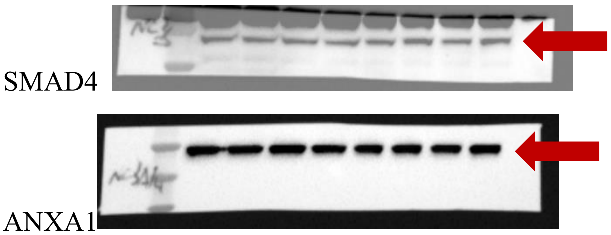

SMAD4

ANXA1

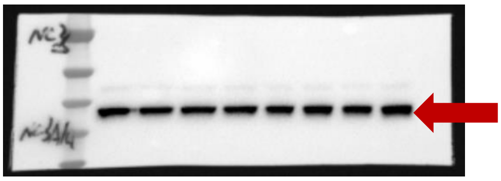

GAPDH

siRNA-NC(1)

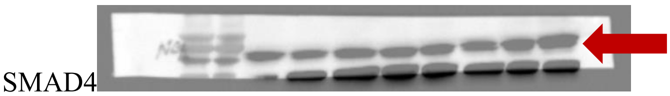

SMAD4

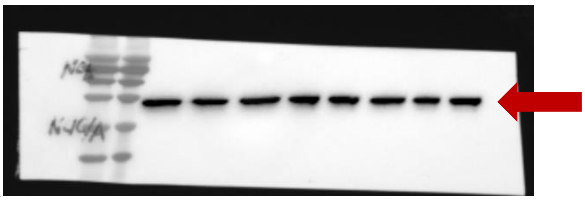

ANXA1

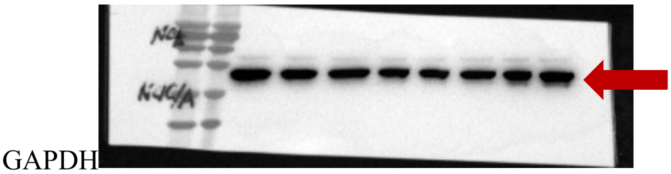

GAPDH

siRNA-NC(1)

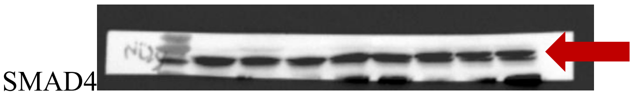

SMAD4

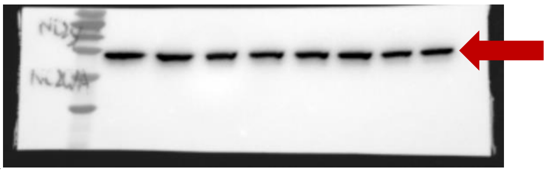

ANXA1

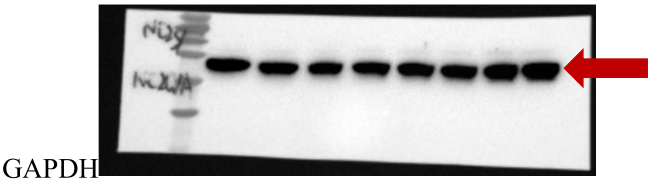

GAPDH

siRNA-ANXA1(1)

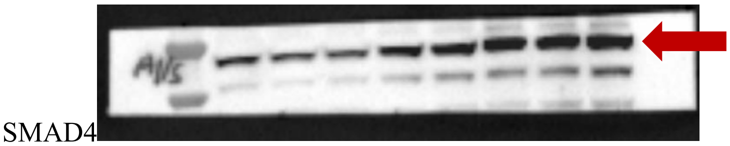

SMAD4

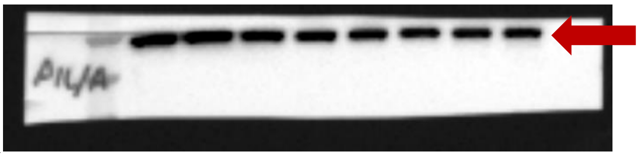

ANXA1

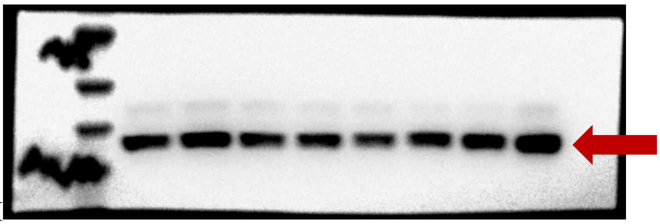

GAPDH

siRNA-ANXA1(1)

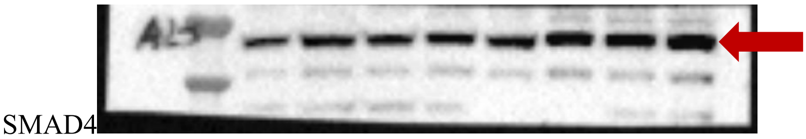

SMAD4

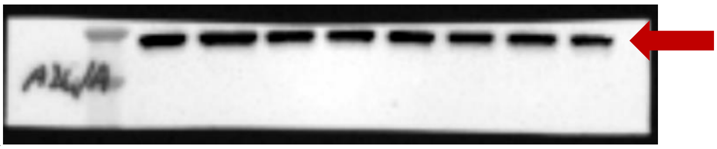

ANXA1

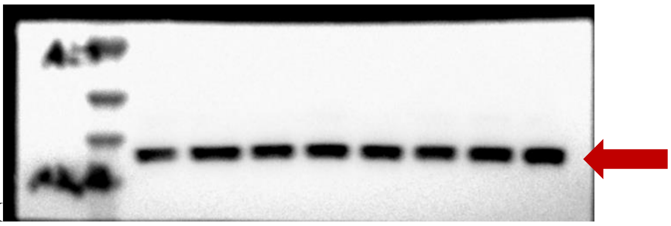

GAPDH

siRNA-ANXA1(1)

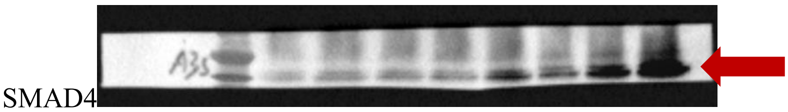

SMAD4

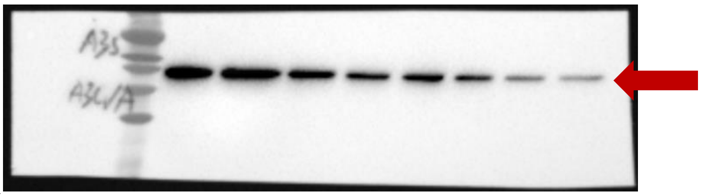

ANXA1

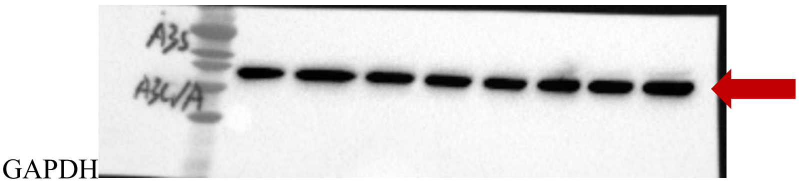

GAPDH

siRNA-ANXA1(1)

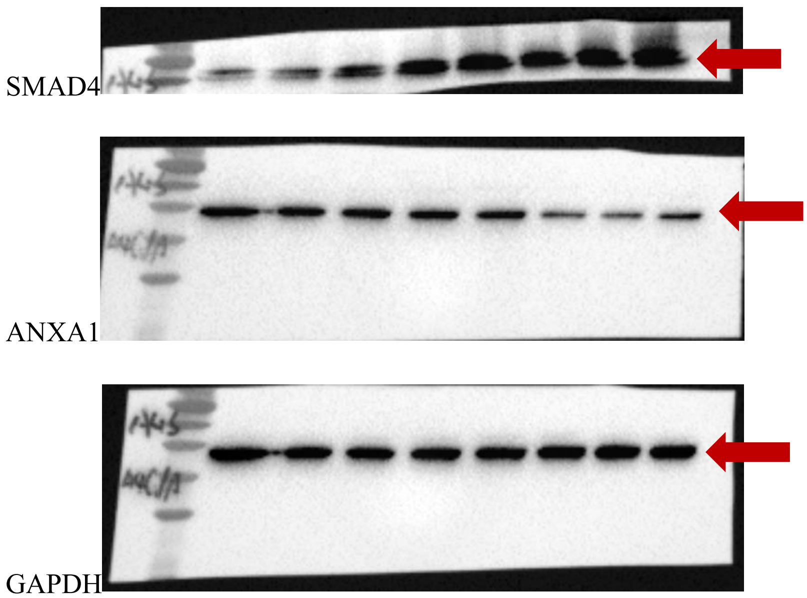
SMAD4
ANXA1
GAPDH

h

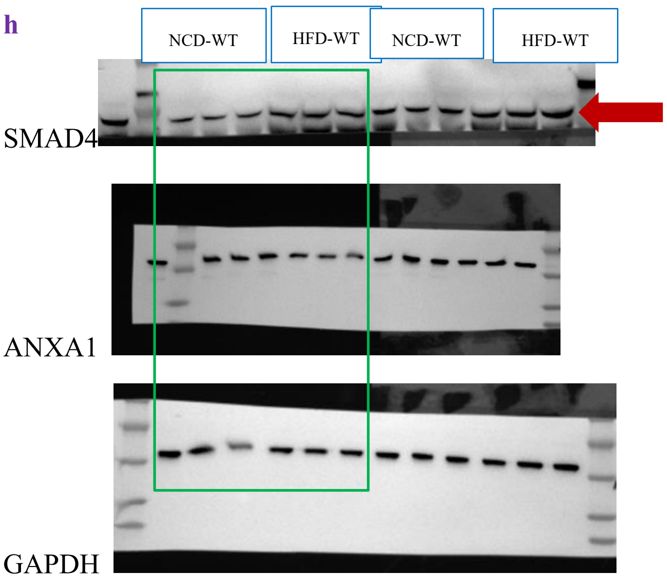
NCD-WT
HFD-WT
NCD-WT
HFD-WT
SMAD4
ANXA1
GAPDH

j

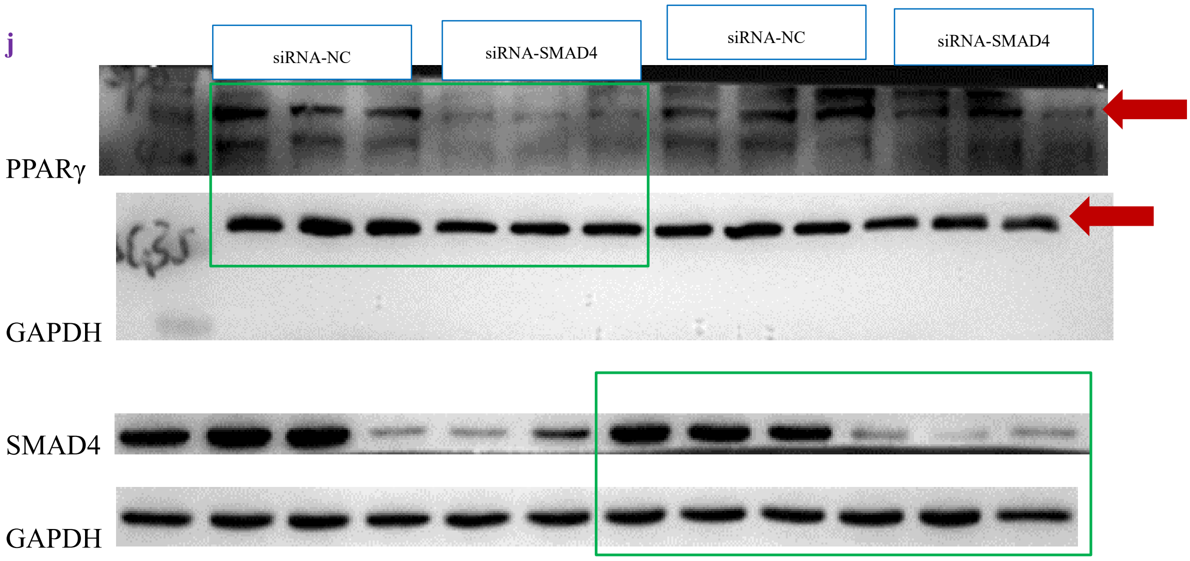
siRNA-NC
siRNA-SMAD4
siRNA-NC
siRNA-SMAD4
PPARγ
GAPDH
SMAD4
GAPDH

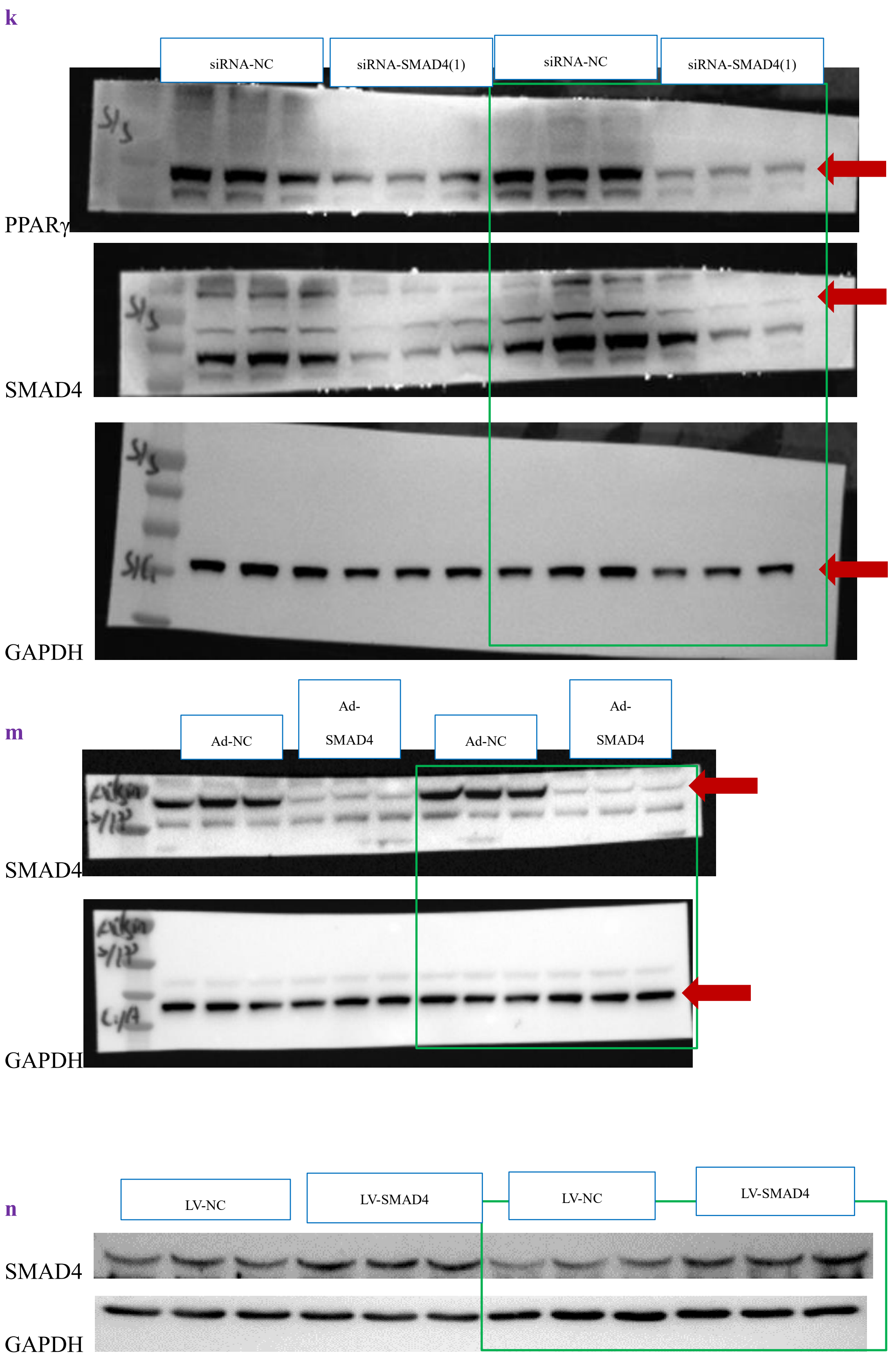
k
siRNA-NC
siRNA-SMAD4(1)
siRNA-NC
siRNA-SMAD4(1)
PPARγ
SMAD4
GAPDH
m
Ad-NC
Ad-SMAD4
Ad-NC
Ad-SMAD4
SMAD4
GAPDH
n
LV-NC
LV-SMAD4
LV-NC
LV-SMAD4
SMAD4
GAPDH

Fig. S7

b

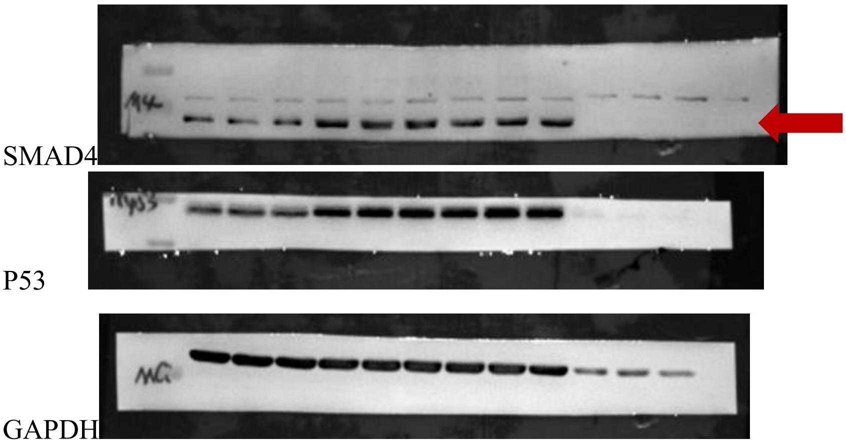
SMAD4
P53
GAPDH

c

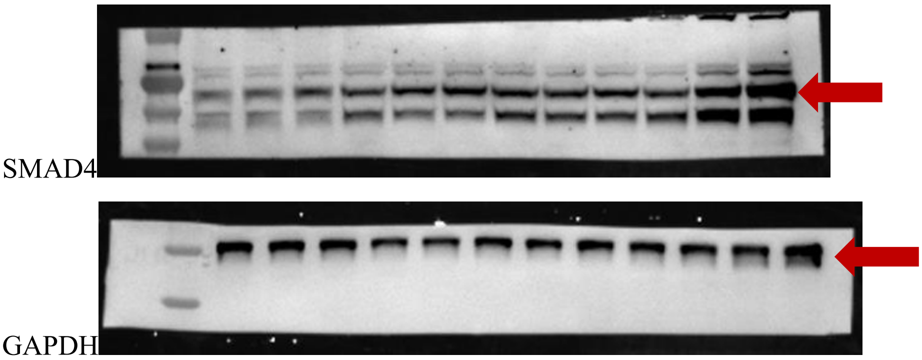
SMAD4
GAPDH

g

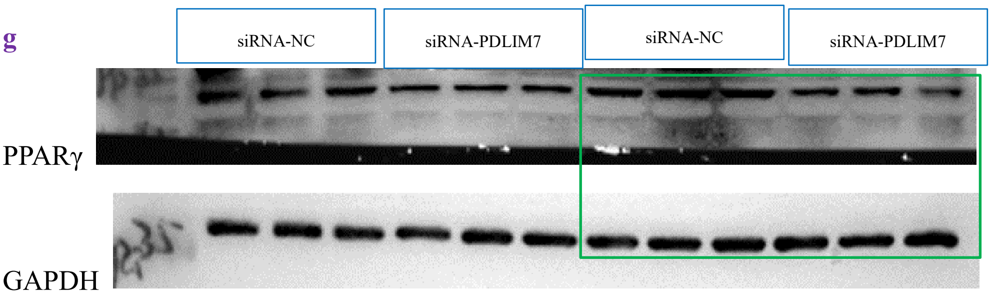
siRNA-NC
siRNA-PDLIM7
siRNA-NC
siRNA-PDLIM7
PPARγ
GAPDH

i

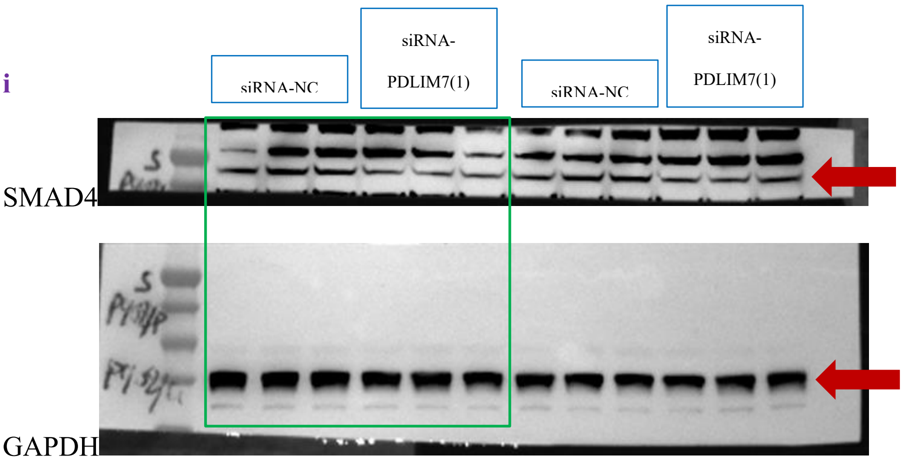
siRNA-NC
siRNA-PDLIM7(1)
siRNA-NC
siRNA-PDLIM7(1)
SMAD4
GAPDH

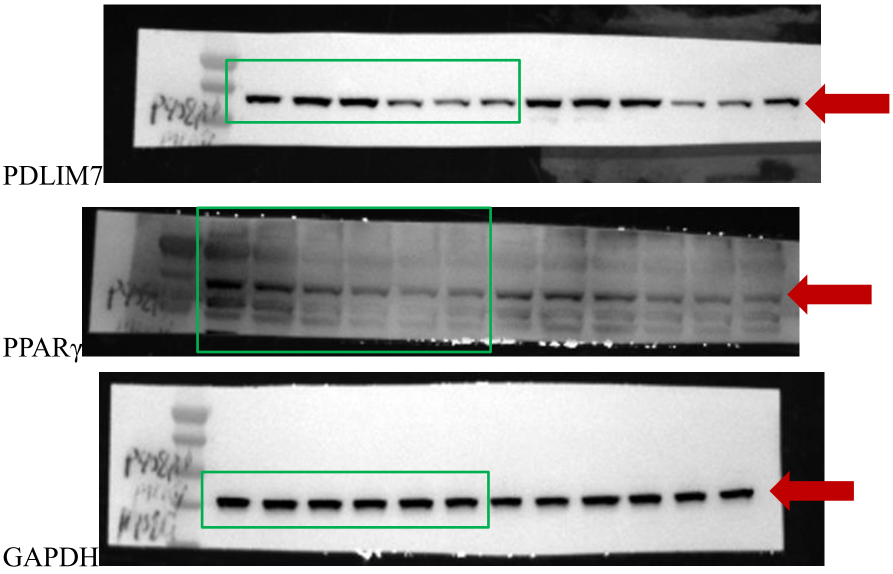

PDLIM7
PPARγ
GAPDH

k

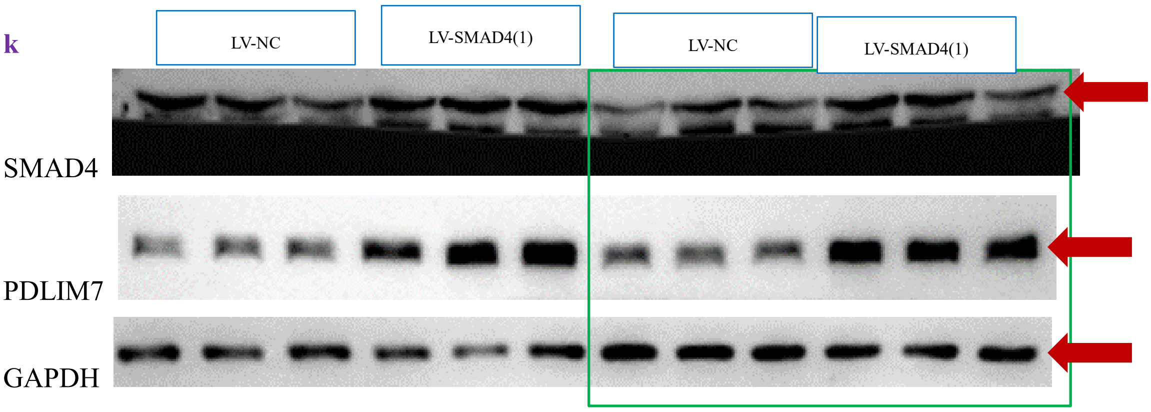

LV-NC
LV-SMAD4(1)
LV-NC
LV-SMAD4(1)
SMAD4
PDLIM7
GAPDH

Fig. S8

c

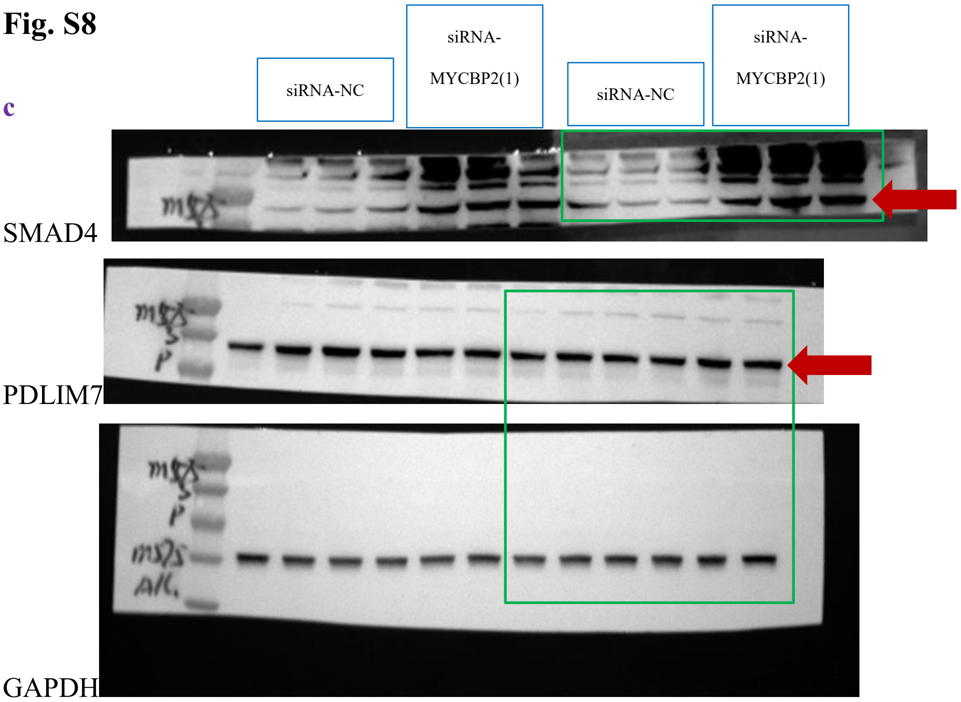

siRNA-NC
siRNA-MYCBP2(1)
siRNA-NC
siRNA-MYCBP2(1)
SMAD4
PDLIM7
GAPDH

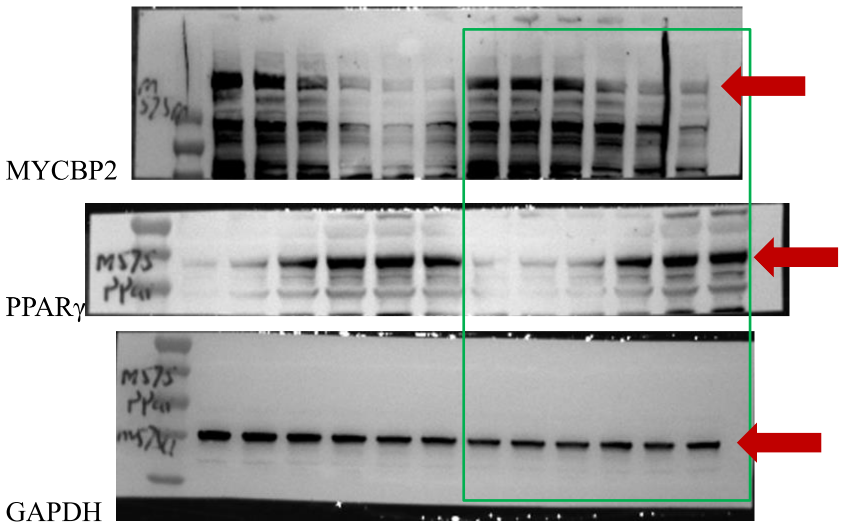
MYCBP2
PPARγ
GAPDH

g

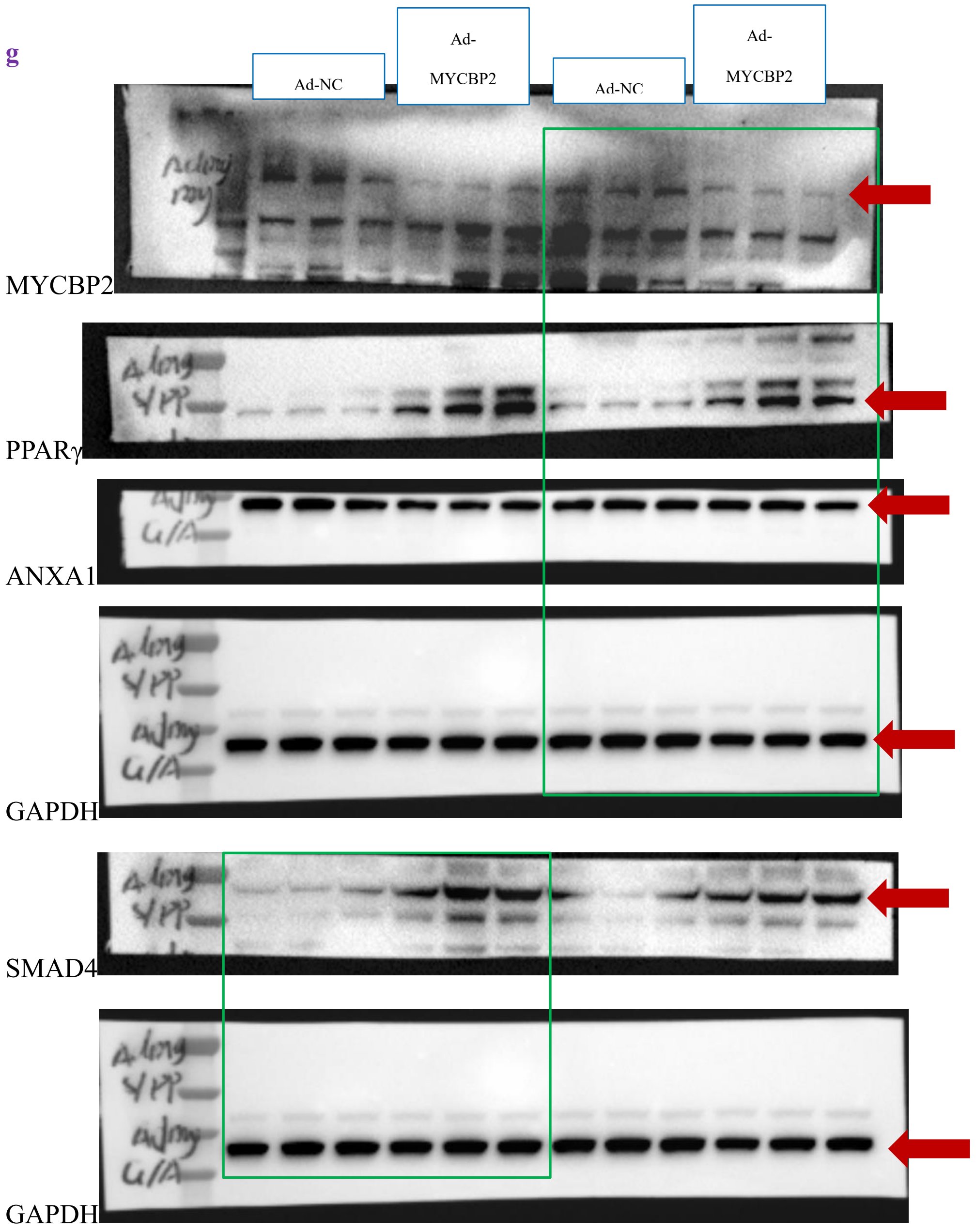
Ad-NC
Ad-MYCBP2
Ad-NC
Ad-MYCBP2
MYCBP2
PPARγ
ANXA1
GAPDH
SMAD4
GAPDH

h

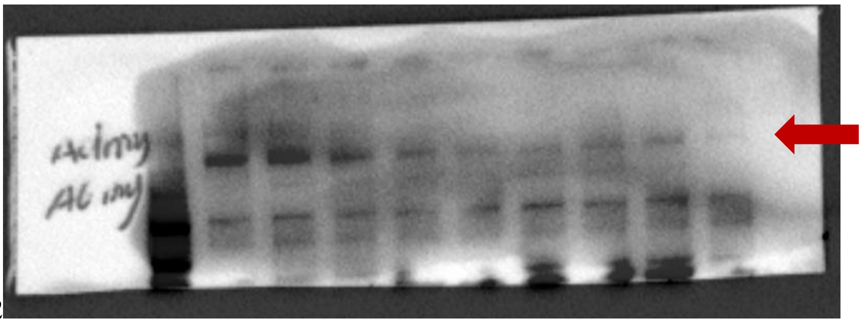

MYCBP2

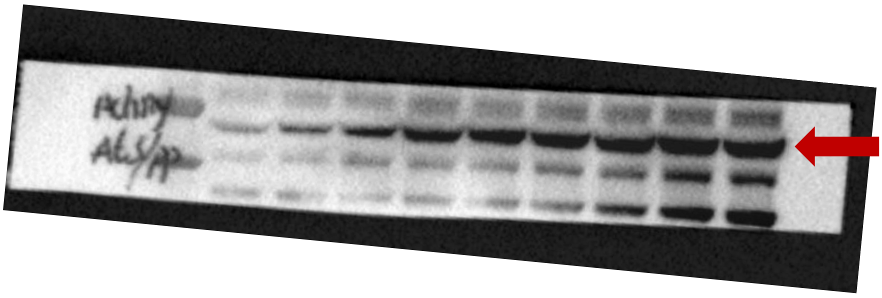

SMAD4

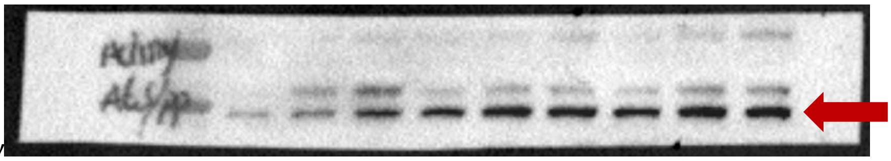

PPARγ

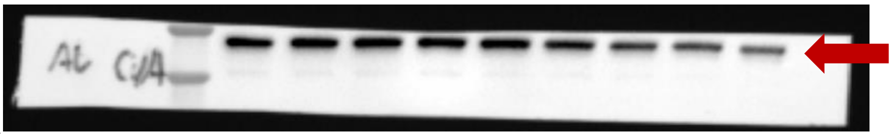

ANXA1

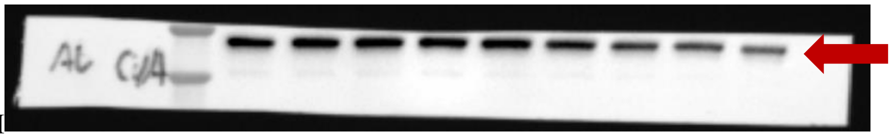

GAPDH

Fig. S9

a

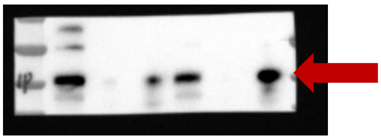

PDLIM7

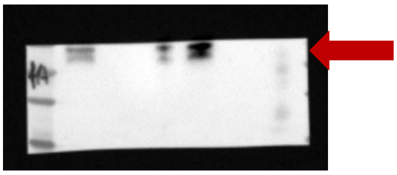

ANXA1

b

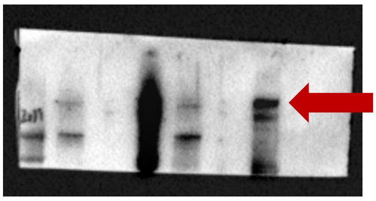

MYCBP2

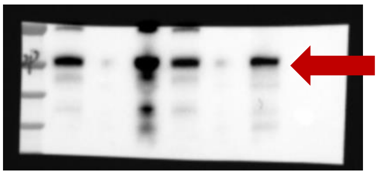

PDLIM7

c

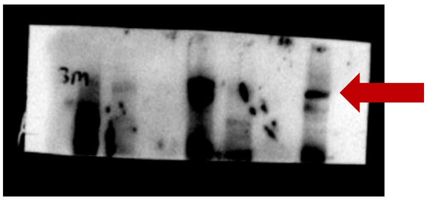

MYCBP2

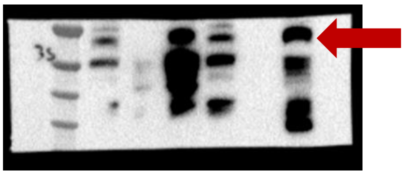

SMAD4

d

PDLIM7

ANXA1

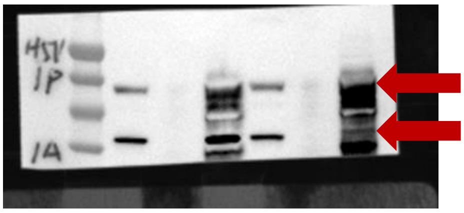

e

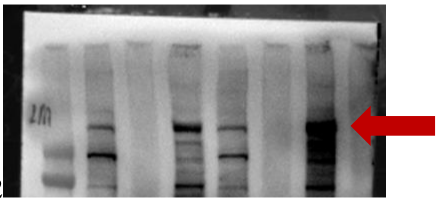

MYCBP2

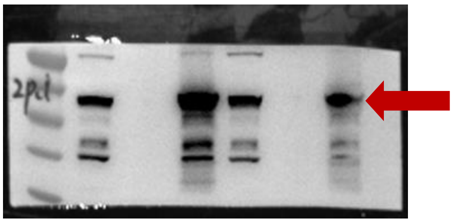

PDLIM7

f

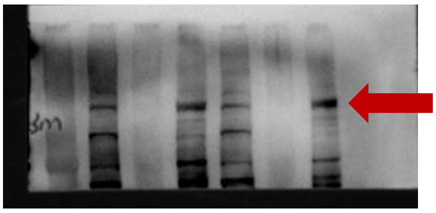

MYCBP2

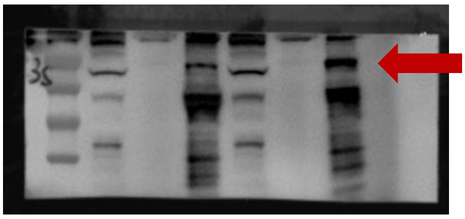

SMAD4

g

siRNA-NC	siRNA-ANXA1	siRNA-NC	siRNA-ANXA1(1)

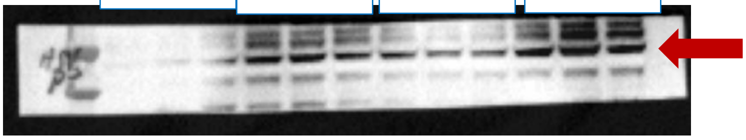

SMAD4

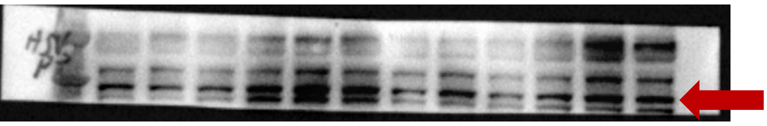

PPARγ

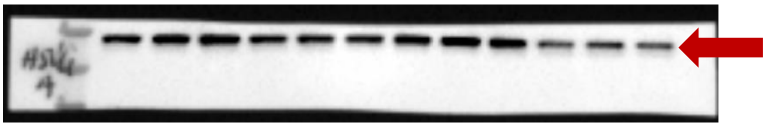

ANXA1

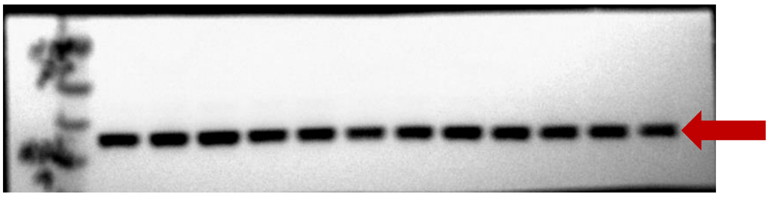

GAPDH
