# Supplementary material for: Annexin A1 binds PDZ and LIM domain 7 to inhibit adipogenesis and prevent obesity
Source: Signal Transduct Target Ther. 2024 Aug 23;9:218. doi: 10.1038/s41392-024-01930-0 (PMC11341699; doi:10.1038/s41392-024-01930-0)
Supplement: Supplementary file 2 — Supplementary figures and Supplementary tables [file 41392_2024_1930_MOESM2_ESM.docx]

Supplementary Materials for

Annexin A1 binds PDZ and LIM domain 7 to inhibit adipogenesis and prevent obesity

^#^Lu Fang, ^#^Changjie Liu, Zong-zhe Jiang, Mengxiao Wang, Kang Geng, Yangkai Xu, Yujie Zhu, Yiwen Fu, Jing Xue, Wenxin Shan, Qi Zhang, Jie Chen, Jiahong Chen, Mingming Zhao, Yuxuan Guo, K.W. Michael Siu, Y. Eugene Chen, *Yong Xu, *Donghui Liu, *Lemin Zheng

Correspondence to: zhengl@bjmu.edu.cn

**This PDF file includes:**

Supplementary Figure 1 to 10

Supplementary Table 1 to 4

**Other Supplementary Materials for this manuscript include the following:**

Unprocessed western blots

**
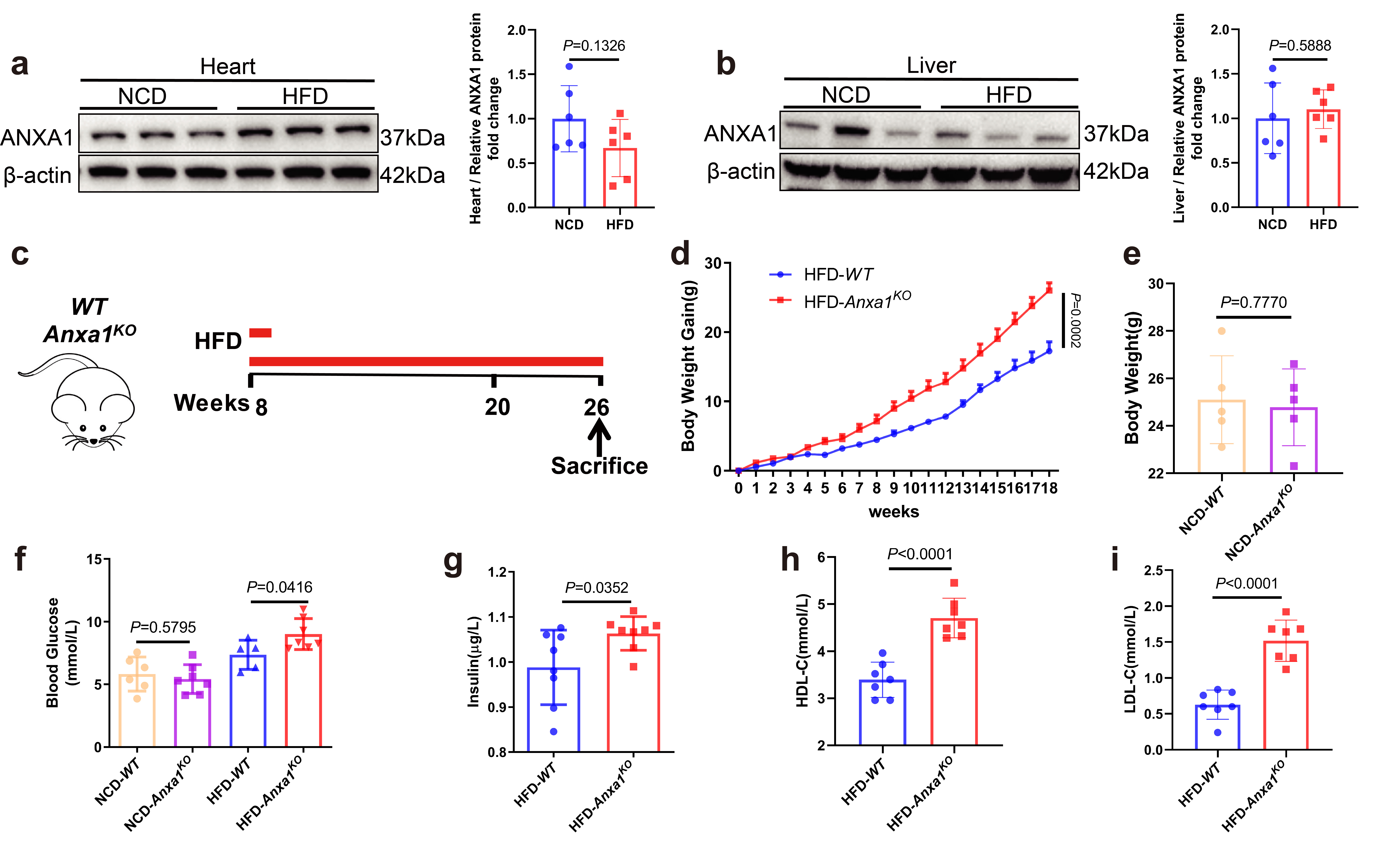
**

**Supplementary Figure.1. Knockdown of ANXA1 increases obesity.**

**a-b.** Representative western blotting and quantification of ANXA1 from heart and liver of *WT* mice fed with NCD or HFD for 12 weeks (n = 6 per group). Student's t-test was used for analysis.

**c.** *WT* and *Anxa1*^KO^ mice were fed with HFD for 12 or 18 weeks starting from 8 weeks of age, and then euthanized.

**d.** Average body weight gain ± s.e.m. of *WT* and *Anxa1*^KO^ mice fed with HFD for 18 weeks (n = 8 per group). One-way ANOVA and Dunn post hoc test were used for analysis.

**e.** Average body weight ± s.e.m. of *WT* and *Anxa1*^KO^ mice fed with NCD for 12 weeks (n = 5 per group). Student's t-test was used for analysis.

**f.** Blood glucose concentration in *WT* and *Anxa1*^KO^ mice fed with NCD or HFD for 12 weeks (n = 5-7 per group). Student's t-test was used for analysis.

**g-i.** Plasma insulin (**g**), high-density lipoprotein cholesterol (HDL-C) (**h**), and low-density lipoprotein cholesterol (LDL-C) (**i**) concentrations in *WT* and *Anxa1*^KO^ mice fed with HFD for 12 weeks (n = 7-8 per group). Student's t-test was used for analysis.


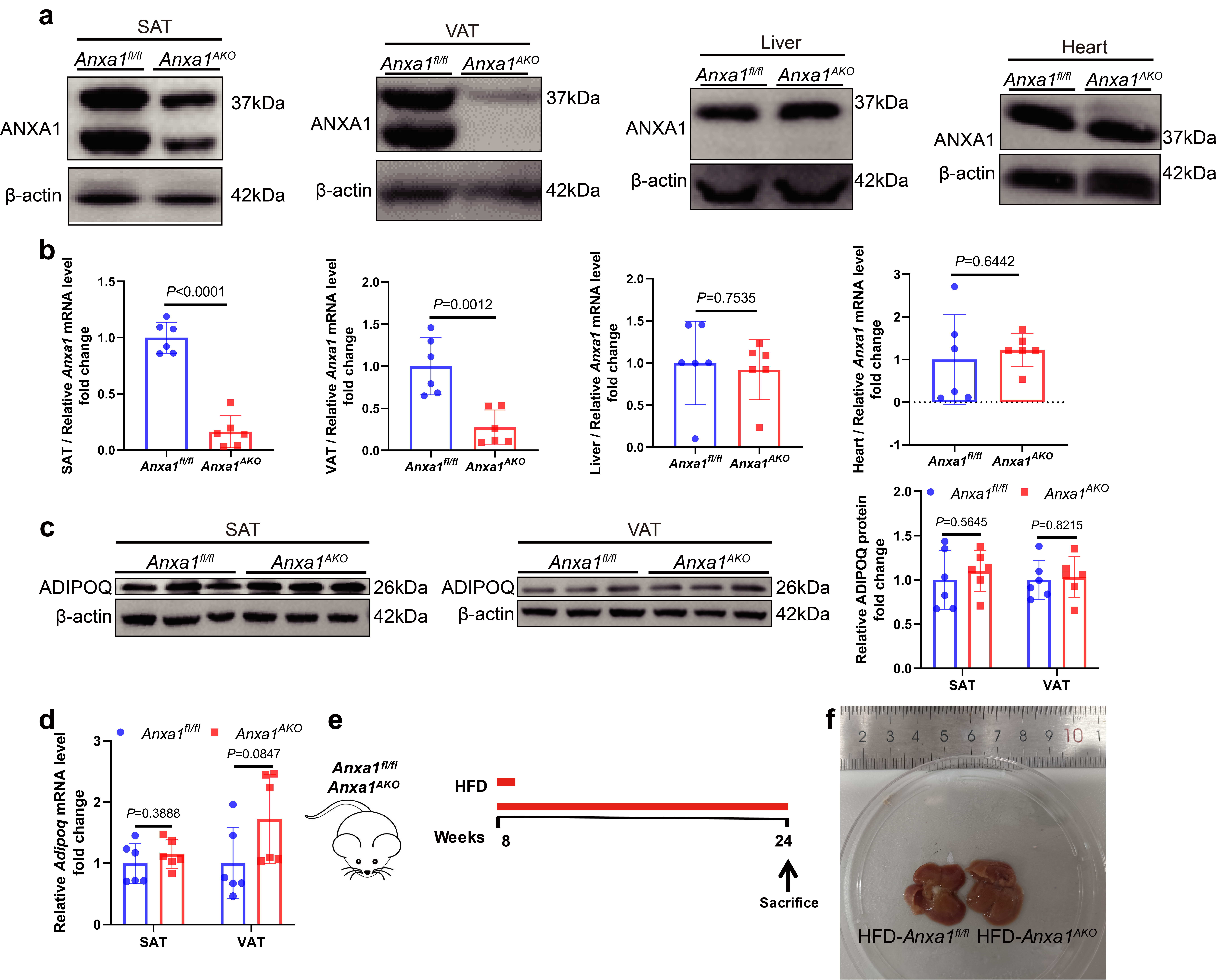


**Supplementary Figure.2. Adipose tissue ANXA1 deficiency does not cause exacerbation of fatty liver.**

**a.** Representative western blotting of ANXA1 in SAT, VAT, liver, and heart from 8-week-old *Anxa1^fl/fl^* and *Anxa1^AKO^* mice (n = 6 per group).

**b.** mRNA abundance of *Anxa1* in SAT, VAT, liver, and heart from 8-week-old *Anxa1^fl/fl^* and *Anxa1^AKO^* mice (n = 6 per group). Student's t-test was used for analysis.

**c.** Representative western blotting and quantification of ADIPOQ in SAT and VAT from 4-week-old *Anxa1^fl/fl^* and *Anxa1^AKO^* mice (n = 6 per group). One-way ANOVA and Dunn post hoc test were used for analysis.

**d.** mRNA abundance of Adipoq in SAT and VAT from 4-week-old *Anxa1^fl/fl^* and *Anxa1^AKO^* mice (n = 6 per group). One-way ANOVA and Dunn post hoc test were used for analysis.

**e.** *Anxa1^fl/fl^* and *Anxa1^AKO^* mice were fed with HFD for 16 weeks starting from 8 weeks of age, and then euthanized.

**f.** Representative photographs of the liver from *Anxa1^fl/fl^* and *Anxa1^AKO^* mice fed with HFD for 16 weeks (n = 5-8 per group).

**
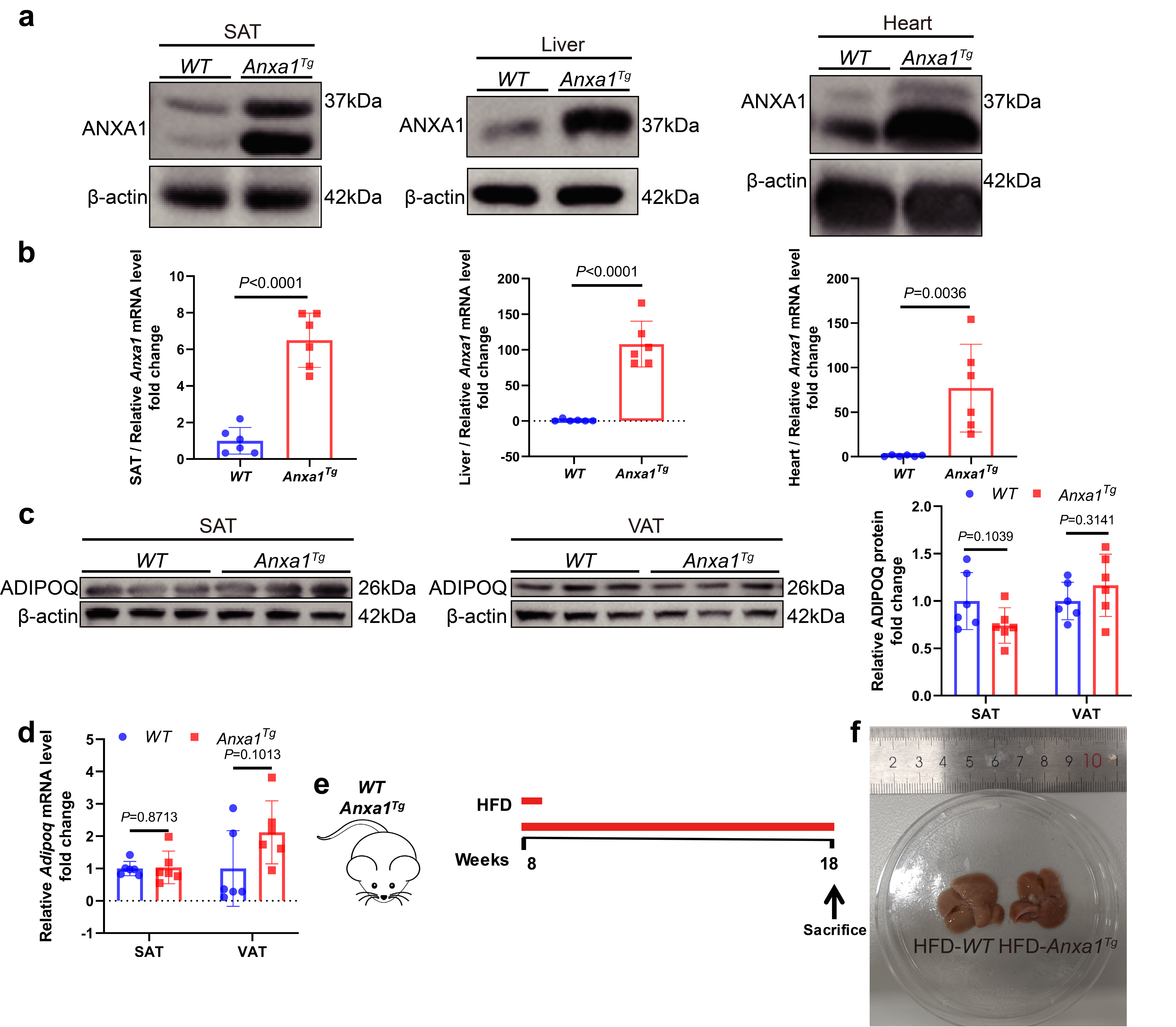
**

**Supplementary Figure.3. ANXA1 overexpression attenuates fatty liver.**

**a.** Representative western blotting of ANXA1 in SAT, liver, and heart from 8-week-old *WT* and *Anxa1^Tg^* mice.

**b.** mRNA abundance of *Anxa1* in SAT, liver, and heart from 8-week-old *WT* and *Anxa1^Tg^* mice (n = 6 per group). Student's t-test was used for analysis.

**c.** Representative western blotting and quantification of ADIPOQ in SAT and VAT from 4-week-old *WT* and *Anxa1^Tg^* mice (n = 6 per group). One-way ANOVA and Dunn post hoc test were used for analysis.

**d.** mRNA abundance of Adipoq in SAT and VAT from 4-week-old *WT* and *Anxa1^Tg^* mice (n = 6 per group). One-way ANOVA and Dunn post hoc test were used for analysis.

**e.** *WT* and *Anxa1^Tg^* mice were fed with HFD for 10 weeks starting from 8 weeks of age, and then euthanized.

**f.** Representative photographs of the liver from *WT* and *Anxa1^Tg^* mice fed with HFD for 10 weeks (n = 6-7 per group).


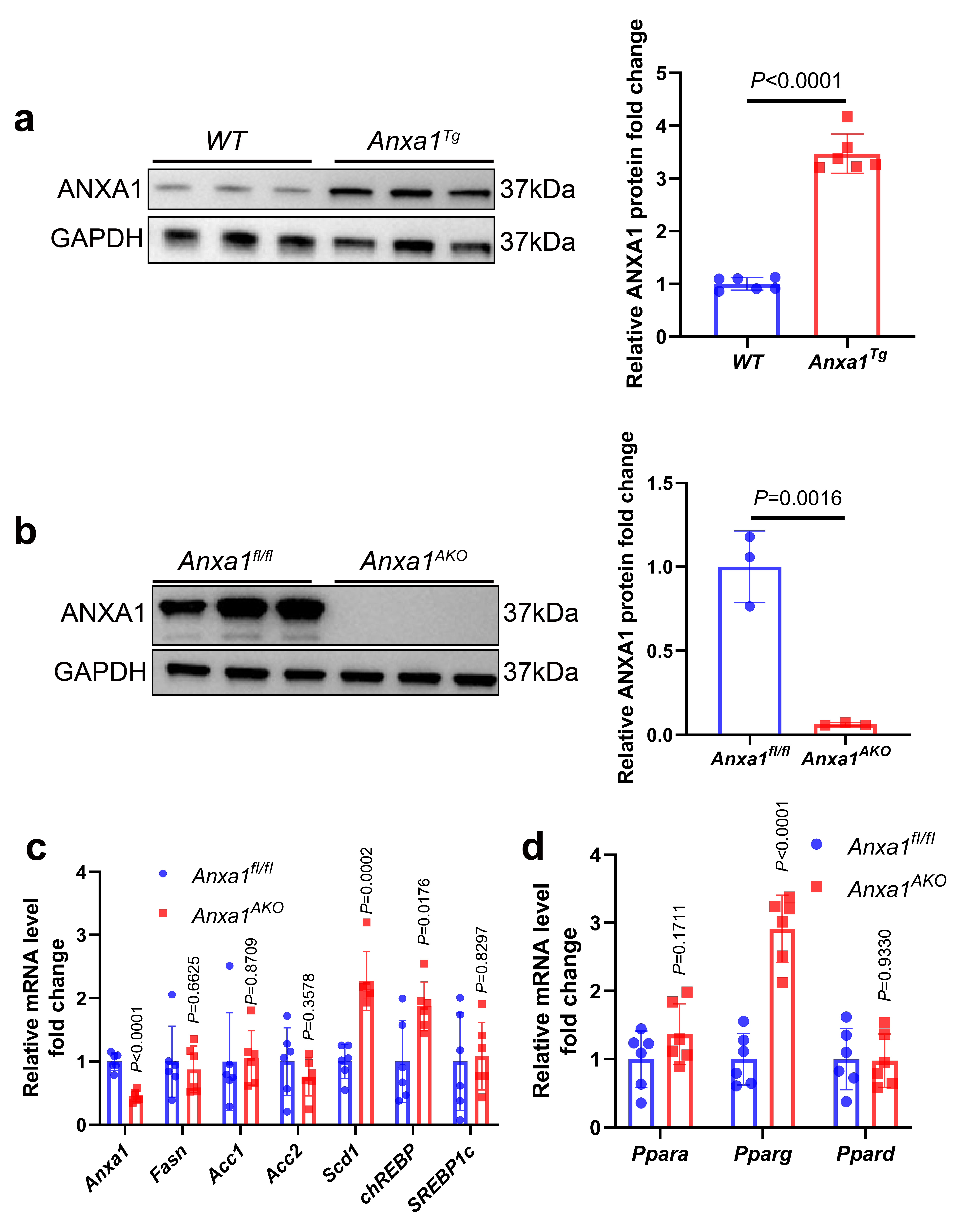


**Supplementary Figure.4.** **The mRNA levels of lipogenesis-related genes in SVFs of *Anxa1^AKO^* mice are increased.**

**a.** Representative western blotting and quantification of ANXA1 in SVFs from *WT* and *Anxa1^Tg^* mice (n = 6 per group). Student's t-test was used for analysis.

**b.** Representative western blotting and quantification of ANXA1 in SVFs from *Anxa1^fl/fl^* and *Anxa1^AKO^* mice (n = 3 per group). Student's t-test was used for analysis.

**c.** mRNA abundance of *Anxa1* and genes closely related to lipogenesis in SVFs from *Anxa1^fl/fl^* and *Anxa1^AKO^* mice (n = 6 per group). One-way ANOVA and Dunn post hoc test were used for analysis.

**d.** mRNA abundance of *Ppara*, *Pparg* and *Ppard* in SVFs from *Anxa1^fl/fl^* and *Anxa1^AKO^* mice (n = 6 per group). One-way ANOVA and Dunn post hoc test were used for analysis.


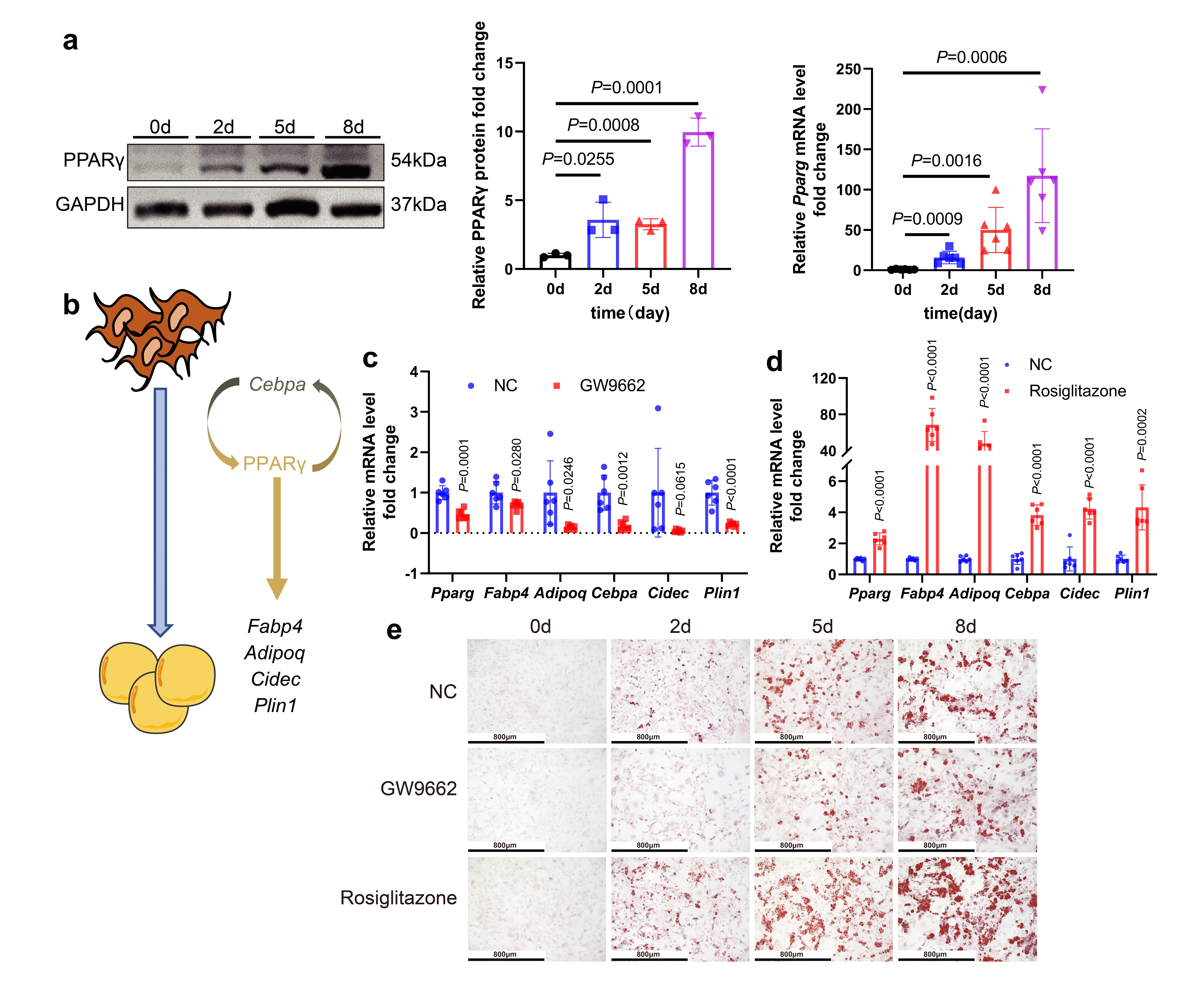


**Supplementary Figure.5. PPARγ promotes adipogenesis.**

**a.** Representative western blotting and quantification of PPARγ (n = 3 per group) and mRNA levels of *Pparg* (n = 6 per group) at different time points after adipogenic induction during the adipogenesis of SVFs. Student's t-test was used for analysis.

**b.** Schematic representation of PPARγ, *Cebpa*, *Fabp4*, *Adipoq*, *Cidec*, and *Plin1* in adipogenesis.

**c.** mRNA abundance of *Pparg* and genes closely related to adipogenesis in SVFs incubated with 20 µM GW9662 or DMSO (NC) for 48 hours (n = 6 per group). One-way ANOVA and Dunn post hoc test were used for analysis. The figure was modiﬁed from Servier Medical Art(http://smart.servier.com/), licensed under a Creative Common Attribution 3.0 Generic License. (https://creativecommons.org/licenses/by/3.0/).

**d.** mRNA abundance of *Pparg* and genes closely related to adipogenesis in SVFs incubated with 20 µM Rosiglitazone or DMSO for 48 hours (n = 6 per group). One-way ANOVA and Dunn post hoc test were used for analysis.

**e.** Oil Red O staining of SVFs at different time points after adipogenic induction with 20 µM GW9662, 20 µM Rosiglitazone, or DMSO (n = 6 per group). Scale bar: 800 µm.





**Supplementary Figure.6. Silence of ANXA1 increases SMAD4 and PPARγ levels.**

**a.** Flow cytometry analysis of SVFs co labeled with anti-CD105 (the marker of preadipocytes) antibody and anti-ANXA1 antibody.

**b.** Co-IF experiments demonstrate the co-expression of CD105 and ANXA1 on SVFs (preadipocytes). Scale bar: 800 µm, 80 µm.

**c.** Representative western blotting and quantification of ANXA1 (n = 3 per group), and mRNA levels of *Anxa1*(n = 6 per group) from SVFs transfected with ANXA1 siRNA or NC siRNA for 48 hours. Student's t-test was used for analysis.

**d.** Representative western blotting and quantification of ANXA1, SMAD4 and PPARγ from SVFs transfected with ANXA1 siRNA (1) or NC siRNA for 48 hours (n = 6 per group). One-way ANOVA and Dunn post hoc test were used for analysis.

**e.** mRNA abundance of *Anxa1*, *Pparg* and genes closely related to adipogenesis in SVFs transfected with ANXA1 siRNA (1) or NC siRNA for 48 hours (n = 6 per group). One-way ANOVA and Dunn post hoc test were used for analysis.

**f.** mRNA abundance of *IL-6*, *Ccl2*, and *Cxcl10* in SVFs transfected with ANXA1 siRNA or NC siRNA for 48 hours (n = 6 per group). One-way ANOVA and Dunn post hoc test were used for analysis.

**g.** Representative western blotting of ANXA1 and SMAD4 from SVFs at different time points after transfection with NC siRNA or ANXA1 siRNA (1) (n = 3-4 per group).

**h.** Representative western blotting and quantification of ANXA1 and SMAD4 from SVFs of *WT* mice fed with NCD or HFD for 12 weeks (n = 6 per group). One-way ANOVA and Dunn post hoc test were used for analysis.

**i.** Average expression of *ANXA1* in ASPC by high or low BMI human for genes in interactions identified by CellphoneDB (human expression).

**j.** Representative western blotting and quantification of PPARγ and SMAD4 from SVFs transfected with SMAD4 siRNA or NC siRNA for 48 hours (n = 6 per group). One-way ANOVA and Dunn post hoc test were used for analysis.

**k.** Representative western blotting and quantification of PPARγ and SMAD4 from SVFs transfected with SMAD4 siRNA (1) or NC siRNA for 48 hours (n = 6 per group). One-way ANOVA and Dunn post hoc test were used for analysis.

**l.** mRNA abundance of *Smad4*, *Pparg* and genes closely related to adipogenesis in SVFs transfected with SMAD4 siRNA (1) or NC siRNA for 48 hours (n = 6 per group). One-way ANOVA and Dunn post hoc test were used for analysis.

**m.** Representative western blotting and quantification of SMAD4, and mRNA levels of *Smad4* from SVFs transfected with SMAD4 adenovirus or NC adenovirus for 72 hours (n = 6 per group). Student's t-test was used for analysis.

**n.** Representative western blotting and quantification of SMAD4, and mRNA levels of *Smad4* from SVFs transfected with SMAD4 lentivirus or NC lentivirus for 96 hours (n = 6 per group). Student's t-test was used for analysis.





**Supplementary Figure.7. PDLIM7 promotes SMAD4 protein accumulation and adipogenesis.**

**a.** mRNA abundance of *Smad4* in SVFs transfected with ANXA1 siRNA (1) or NC siRNA for 48 hours (n = 6 per group). Student's t-test was used for analysis.

**b.** Representative western blotting and quantification of SMAD4 and P53 at different time points after treatment with 10 µM MG132 in SVFs (n = 3 per group). Student's t-test was used for analysis.

**c.** Representative western blotting and quantification of SMAD4 from *Anxa1^fl/fl^*-SVFs, *Anxa1^AKO^*-SVFs, and *Anxa1^fl/fl^*-SVFs and *Anxa1^AKO^*-SVFs incubated with 10 µM MG132 for 6 hours (n = 6 per group). Student's t-test was used for analysis.

**d.** The Venn diagram shows that there are 55 proteins that specifically interact with ANXA1.

**e.** The STRING database indicates that PDLIM7 is associated with SMAD4 protein (https://cn.string-db.org/).

**f.** Co-IF experiments demonstrate the interaction between ANXA1 and PDLIM7. Scale bar: 800 µm, 160 µm.

**g.** Representative western blotting of PPARγ from SVFs transfected with PDLIM7 siRNA or NC siRNA for 48 hours (n = 6 per group). Student's t-test was used for analysis.

**h.** mRNA abundance of *Pdlim7*, *Anxa1*, and *Smad4* in SVFs transfected with PDLIM7 siRNA or NC siRNA for 48 hours (n = 6 per group). One-way ANOVA and Dunn post hoc test were used for analysis.

**i.** Representative western blotting of SMAD4, PDLIM7 and PPARγ from SVFs transfected with PDLIM7 siRNA (1) or NC siRNA for 48 hours (n = 6 per group). One-way ANOVA and Dunn post hoc test were used for analysis.

**j.** mRNA abundance of *Pdlim7*, *Anxa1*, *Smad4*, *Pparg* and genes closely related to adipogenesis in SVFs transfected with PDLIM7 siRNA (1) or NC siRNA for 48 hours (n = 6 per group). One-way ANOVA and Dunn post hoc test were used for analysis.

**k.** Representative western blotting and quantification of PDLIM7 and SMAD4 in SVFs transfected with PDLIM7 lentivirus or NC lentivirus for 96 hours (n = 6 per group). One-way ANOVA and Dunn post hoc test were used for analysis.

**l.** mRNA abundance of *Pdlim7*, *Pparg* and genes closely related to adipogenesis in SVFs transfected with PDLIM7 lentivirus or NC lentivirus for 96 hours (n = 6 per group). One-way ANOVA and Dunn post hoc test were used for analysis.





**Supplementary Figure.8. MYCBP2 interacts with both PDLIM7 and SMAD4 and** **downregulates SMAD4 and PPARγ levels.**

**a.** The Venn diagram shows that there are 55 proteins that specifically interact with PDLIM7.

**b.** Co-IF experiments demonstrate the interaction between PDLIM7 and MYCBP2. Scale bar: 800 µm, 160 µm.

**c.** Representative western blotting and quantification of MYCBP2, PPARγ, SMAD4 and PDLIM7 from SVFs transfected with MYCBP2 siRNA (1) or NC siRNA for 48 hours (n = 6 per group). One-way ANOVA and Dunn post hoc test were used for analysis.

**d.** mRNA abundance of *Anxa1*, *Pdlim7*, *Mycbp2* and *Smad4* in SVFs transfected with MYCBP2 siRNA (1) or NC siRNA for 48 hours (n = 6 per group). One-way ANOVA and Dunn post hoc test were used for analysis.

**e.** mRNA abundance of *Pparg* and genes closely related to adipogenesis in SVFs transfected with MYCBP2 siRNA (1) or NC siRNA for 48 hours (n = 6 per group). One-way ANOVA and Dunn post hoc test were used for analysis.

**f.** Co-IF experiments demonstrate the interaction between MYCBP2 and SMAD4. Scale bar: 800 µm, 160 µm.

**g.** Representative western blotting and quantification of MYCBP2, SMAD4, PPARγ and ANXA1 from SVFs transfected with MYCBP2 adenovirus or NC adenovirus for 72 hours (n = 6 per group). One-way ANOVA and Dunn post hoc test were used for analysis.

**h.** Representative western blotting and quantification of MYCBP2, SMAD4, PPARγ and ANXA1 from SVFs incubation with MYCBP2 adenovirus or NC adenovirus for 96 hours and transfected with ANXA1 siRNA (1) or NC siRNA for 48 hours (n = 3 per group). One-way ANOVA and Dunn post hoc test were used for analysis.

**i.** mRNA abundance of *Anxa1*, *Pdlim7*, *Mycbp2*, *Smad4*, *Pparg* and genes closely related to adipogenesis from SVFs incubation with MYCBP2 adenovirus or NC adenovirus for 96 hours and transfected with ANXA1 siRNA, ANXA1 siRNA (1) or NC siRNA for 48 hours (n = 3-6 per group). Compared to Ad-MYCBP2 siRNA-NC, one-way ANOVA and Dunn post hoc test were used for analysis.


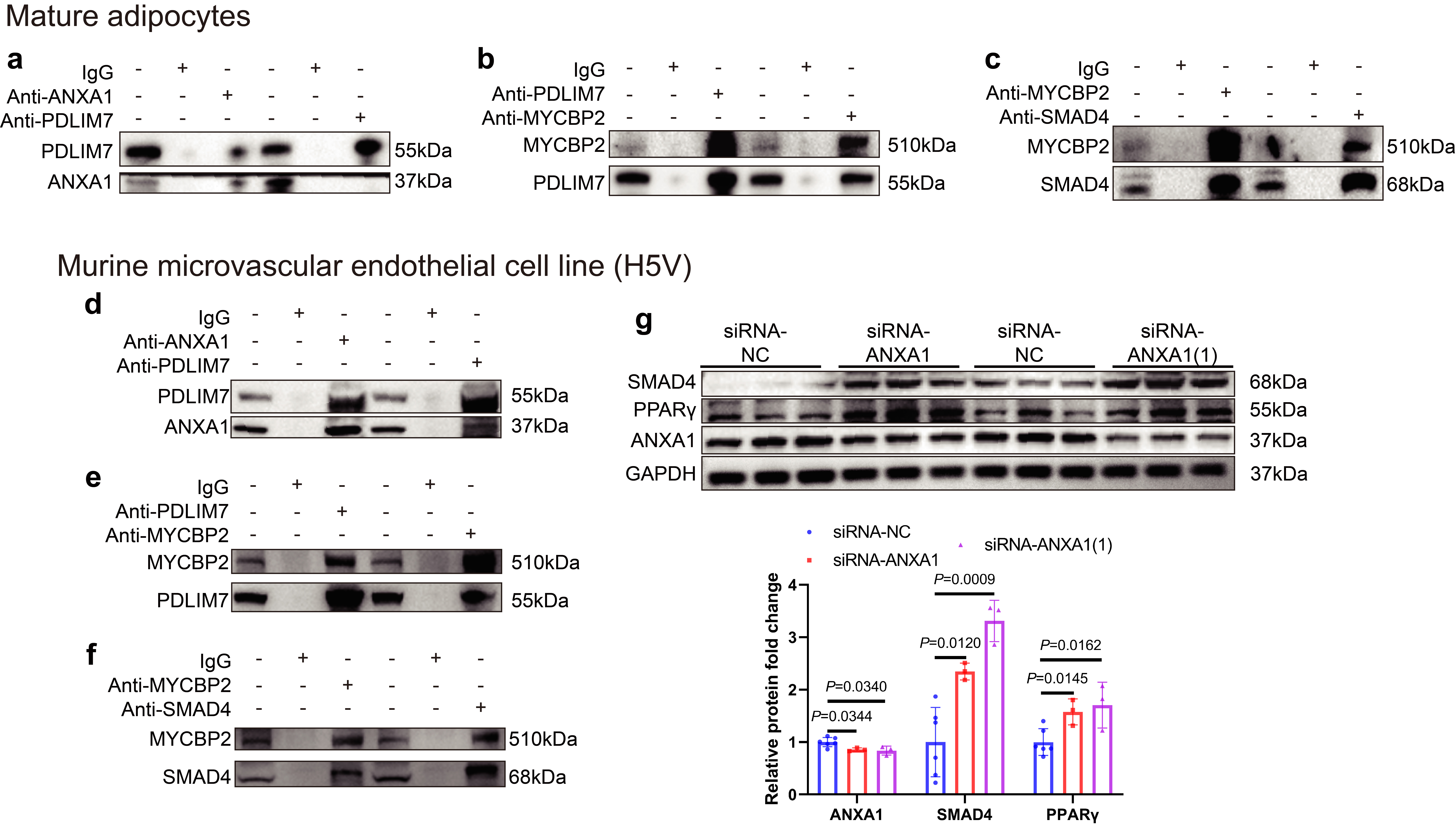


**Supplementary Figure.9. The ANXA1-PDLIM7-MYCBP2-SMAD4 axis still exists in mature adipocytes and H5V cells.**

**a.** Co-immunoprecipitation assay of ANXA1 and PDLIM7 in mature adipocytes.

**b.** Co-immunoprecipitation assay of PDLIM7 and MYCBP2 in mature adipocytes.

**c.** Co-immunoprecipitation assay of MYCBP2 and SMAD4 in mature adipocytes.

**d.** Co-immunoprecipitation assay of ANXA1 and PDLIM7 in H5V cells.

**e.** Co-immunoprecipitation assay of PDLIM7 and MYCBP2 in H5V cells.

**f.** Co-immunoprecipitation assay of MYCBP2 and SMAD4 in H5V cells.

**g.** Representative western blotting and quantification of ANXA1, SMAD4 and PPARγ from H5V cells transfected with ANXA1 siRNA, ANXA1 siRNA (1) or NC siRNA for 48 hours (n = 3-6 per group). One-way ANOVA and Dunn post hoc test were used for analysis.





**Supplementary Figure.10. GW9662 prevents *Anxa1* AKO-induced obesity in mice.**

**a-j.** Eight-week-old *Anxa1^AKO^* mice were injected intraperitoneally with 1 mg/kg of GW9662 (dissolved in PBS solution containing 10% DMSO) or an equivalent amount of PBS solution containing 10% DMSO for 16 weeks and were fed with HFD throughout.

**a.** Average body weight ± s.e.m. of the two groups of mice (n = 5-8 per group). One-way ANOVA and Dunn post hoc test were used for analysis.

**b.** Representative photographs of the two groups of mice (n = 5-8 per group).

**c.** Representative photographs of the liver from the two groups of mice (n = 5-8 per group).

**d.** Liver weight of the two groups of mice (n = 5-8 per group). Student's t-test was used for analysis.

**e.** Representative photographs of subcutaneous fat (upper left), perirenal fat (upper right), and gonadal fat (lower) from the two groups of mice (n = 5-8 per group).

**f.** Subcutaneous fat, perirenal fat, and gonadal fat weight of the two groups of mice (n = 5-8 per group). One-way ANOVA and Dunn post hoc test were used for analysis.

**g.** Hematoxylin & eosin-stained SAT sections showing adipocyte size in the two groups of mice. Scale bar: 800 µm. Student's t-test was used for analysis.

**h.** Results of glucose tolerance test (GTT)(left) were quantified as area under the curve (AUC)(right) for the two groups of mice (n = 5-8 per group). One-way ANOVA and Dunn post hoc test were used for analysis.

**i.** Results of insulin tolerance test (ITT)(left) were quantified as AUC(right) for the two groups of mice (n = 5-8 per group). One-way ANOVA and Dunn post hoc test were used for analysis.

**j.** Plasma insulin concentration in the mice (n = 5-8 per group). Student's t-test was used for analysis.

Supplementary Table 1

| Clinical characteristics of Lean and Obese individuals analysed for ANXA1 expression, related to Figure 1c-d. | | | | | |
| --- | --- | --- | --- | --- | --- |
|  | gender | Age (years) | Height (cm) | Body weight (kg) | BMI |
| Lean-1 | female | 48 | 158 | 54.5 | 21.8 |
| Lean-2 | male | 57 | 168 | 58 | 20.5 |
| Lean-3 | female | 21 | 164 | 49 | 18.2 |
| Lean-4 | male | 19 | 163 | 56 | 21 |
| Lean-5 | male | 21 | 181 | 62 | 18.9 |
|  |  |  |  |  |  |
| obese-1 | female | 54 | 142 | 69 | 34.2 |
| obese-2 | male | 21 | 165 | 75 | 27.5 |
| obese-3 | female | 47 | 162 | 82 | 31.2 |
| obese-4 | female | 35 | 163 | 82 | 30.8 |
| obese-5 | male | 21 | 174 | 82 | 27 |

Supplementary Table 2

| Plasmid name | sequence(5'-3') | Cloning Vector |
| --- | --- | --- |
| LV-SMAD4 | ATTCTGGCCGTTTTTGGCTTTTTTGTTAGACGAAGCTTGGGCTGCAGGTCGACTCTAGAGGATCCCGCCACCATGGACAATATGTCTATAACAAATACACCAACAAGTAACGATGCCTGTCTGAGCATTGTACATAGTTTGATGTGTCATAGACAAGGTGGGGAAAGTGAAACCTTTGCAAAAAGAGCAATTGAGAGTTTGGTAAAGAAGCTGAAAGAGAAAAAAGATGAATTGGATTCTTTAATAACAGCTATAACTACAAATGGAGCTCATCCTAGCAAGTGTGTCACCATACAGAGAACATTGGATGGACGACTTCAGGTGGCTGGTCGGAAAGGATTTCCTCATGTGATCTATGCCCGTCTGTGGAGGTGGCCTGATCTACACAAGAATGAACTAAAGCATGTTAAATATTGTCAGTATGCGTTTGACTTAAAATGTGACAGTGTCTGTGTGAATCCATATCACTATGAGCGGGTTGTCTCACCTGGAATTGATCTCTCAGGATTAACACTGCAGAGTAATGCTCCAAGTATGTTAGTGAAGGATGAGTACGTTCACGACTTTGAAGGACAGCCGTCCTTACCCACTGAAGGACATTCGATTCAAACCATCCAACACCCGCCAAGTAATCGCGCATCAACGGAGACGTACAGCGCCCCGGCTCTGTTAGCCCCGGCAGAGTCTAACGCCACCAGCACCACCAACTTCCCCAACATTCCTGTGGCTTCCACAAGTCAGCCGGCCAGTATTCTGGCGGGCAGCCATAGTGAAGGACTGTTGCAGATAGCTTCAGGGCCTCAGCCAGGACAGCAGCAGAATGGATTTACTGCTCAGCCAGCTACTTACCATCATAACAGCACTACCACCTGGACTGGAAGTAGGACTGCACCATACACACCTAATTTGCCTCACCACCAAAACGGCCATCTTCAGCACCACCCGCCTATGCCGCCCCATCCTGGACATTACTGGCCAGTTCACAATGAGCTTGCATTCCAGCCTCCCATTTCCAATCATCCTGCTCCTGAGTACTGGTGCTCCATTGCTTACTTTGAAATGGACGTTCAGGTAGGAGAGACGTTTAAGGTCCCTTCAAGCTGCCCTGTTGTGACTGTGGATGGCTATGTGGATCCTTCGGGAGGAGATCGCTTTTGCTTGGGTCAACTCTCCAATGTCCACAGGACAGAAGCGATTGAGAGAGCGAGGTTGCACATAGGCAAAGGAGTGCAGTTGGAATGTAAAGGTGAAGGTGACGTTTGGGTCAGGTGCCTTAGTGACCACGCGGTCTTTGTACAGAGTTACTACCTGGACAGAGAAGCTGGCCGAGCACCTGGCGACGCTGTTCATAAGATCTACCCAAGCGCGTATATAAAGGTCTTTGATCTGCGGCAGTGTCACCGGCAGATGCAGCAACAGGCGGCCACTGCGCAAGCTGCAGCTGCTGCTCAGGCGGCGGCCGTGGCAGGGAACATCCCTGGCCCTGGGTCCGTGGGTGGAATAGCTCCAGCCATCAGTCTGTCTGCTGCTGCTGGCATCGGTGTGGATGACCTCCGGCGATTGTGCATTCTCAGGATGAGCTTTGTGAAGGGCTGGGGCCCAGACTACCCCAGGCAGAGCATCAAGGAAACCCCGTGCTGGATTGAGATTCACCTTCACCGAGCTCTGCAGCTCTTGGATGAAGTCCTGCACACCATGCCCATTGCGGACCCACAGCCTTTAGACGGTATGGACTACAAGGATGACGATGACAAGGATTACAAAGACGACGATGATAAGGACTATAAGGATGATGACGACAAATGAGCTAGCACATAACTTACGGTAAATGGCCCGCCTGGCTGACCGCCCAACGACCCCCGCCCATTGACGTCAATAGTAACGCCAATAGGGACTTTCCATTGACGTCAATGGGTGGAGTATTTACGGTAAACTGCCCACTTGGCAGTACATCAAGTGTATCATATGCCAAGTACGCCCCCTATTGACGTCAATGACGGTAAATGGCCCGCCTGGCATTGTGCCCAGTACATGACCTTA | GV492 |
| LV-PDLIM7 | TTAGACGAAGCTTGGGCTGCAGGTCGACTCTAGAGGATCCCGCCACCATGGATTCCTTCAAGGTGGTGCTGGAAGGGCCAGCCCCTTGGGGCTTCCGTCTGCAAGGGGGCAAGGACTTCAATGTGCCCCTCTCTATCTCTCGGCTCACGCCCGGAGGCAAAGCTGCACAGGCCGGTGTGGCTGTGGGAGACTGGGTACTGAATATTGACGGTGAGAACGCGGGCAGCCTCACGCACATCGAAGCCCAGAACAAGATCCGCGCCTGTGGGGAGCGCCTCAGCCTGGGTCTTAGCAGAGCCCAGCCTGTTCAGAGCAAACCACAGAAGGCCCTGACCCCTCCCGCCGACCCCCCGAGGTACACTTTTGCACCAAGCGCCTCCCTCAACAAGACGGCCCGGCCCTTCGGGGCACCCCCACCTACTGACAGCACCCTGCGGCAGAATGGACAGTTGCTCAGACAGCCGGTCCCCGATGCCAGCAAGCAGCGGCTGATGGAGGATACCGAAGACTGGCGGCCGCGGCCGGGGACAGGCCAGTCCCGCTCCTTCCGCATCCTTGCCCACCTCACGGGCACAGAGTTCATGCAAGACCCGGATGAGGAATTCATGAAGAAGTCAAGCCAGGTGCCCAGGACAGAAGCCCCAGCCCCAGCCTCAACTATACCCCAGGAATCCTGGCCTGGCCCCACCACTCCCAGCCCCACCAGCCGCCCACCCTGGGCTGTGGATCCTGCATTTGCTGAGCGCTATGCCCCAGACAAAACCAGCACAGTGCTGACCCGGCACAGCCAGCCAGCCACACCCACGCCTCTGCAGAACCGCACCTCCATAGTGCAGGCCGCAGCTGGAGGGGGCACAGGAGGGGGCAGCAACAACGGCAAGACTCCTGTATGCCACCAGTGCCACAAGATCATCCGCGGCCGCTACCTGGTAGCACTGGGCCACGCATACCATCCCGAGGAGTTTGTGTGCAGCCAGTGTGGAAAGGTCCTGGAAGAGGGTGGCTTCTTCGAGGAGAAGGGAGCTATCTTTTGCCCCTCCTGCTATGATGTGCGCTATGCACCCAACTGTGCCAAATGCAAGAAGAAGATCACTGGAGAGATCATGCATGCTCTGAAGATGACCTGGCACGTCCATTGCTTCACCTGTGCTGCCTGCAAAACGCCAATTCGCAACAGAGCCTTTTACATGGAAGAAGGGGCCCCCTACTGCGAGCGAGACTATGAGAAGATGTTTGGCACAAAATGTCGAGGCTGTGACTTCAAGATTGATGCTGGAGACCGCTTCCTGGAAGCGCTGGGCTTCAGCTGGCATGACACATGCTTTGTTTGCGCAATATGTCAGATCAACTTGGAAGGAAAGACCTTCTACTCCAAGAAGGACAAGCCCCTCTGCAAGAGCCACGCCTTCTCTCACGTAACCGGTATGGACTACAAGGATGACGATGACAAGGATTACAAAGACGACGATGATAAGGACTATAAGGATGATGACGACAAATGAGCTAGCACATAACTTACGGTAAATGGCCCGCCTGGCTGACCGCCCAACGACCCCCGCCCATTGACGTCAATAGTAACGCCAATAGGGACTTTCCATTGACGTCAATGGGTGGAGTATTTACGGTAAACTGCCCACTTGGCAGTACATCAAGTGTATCATATGCCAAGTACGCCCCCTATTGACGTC | GV492 |
| LV-NC | http://www.genechem.com.cn/index/supports/tool_search.html?keywords=GV492 | GV492 |
|  |  |  |
| Ad-NC | Nonsense sequence | PADM-U6-shRNA-mCMV-copGFP |
| Ad-SMAD4 | Target sequence: GCGATTGTGCATTCTCAGGAT | PADM-U6-shRNA-mCMV-copGFP |
| Ad-MYCBP2 | Target sequence: GACGTCTCTAGAATTAGTTAA | PADM-U6-shRNA-mCMV-copGFP |
|  |  |  |
| ANXA1-Flag | ATGGCAATGGTATCAGAATTCCTCAAGCAGGCCCGTTTTCTTGAAAATCAAGAACAGGAATATGTTCAAGCTGTAAAATCATACAAAGGTGGTCCTGGGTCAGCAGTGAGCCCCTACCCTTCCTTCAATGTATCCTCGGATGTTGCTGCCTTGCACAAAGCTATCATGGTTAAAGGTGTGGATGAAGCAACCATCATTGACATTCTTACCAAGAGGACCAATGCTCAGCGCCAGCAGATCAAGGCCGCGTACTTACAGGAGAATGGAAAGCCCTTGGATGAAGTCTTGAGAAAAGCCCTTACAGGCCACCTGGAGGAGGTTGTTTTGGCTATGCTAAAAACTCCAGCTCAGTTTGATGCAGATGAACTCCGTGGTGCCATGAAGGGACTTGGAACAGATGAAGACACTCTCATTGAGATTTTGACAACAAGATCTAACGAACAAATCAGAGAGATTAATAGAGTCTACAGAGAAGAGCTGAAAAGAGATCTGGCCAAAGACATCACTTCAGATACATCTGGAGACTTTCGGAAAGCCTTGCTTGCTCTTGCCAAGGGTGACCGTTGTCAGGACTTGAGTGTGAATCAAGATTTGGCTGATACAGATGCCAGGGCTTTGTATGAAGCTGGAGAAAGGAGAAAGGGGACAGACGTGAACGTCTTCACCACAATTCTGACCAGCAGGAGCTTTCCTCATCTTCGCAGAGTGTTTCAGAATTACGGAAAGTACAGTCAACATGACATGAACAAAGCTCTGGATCTGGAACTGAAGGGTGACATTGAGAAGTGCCTCACAACCATCGTGAAGTGTGCCACCAGCACTCCAGCTTTCTTTGCCGAGAAGCTGTACGAAGCCATGAAGGGTGCCGGAACTCGCCATAAGGCATTGATCAGGATTATGGTCTCCCGTTCGGAAATTGACATGAATGAAATCAAAGTATTTTACCAGAAGAAGTATGGAATCTCTCTTTGCCAAGCCATCCTGGATGAAACCAAAGGAGACTATGAAAAAATCCTGGTGGCTCTGTGTGGTGGAAACGATTACAAGGATGACGACGATAAGTAG | pUC57 |
| ANXA1-pfam1-Flag | ATGGCAATGGTATCAGAATTCCTCAAGCAGGCCCGTTTTCTTGAAAATCAAGAACAGGAATATGTTCAAGCTGTAAAATCATACAAAGGTGGTCCTGGGTCAGCAGTGAGCCCCTACCCTTCCTTCAATGTATCCTCGGATGTTGCTGCCTTGCACAAAGCTATCATGGTTAAAGGTGTGGATGAAGCAACCATCATTGACATTCTTACCAAGAGGACCAATGCTCAGCGCCAGCAGATCAAGGCCGCGTACTTACAGGAGAATGGAAAGCCCTTGGATGAAGTCTTGAGAAAAGCCCTTACAGGCCACCTGGAGGAGGTTGTTTTGGCTATGGATTACAAGGATGACGACGATAAGTAG | pUC57 |
| ANXA1-pfam2-Flag | ATGCTAAAAACTCCAGCTCAGTTTGATGCAGATGAACTCCGTGGTGCCATGAAGGGACTTGGAACAGATGAAGACACTCTCATTGAGATTTTGACAACAAGATCTAACGAACAAATCAGAGAGATTAATAGAGTCTACAGAGAAGAGCTGAAAAGAGATCTGGCCAAAGACATCACTTCAGATACATCTGGAGACTTTCGGAAAGCCTTGCTTGCTCTTGCCAAGGGTGACCGTTGTCAGGACTTGAGTGTGAATCAAGATTTGGCTGATACAGATGCCAGGGCTTTGTATGAAGCTGGAGAAAGGAGAAAGGGGACAGACGTGAACGTCTTCACCACAATTCTGACCAGCAGGAGCTTTCCTCATCTTCGCAGAGTGTTTCAGAATTACGGAAAGTACAGTCAACATGACATGAACAAAGCTCTGGATCTGGAACTGAAGGGTGACATTGAGAAGTGCCTCACAACCATCGTGAAGTGTGCCACCAGCACTCCAGCTTTCTTTGCCGAGAAGCTGTACGAAGCCATGAAGGGTGCCGGAACTCGCCATAAGGCATTGATCAGGATTATGGTCTCCCGTTCGGAAATTGACATGAATGAAATCAAAGTATTTTACCAGAAGAAGTATGGAATCTCTCTTTGCCAAGCCATCCTGGATGAAACCAAAGGAGACTATGAAAAAATCCTGGTGGCTCTGTGTGGTGGAAACGATTACAAGGATGACGACGATAAGTAG | pUC57 |
| PDLIM7-HA | ATGGATTCCTTCAAGGTGGTGCTGGAAGGGCCAGCCCCTTGGGGCTTCCGTCTGCAAGGGGGCAAGGACTTCAATGTGCCCCTCTCTATCTCTCGGCTCACGCCCGGAGGCAAAGCTGCACAGGCCGGTGTGGCTGTGGGAGACTGGGTACTGAATATTGACGGTGAGAACGCGGGCAGCCTCACGCACATCGAAGCCCAGAACAAGATCCGCGCCTGTGGGGAGCGCCTCAGCCTGGGTCTTAGCAGAGCCCAGCCTGTTCAGAGCAAACCACAGAAGGCCCTGACCCCTCCCGCCGACCCCCCGAGGTACACTTTTGCACCAAGCGCCTCCCTCAACAAGACGGCCCGGCCCTTCGGGGCACCCCCACCTACTGACAGCACCCTGCGGCAGAATGGACAGTTGCTCAGACAGCCGGTCCCCGATGCCAGCAAGCAGCGGCTGATGGAGGATACCGAAGACTGGCGGCCGCGGCCGGGGACAGGCCAGTCCCGCTCCTTCCGCATCCTTGCCCACCTCACGGGCACAGAGTTCATGCAAGACCCGGATGAGGAATTCATGAAGAAGTCAAGCCAGGTGCCCAGGACAGAAGCCCCAGCCCCAGCCTCAACTATACCCCAGGAATCCTGGCCTGGCCCCACCACTCCCAGCCCCACCAGCCGCCCACCCTGGGCTGTGGATCCTGCATTTGCTGAGCGCTATGCCCCAGACAAAACCAGCACAGTGCTGACCCGGCACAGCCAGCCAGCCACACCCACGCCTCTGCAGAACCGCACCTCCATAGTGCAGGCCGCAGCTGGAGGGGGCACAGGAGGGGGCAGCAACAACGGCAAGACTCCTGTATGCCACCAGTGCCACAAGATCATCCGCGGCCGCTACCTGGTAGCACTGGGCCACGCATACCATCCCGAGGAGTTTGTGTGCAGCCAGTGTGGAAAGGTCCTGGAAGAGGGTGGCTTCTTCGAGGAGAAGGGAGCTATCTTTTGCCCCTCCTGCTATGATGTGCGCTATGCACCCAACTGTGCCAAATGCAAGAAGAAGATCACTGGAGAGATCATGCATGCTCTGAAGATGACCTGGCACGTCCATTGCTTCACCTGTGCTGCCTGCAAAACGCCAATTCGCAACAGAGCCTTTTACATGGAAGAAGGGGCCCCCTACTGCGAGCGAGACTATGAGAAGATGTTTGGCACAAAATGTCGAGGCTGTGACTTCAAGATTGATGCTGGAGACCGCTTCCTGGAAGCGCTGGGCTTCAGCTGGCATGACACATGCTTTGTTTGCGCAATATGTCAGATCAACTTGGAAGGAAAGACCTTCTACTCCAAGAAGGACAAGCCCCTCTGCAAGAGCCACGCCTTCTCTCACGTATACCCATACGACGTCCCAGACTACGCTTAG | pUC57 |
| PDLIM7-pfam1-HA | ATGGATTCCTTCAAGGTGGTGCTGGAAGGGCCAGCCCCTTGGGGCTTCCGTCTGCAAGGGGGCAAGGACTTCAATGTGCCCCTCTCTATCTCTCGGCTCACGCCCGGAGGCAAAGCTGCACAGGCCGGTGTGGCTGTGGGAGACTGGGTACTGAATATTGACGGTGAGAACGCGGGCAGCCTCACGCACATCGAAGCCCAGAACAAGATCCGCGCCTGTGGGGAGCGCCTCAGCCTGGGTCTTAGCAGAGCCCAGCCTGTTCAGAGCAAACCACAGAAGGCCCTGACCCCTCCCGCCGACCCCCCGAGGTACACTTTTGCACCAAGCGCCTCCCTCAACAAGACGGCCCGGCCCTTCGGGGCACCCCCACCTACTGACAGCACCCTGCGGCAGAATGGACAGTTGCTCAGACAGCCGGTCCCCGATGCCAGCAAGCAGCGGCTGATGGAGGATACCGAAGACTGGCGGCCGCGGCCGGGGACAGGCCAGTCCCGCTCCTTCCGCATCCTTGCCCACCTCACGGGCACAGAGTTCATGCAAGACCCGGATGAGGAATTCTACCCATACGACGTCCCAGACTACGCTTAG | pUC57 |
| PDLIM7-pfam2-HA | ATGAAGAAGTCAAGCCAGGTGCCCAGGACAGAAGCCCCAGCCCCAGCCTCAACTATACCCCAGGAATCCTGGCCTGGCCCCACCACTCCCAGCCCCACCAGCCGCCCACCCTGGGCTGTGGATCCTGCATTTGCTGAGCGCTATGCCCCAGACAAAACCAGCACAGTGCTGACCCGGCACAGCCAGCCAGCCACACCCACGCCTCTGCAGAACCGCACCTCCATAGTGCAGGCCGCAGCTGGAGGGGGCACAGGAGGGGGCAGCAACAACGGCAAGACTCCTGTATGCCACCAGTGCCACAAGATCATCCGCGGCCGCTACCTGGTAGCACTGGGCCACGCATACCATCCCGAGGAGTTTGTGTGCAGCCAGTGTGGAAAGGTCCTGGAAGAGGGTGGCTTCTTCGAGGAGAAGGGAGCTATCTTTTGCCCCTCCTGCTATGATGTGCGCTATTACCCATACGACGTCCCAGACTACGCTTAG | pUC57 |
| PDLIM7-pfam3-HA | ATGGCACCCAACTGTGCCAAATGCAAGAAGAAGATCACTGGAGAGATCATGCATGCTCTGAAGATGACCTGGCACGTCCATTGCTTCACCTGTGCTGCCTGCAAAACGCCAATTCGCAACAGAGCCTTTTACATGGAAGAAGGGGCCCCCTACTGCGAGCGAGACTATGAGAAGATGTTTTACCCATACGACGTCCCAGACTACGCTTAG | pUC57 |
| PDLIM7-pfam4-HA | ATGGGCACAAAATGTCGAGGCTGTGACTTCAAGATTGATGCTGGAGACCGCTTCCTGGAAGCGCTGGGCTTCAGCTGGCATGACACATGCTTTGTTTGCGCAATATGTCAGATCAACTTGGAAGGAAAGACCTTCTACTCCAAGAAGGACAAGCCCCTCTGCAAGAGCCACGCCTTCTCTCACGTATACCCATACGACGTCCCAGACTACGCTTAG | pUC57 |
| RING-Flag | ATGGGAAAGGACGGGCAACAGAAGCAAATGCCTATGTGTGACAACCATGATGACGGTGAAACTGCAGCTATCATCTTATGCAACATCTGTGGCAATCTGTGTACTGACTGTGACAGATTCCTTCATCTGCATCGACGAACGAAGACTCACCAAAGACAGGTCTTCAAAGAAGAAGAAGAGGCTATAAAGGTCGACCTCCATGAAGGCTGTGGTAGAACAAAACTGTTCTGGTTGATGGCATTAGCAGATTCTAAAACAATGAAGGCAATGGTGGAATTCCGAGAACACACAGGCAAACCCACCACGAGTAGCTCGGAAGCATGCCGCTTCTGTGGGTCCAGGAGTGGAACGGAGCTGTCTGCTGTTGGCAGTGTTTGTTCTGATGCAGACTGCCAGGAATATGCTAAGATCGCCTGCAGTAAGACACATCCCTGCGGACATCCGTGTGGAGGTGTTAGAAACGAAGAGCACTGCCTGCCCTGCCTGCACGGCTGTGATAAGAGCGCCACCACACTGAAACAAGATGCCGACGACATGTGCATGATCTGCTTCACCGAAGCGCTCTCTGCAGCACCGGCCATTCAGTTGGACTGCAGTCATGTGTTCCACTTACAGTGCTGTCGGCGAGTTTTGGAAAACAGATGGCTTGGTCCAAGGATAACGTTTGGATTCATTTCGTGTCCTATTTGCAAGAACAAGATAAATCATATAGTATTAAAAGACCTGCTTGATCCAATAAAAGAGCTCTATGAAGACGTCAGAAGAAAAGCCTTAATGAGATTGGAGTATGAAGGTCTGCATAAGAGTGAAGCTATCACGACTCCTGGTGTCAGGTTTTATAATGATGCAGCTGGCTATGCCATGAATAGATACGCATATTATGTCTGCTACAAGTGCAGAAAGGCATACTTTGGTGGTGAGGCTCGCTGTGATGCTGAGGCTGGACAAGGAGATGATTACGACCCCAGAGAGCTCATCTGCGGCGCCTGTTCCGATGTGTCCAGGGCCCAGATGTGTCCCAAACATGGAACAGACTTTCTAGAATACAAGTGTCGCTACTGCTGTTCGGTGGCTGTCTTCTTCTGTTTCGGAACAACACATTTCTGTAATGCTTGTCATGATGACTTTCAGAGAATGACCAGCATTCCTAAGGAAGAACTCCCACACTGTCCTGCAGGTCCCAAAGGCAAGCAGCTAGAAGGAACTGAGTGTCCACTCCACGTTGTTCATCCGCCCACGGGGGAAGAGTTTGCTCTCGGGTGCGGCGTGTGCAGAAATGCTCACACGTTTGATTACAAGGATGACGACGATAAGTAG | pUC57 |

Supplementary Table 3

| siRNA name | sense（5'-3'） | antisense（5'-3'） |
| --- | --- | --- |
| Negative control | UUCUCCGAACGUGUCACGUTT | ACGUGACACGUUCGGAGAATT |
| siRNA-ANXA1 | CCAUCCUGGAUGAAACCAATT | UUGGUUUCAUCCAGGAUGGTT |
| siRNA-ANXA1(1) | GGACUUGAGUGUGAAUCAATT | UUGAUUCACACUCAAGUCCTT |
| siRNA-SMAD4 | CAGUAUGCGUUUGACUUAATT | UUAAGUCAAACGCAUACUGTT |
| siRNA-SMAD4(1) | CUCCAUUGCUUACUUUGAATT | UUCAAAGUAAGCAAUGGAGTT |
| siRNA-PDLIM7 | GAGACUGGGUACUGAAUAUTT | AUAUUCAGUACCCAGUCUCTT |
| siRNA-PDLIM7(1) | GGCAGCAACAACGGCAAGATT | UCUUGCCGUUGUUGCUGCCTT |
| siRNA-MYCBP2 | CAGGCUCUGUGAGGUUUGATT | UCAAACCUCACAGAGCCUGTT |
| siRNA-MYCBP2(1) | CCGGCCUCCAGGAAUAAGATT | UCUUAUUCCUGGAGGCCGGTT |

Supplementary Table 4

| Gene name | Forward | Reverse |
| --- | --- | --- |
| m-β-actin | CATTGCTGACAGGATGCAGAAGG | TGCTGGAAGGTGGACAGTGAGG |
| m-Anxa1 | CCTCATCTTCGCAGAGTGTTTC | ACGATGGTTGTGAGGCACTTCT |
| m-Fasn | TTGCTGGCACTACAGAATGC | AACAGCCTCAGAGCGACAAT |
| m-Acc1 | ATTGGGCACCCCAGAGCTA | CCCGCTCCTTCAACTTGCT |
| m-Acc2 | GGGCTCCCTGGATGACAAC | TTCCGGGAGGAGTTCTGGA |
| m-Scd1 | TTCTTGCGATACACTCTGGTGC | CGGGATTGAATGTTCTTGTCGT |
| m-ChREBP | ACCTGTCTCCCCCTCAAACT | TGTCTTCTGAAGCGTGGTTG |
| m-SREBP1c | GATGTGCGAACTGGACACAG | CATAGGGGGCGTCAAACAG |
| m-Ppara | GCAGCTCGTACAGGTCATCA | CTCTTCATCCCCAAGCGTAG |
| m-Pparg | GATGTCTCACAATGCCATCAG | ATATCACTGGAGATCTCCGC |
| m-Ppard | CTCTTCATCGCGGCCATCATTCT | TCTGCCATCTTCTGCAGCAGCTT |
| m-Adipoq | AGATGCAGGTCTTCTTGGTC | ACATAAGCGGCTTCTCCAG |
| m-Plin1 | GAGAAGGGTGTACAGGGTG | CATTGGCAGCTGTGAACTG |
| m-Fabp4 | CAGAAGTGGGATGGAAAGTC | GCCTTTCATAACACATTCCAC |
| m-Cebpa | ACGAGACGTCTATAGACATCAG | AGGAACTCGTCGTTGAAGG |
| m-Cidec | CCTCCTGAACAAGGTCCAG | ACGATTGTGCCATCTTCCT |
| m-Smad4 | CTCCAGCCATCAGTCTGTC | CAAAGCTCATCCTGAGAATGC |
| m-Pdlim7 | CAGAGCAAACCACAGAAGG | GAGCAACTGTCCATTCTGC |
| m-Mycbp2 | ATGGCCTTAAGATGCTGGA | GATGTGATGGAAGACATTGCT |
| m-IL6 | AAGCCAGAGTCCTTCAGAGAGA | ACTCCTTCTGTGACTCCAGCTT |
| m-Ccl2 | CCACAACCACCTCAAGCACTTC | AAGGCATCACAGTCCGAGTCAC |
| m-Cxcl10 | CTCAGGCTCGTCAGTTCTAAGT | CCCTTGGGAAGATGGTGGTTAA |
|  |  |  |
| m-Anxa1-flox | ATCAGCTCTTCTCCTCAGGTCC | TGGTGCAGCATAAAATGTGTGGTG |
| m-Adipoq-Cre | GGATGTGCCATGTGAGTCTG | ACGGACAGAAGCATTTTCCA |
| m-Anxa1-Tg | CAAAGCTCTGGATCTGGAACTG | GACGGAGCTGGGTTAGGTATG |
|  |  |  |
| m-Pparg-pro | GACATGGACATGGACATCGG | TGAGGAAATCCTTTCCCCCATC |
|  |  |  |
| h-β-actin | GAAGATCAAGATCATTGCTCCTC | ATCCACATCTGCTGGAAGG |
| h-ANXA1 | GCAGGAATATGTTCAAACTGTG | GATGGATTGAAGGTAGGATAGG |
